# Synthesis of bis(aryloxy)fluoromethanes using a heterodihalocarbene strategy

**DOI:** 10.3762/bjoc.17.70

**Published:** 2021-04-12

**Authors:** Carl Recsei, Yaniv Barda

**Affiliations:** 1 ADAMA Agan, HaAshlaag 3, Northern Industrial Zone, Ashdod, 77102, Israel

**Keywords:** acetal, aryloxyfluoromethane, dihalocarbene, herbicide, organofluorine

## Abstract

A side-product present in the herbicide pyroxasulfone was synthesized. The construction of a bis(aryloxy)fluoromethane moiety was necessary, for which no existing method was available. We report a simple, new procedure which we applied to the synthesis of some of these unusual structures.

## Introduction

Organofluorine molecules are widely used for medicinal, agrochemical and material purposes. Recently, a proliferation of methods has allowed easy access to a particularly desired class of these compounds: singly fluorinated compounds such as fluoromethyl ethers [1]. Still missing from the chemist’s toolkit, however, are means to construct their more highly oxidized analogues, fluoromethylene acetals.

**Scheme 1:**
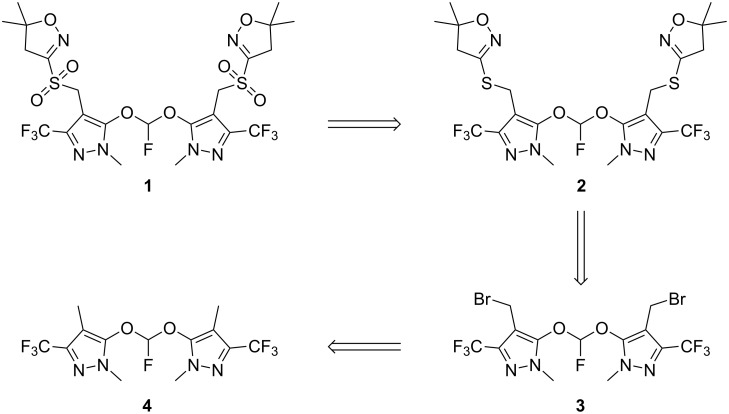
Retrosynthesis of compound **1**.

In the course of preparing synthetic samples of trace impurities in the herbicide pyroxasulfone, we were confronted with the problem of generating a bis(aryloxy)fluoromethane. Our intended route to structure **1** (Scheme 1) required the oxidation of bis-thioether **2**, derived in turn from dibromide **3**, produced by radical bromination of key intermediate **4**.

The principal challenge entailed in the envisaged synthesis was the construction of the bis(aryloxy)fluoromethane moiety of **4**. The simplicity of this structure belies the paucity with which it is encountered in the literature, particularly in an acyclic form. Reports of this functional group occurring in trace quantities as byproducts exist [2–5], but we are aware of only two examples of a bis(aryloxy)fluoromethane being reported as the desired product of a chemical transformation.

**Scheme 2:**
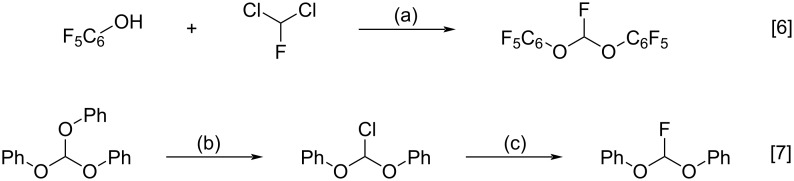
Reported bis(aryloxy)fluoromethane syntheses. Reagents and conditions: (a) Cl_2_FCH, NaOH, 1,4-dioxane/water, 70 °C, 20 min, 90% (+3% (C_6_F_5_)_3_CH); (b) AcCl, HCl, Et_2_O, rt, 24 h, 70%; (c) KHF_2_, neat, 105–110 °C, 60%.

The first example of a reported bis(aryloxy)fluoromethane synthesis is a 1973 report by Platonov of the reaction of a slight excess of dichlorofluoromethane with pentafluorophenol in the presence of base, presumably via chlorofluorocarbene as an intermediate (Scheme 2) [6]. Typically, phenoxides are known to react with difluorocarbene to form (difluoromethoxy)arenes, while the reaction with dichlorocarbene gives salicylaldehydes via hydrolysis of an *ortho*-dichloromethylphenol: the Reimer–Tiemann reaction. The literature, other than this single reference with pentafluorophenol [6], is bereft of references to the capture of heterodihalocarbenes by phenol nucleophiles.

The second report, by Scheeren [7], working from a bis(phenoxy)chloromethane synthesis reported by Scheibler [8], described the conversion of phenyl orthoformate to bis(phenoxy)chloromethane with acetyl chloride and anhydrous hydrogen chloride, followed by reaction with potassium hydrogendifluoride to give bis(phenoxy)fluoromethane (Scheme 2) [7]. We initially considered this route impractical for our purposes since, according to the author: ‘*The procedure is restricted to the preparation of diaryloxymethyl chlorides in which the aryl residues do not contain more or stronger electron-withdrawing groups than one chloro substituent’* [7].

Fearing instability of a hypothetical bis(aryloxy)chloromethane intermediate, we initially anticipated that the number of literature reports detailing the isolation of bis(aryloxy)fluoromethanes as byproducts [2–5], albeit in very small amounts, might serve better our need to produce the structure **1** (Scheme 1), rather than the literature example with electron-poor pentafluorophenol and chlorofluorocarbene [6].

## Results and Discussion

### Synthesis of compound **4**

**Scheme 3:**
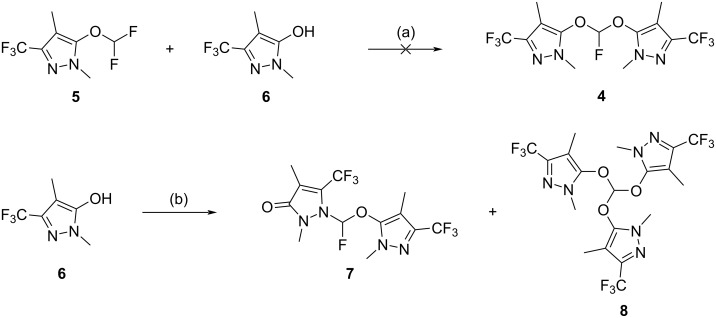
Attempted synthesis of **4**. Reagents and conditions: (a) Ca(OH)_2_, 1,4-dioxane/water, reflux, 72 h, 5% (by HPLC integration); (b) ClF_2_CO_2_Na, K_2_CO_3_, DMF, 95 °C, 4 h, **7** (4%), **8** (19%).

Reports of bis(aryloxy)fluoromethanes as side products in difluoromethylation reactions led us to attempt to displace a single fluoride ion from **5** with the anion of **6** (Scheme 3), with fluorophilic calcium hydroxide as a base, in analogy to a report where phenoxide moieties are introduced to the anomeric position of a fluorinated sugar [9]. This gave only traces of **4**, while adjustments in reactant ratios, the identity of the base and admixture of potential catalysts such as tetrabutylammonium iodide or DABCO showed no improvement in product ratios. Using superstoichiometric **6** and base with chlorodifluoromethane in a bid to form and react **5** in a one-pot protocol were unsuccessful, even after a screen of bases, reactant ratios, reactor pressure, addition rates and temperature.

A compound with the requisite molecular formula was produced as a minor product by the reaction of **6** (Scheme 3) with difluorocarbene generated at elevated temperature via decarboxylation of chlorodifluoroacetate, but it was found to be the isomeric material **7**, with no trace of the desired isomer. It was also apparent that where the reaction did succeed in producing small quantities of **4**, it was converted to the *ortho-*ester **8** at a rate which precluded generation of **4** in greater quantities than **8**. Yields of compound **4** were below 5% for this type of transformation. Our conclusion was that the attempt to produce the desired structure **4** in reasonable yield by adapting literature reports which detailed the production of a bis(aryloxy)fluoromethane as a side product had been unsuccessful.

Having obtained compound **8**, we revisited Scheeren’s preparation of bis(phenoxy)chloromethane from phenyl orthoformate [7] and attempted to apply the method to the production of compound **4**. The intermediate bis(aryloxy)chloromethane was not observed, even after heating. We eschewed attempts to produce this bis(aryloxy)chloromethane via a published protocol for radical chlorination of an acetal due to the presence of vulnerable benzylic methyl groups proximate to the acetal [10].

**Scheme 4:**
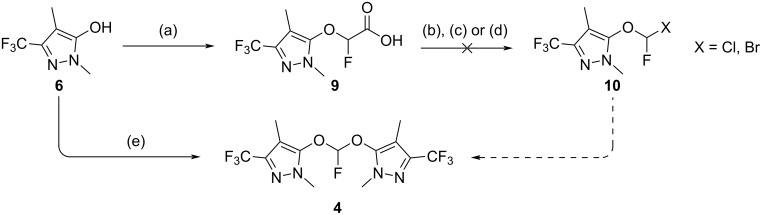
Synthesis of **10**. Reagents and conditions: (a) BrFCHCO_2_Et, Cs_2_CO_3_, DMF, 35 °C, 16 h then H_2_O, 35 °C, 4 h, 80%; (b) BrCCl_3_, DMAP, *N*-hydroxy-2-thiopyridone sodium salt, *h*ν, CH_2_Cl_2_, rt, 16 h then Bu_4_N^+^Cl^−^, rt, 3 h, 0%; (c) dibromoisocyanuric acid, Bu_4_N^+^Br_3_^−^, *h*ν, CH_2_Cl_2_, rt, 24 h, 0%; (d) SOCl_2_, DMF, 70 °C, 16 h, 0%; (e) Br_2_FCH, K_3_PO_4_, MeCN, rt, 3 d, 39%.

We then synthesized carboxylic acid **9**, from which we anticipated creating an aryloxylchlorofluoromethane (**10**, X = Cl, Scheme 4) via chlorodecarboxylation. This reaction would be analogous to the single reported synthesis of bis(pentafluorophenoxy)fluoromethane, in that it would be a route to the product of the reaction between an aryl oxide and chlorofluorocarbene. Our conjecture was that the ease by which compound **10** could be transformed into **4** (by direct attack of a aryl oxide nucleophile or deprotonation and loss of chloride in a second carbene generation followed by a second phenoxide attack) would be substantially greater than an aryloxydifluoromethane such as **5**, with milder conditions allowing us to avoid the undesired isomer **7**. Unfortunately, several methods of halodecarboxylation [11–13] failed to yield the desired product **10** (X = Cl, Br), likely due to the presence, in the pyrazole, of moieties sensitive to these conditions or to the instability of the product to the reaction conditions.

Pursuing the heterodihalocarbene strategy, we were delighted to observe that the desired compound, **4**, was produced as the major product, in 39% yield (72% yield based on recovered starting material) when **6** was reacted with dibromofluoromethane in the presence of excess potassium phosphate in acetonitrile (Scheme 4). Smaller amounts of the undesired isomer were produced, recovery of unreacted **6** was simple and the quantity of **8** (<5%) was reduced compared to all previous attempts. Chromatographic separation permitted the removal of **8** and the isolation of a pure sample of compound **4**.

### Synthesis of compound **1**

**Scheme 5:**
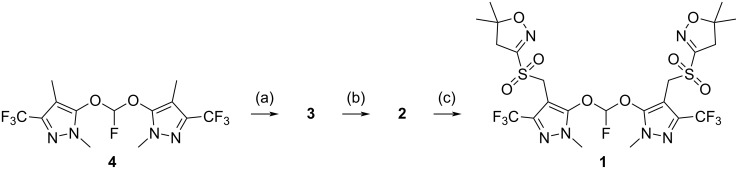
Synthesis of **1**. Reagents and conditions: (a) 1,3-dibromo-5,5-dimethylhydantoin, benzoyl peroxide, (CH_2_Cl)_2_, reflux, 4 h, 88%; (b) 5,5-dimethyl-3-(4*H*-isoxazolyl) carbamimidothioate·HCl, K_2_CO_3_, MeCN/H_2_O, 50 °C, 1.5 h, 73%; (c) H_2_O_2_, Na_2_WO_4_, MeCN/H_2_O, 45 °C, 1.5 d, 66%.

Having produced the key intermediate **4**, we completed the radical bromination of **4** to produce **3** (Scheme 5), which was used to deliver the penultimate bis-thioether **2**, with oxidation to **1** giving the target impurity structure as anticipated, with a combined yield for the three transformations from **4** to **1** of 42%.

The synthesis of **4** was also possible with potassium hydroxide as a base, while potassium carbonate gave only traces of the desired product. DMF was found to be an inferior solvent. All these changes led to larger quantities of the undesired isomer **7**, with respect to **4**. Attempting to push this reaction to higher conversion of **6** with an increase in the amount of dibromofluoromethane employed, also disturbed the product ratio in favor of the undesired isomer **7**.

### A general method to produce electron-poor acyclic bis(aryloxy)fluoromethanes

**Scheme 6:**
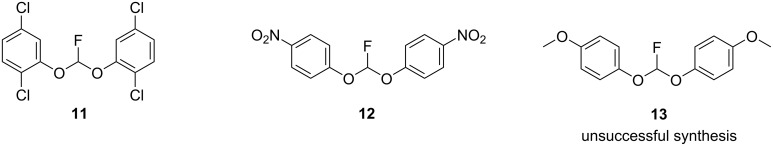
Synthesis of **11**–**13**. Reagents and conditions: ArOH (1.3 mmol), Br_2_FCH (1.3 mmol), KOH (4 mmol), MeCN (5 mL), rt, 16 h, 54% (68%*) (**11**), 52% (64%*) (**12**), 0%* (**13**). *Isolated yields based on recovered starting material.

We had produced the unusual acyclic bis(aryloxy)fluoromethane moiety and some of the limitations that we had encountered in terms of yield and regioselectivity were presumably specific to the ambident nucleophile **6**. We considered that a different ambident nucleophile, a phenol, might also give unusual selectivity for production of a bis(aryloxy)fluoromethane over a Reimer–Tiemann formylation. Indeed, we discovered that we could react 2,5-dichlorophenol with dibromofluoromethane, in the presence of potassium hydroxide, at ambient temperature, to give **11** in 54% yield, or 68% based on recovered starting material (Scheme 6). The examination of the crude product mixture by NMR showed that no aldehydes were present, and that unreacted phenol comprised a further 26% of the mass balance. Thus, the reactivity of fluorobromocarbene in this case mirrored the tendency of difluorocarbene to react with phenoxides to form dihalomethyl phenyl ethers, rather than forming aldehydes as with dichlorocarbene – the classic Reimer–Tiemann intermediate. This was the case even after the addition of water and continued stirring to allow time for a hypothetical intermediate (bromofluoromethyl)arene to hydrolyze.

A general procedure for the production of electron-poor bis(aryloxy)fluoromethanes is to stir equimolar phenol and dibromofluoromethane with 3 equivalents of potassium hydroxide in acetonitrile at ambient temperature in a sealed flask for 16 hours. Using this method, we produced an additional compound, **12** (Scheme 6). The yield of **11** from the corresponding phenol may be increased to 78% with three equivalents of dibromofluoromethane, yet this rather expensive reagent is preferably not used in such a large excess, since phenol recovery is trivial under these conditions. Using *p*-methoxyphenol we observed a complex reaction mixture, in which the predominant product was the *ortho*-ester. Although a peak of the corresponding mass was observed by GC–MS, we were unable to isolate **13** in pure form.

The base-mediated method we have reported herein is complementary to that of Scheeren, since it is apparently more practical for electron-poor arenes, while the bis(aryloxy)chloromethanes required as intermediates in Scheeren’s method must be constructed with electron-rich arenes under highly acidic conditions. Furthermore, the method detailed in this work is operationally simple, not requiring the use or isolation of unstable intermediates and giving electron-poor bis(aryloxy)fluoromethanes in a single step.

**Scheme 7:**
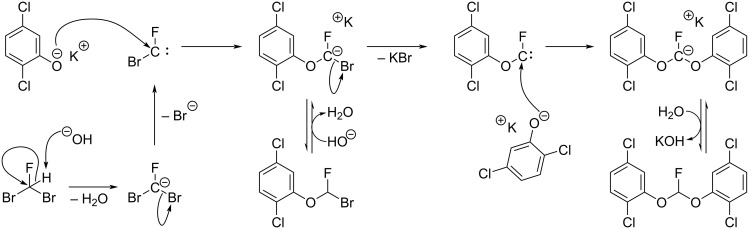
Proposed mechanism for the formation of compound **11**.

An attempt to react dibromofluoromethane with *n*-pentanol in the presence of potassium hydroxide or sodium *tert*-butoxide did not produce di(*n*-pentoxy)fluoromethane at ambient temperature. Since elevated temperatures are typically required for the direct, S_N_2 attack upon dibromomethanes by alcohols or phenols [14], our results suggest that sequential phenoxide ion attacks on intermediate carbenes (Scheme 7) was indeed the mechanism responsible for the production of compounds **4**, **11** and **12**.

The bis(aryloxy)fluoromethane moiety had proven robust with respect to radical, brominative conditions as well as highly oxidizing and alkaline conditions in the synthesis of **1**. We decided to probe the stability of this moiety to acidic conditions. Compound **11** proved to have considerable stability to acidic hydrolysis, suffering only 33% hydrolysis upon stirring in 2 M HCl/MeOH/1,2-dichloroethane 1:4:1 at 35 °C for 5 h, with 85% hydrolysis observed after 20 hours. The relative stability of **11** to acidic hydrolysis and its presumably enhanced lipophilicity with respect to a des-fluoro acetal, might presage a role for compounds possessing the acyclic bis(aryloxy)fluoromethane moiety in medicinal or agrochemical applications.

## Conclusion

We have synthesized the trace impurity **1**, using a novel and operationally simple procedure for the construction of acyclic bis(aryloxy)fluoromethanes and extended the transformation to two representative electron-poor phenols. We were able to provide preliminary answers to the previously unaddressed question of how heterodihalocarbenes would react with phenols capable of undergoing Reimer–Tiemann formylation.

## Supporting Information

File 1 Experimental procedures.

File 2 Copies of NMR spectra.
