# Supplementary material for: Synthesis of bis(aryloxy)fluoromethanes using a heterodihalocarbene strategy
Source: Beilstein J Org Chem. 2021 Apr 12;17:813–8. doi: 10.3762/bjoc.17.70 (PMC8056069; doi:10.3762/bjoc.17.70)
Supplement: File 1 — Experimental procedures. [file Beilstein_J_Org_Chem-17-813-s001.pdf]

BEILSTEIN JOURNAL OF ORGANIC CHEMISTRY

Supporting Information

for

Synthesis of bis(aryloxy)fluoromethanes using a heterodihalocarbene strategy

Carl Recsei and Yaniv Barda

Beilstein J. Org. Chem. **2021,** *17,* 813–818. doi:10.3762/bjoc.17.70

Experimental procedures

License and Terms: This is a supporting information file under the terms of the Creative Commons Attribution License (https://creativecommons.org/licenses/by/4.0). Please note that the reuse, redistribution and reproduction in particular requires that the author(s) and source are credited and that individual graphics may be subject to special legal provisions.
The license is subject to the *Beilstein Journal of Organic Chemistry* terms and conditions: (https://www.beilstein-journals.org/bjoc/terms)

Table of Contents:

	Item	*Page*
I	General experimental	S2
II	Synthesis of compound **4** (+ compounds **8** and **7**)	S3
III	Synthesis of compound **3**	S5
IV	Synthesis of compound **2**	S6
V	Synthesis of compound **1**	S7
VI	Unsuccessful reaction of **5** and **6**	S8
VII	Unsuccessful reaction of **6** and ClF_2CO_2Na	S8
VIII	Synthesis of compound **9** (+ compound **9** ethyl ester)	S9
IX	General procedure for the synthesis of bis(aryloxy)fluoromethanes	S10
X	Compound **11**	S11
XI	Compound **12**	S11
XII	Synthesis of compound **4** by the general procedure	S12

I. General experimental

All solvents and reagents were used as received from commercial sources with recrystallization or distillation performed as required. Compounds **5** and **6** were prepared in a manner analogous to Nakatani, M.; Ito, M.; Miyazaki, M. Preparation of pyrazole derivatives as intermediates in the production of herbicidal isoxazoline derivatives. WO2004013106, 2004. Preparation of 5,5-dimethyl-3-(4*H*-isoxazolyl) carbamimidothioate hydrochloride was in a manner analogous to Boehmer, J. E.; McLachlan, M. M. W. Herbicidal isoxazoline compounds, their preparation, herbicidal compositions, and their use. WO2007096576, 2007.

NMR spectra were recorded at 400, 500 or 700 MHz (^{1}H frequencies). ^{1}H chemical shifts are expressed as parts per million (ppm) with residual solvent or tetramethylsilane (δ 0.00) as reference and are reported as chemical shift, (relative integral, multiplicity, coupling constant). Multiplicities are described by the following abbreviations: s = singlet, d = doublet, t = triplet, q = quartet, dd = doublet of doublets and m = multiplet. Coupling constants (*J*) are reported in Hz. Other nuclei (^{13}C, ^{19}F and ^{15}N) are similarly reported as chemical shift, (relative integral/assignment, multiplicity, coupling constant). Chemical shifts are expressed as parts per million (ppm) with residual solvent, external nitromethane (^{15}N only) or tetramethylsilane (δ 0.00) as reference. ^{13}C NMR chemical shifts are proton decoupled. High Resolution mass spectra were obtained using a Thermo Scientific™ Exactive™ Plus Orbitrap Mass Spectrometer. Melting points were determined with a Büchi® B-545 melting point apparatus. Infrared spectra were acquired neat on an ATR spectrometer. Absorption maxima are expressed in wavenumbers (cm^{-1}).

II. Synthesis of compound **4**

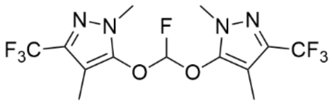

5,5'-((fluoromethylene)bis(oxy))bis(1,4-dimethyl-3-(trifluoromethyl)-1*H*-pyrazole)

Compound **6** (5.00 g, 28 mmol, 1.0 equiv) was taken up in dry MeCN (100 mL) and K_3PO_4 (18.3 g, 86 mmol, 3.0 equiv) added. Dibromofluoromethane (2.4 mL, 30 mmol, 1.1 equiv) was added. The mixture was stirred in a sealed flask (3 days). The mixture was then filtered and most of the MeCN removed in vacuo. The residue was partitioned between ethyl acetate (100 mL) and water (300 mL) and the ethyl acetate partition washed with water (100 mL), brine (100 mL), dried over $MgSO_4$ and the solvent removed in vacuo to give 5.1 g of a colorless oil. Flash column chromatography (ethyl acetate 10%, hexanes 80%, CH_2Cl_2 10%) gave compound **4** as a colorless oil (2.11 g, 39%, 72% based on recovered starting material); ^{1}H NMR (400 MHz, $CDCl_3$) 6.38 (1 H, d, $^2J_{H\text{-}F}$ 74), 3.75 (6 H, s), 2.08 (6 H, s); ^{19}F NMR (376 MHz, $CDCl_3$) -63.08 (6 F, s), -82.38 (1 F, d, $^2J_{H\text{-}F}$ 74); ^{13}C NMR (101 MHz, $CDCl_3$) 143.77 (2 C, s), 139.31 (2 C, q, $^2J_{C\text{-}F}$ 37), 121.33 (2 CF_3, q, $^1J_{C\text{-}F}$ 268), 115.44 (1 CHF, d, $^1J_{C\text{-}F}$ 264), 103.58 (2 C, s), 35.55 (2 CH_3, s), 6.56 (2 CH_3, s); IR (neat) ν_{max}/cm^{-1} 1590, 1485, 1391, 1273, 1166, 1115, 1057, 729 HRMS (ESI) obs'd 391.1004 (MH^+ = $C_{13}H_{14}F_7N_4O_2^+$) calc'd 391.1005.

The aqueous partition of the reaction mixture was acidified to pH 1 with the slow addition of 5 M HCl, then extracted with ethyl acetate (2 × 100 mL) and the ethyl acetate fractions combined, washed with water (100 mL), brine (100 mL), dried over $MgSO_4$ and the solvent removed in vacuo to permit recovery of compound **6** (2.29 g, 46% recovery).

Two additional compounds were obtained by further elution from the column:

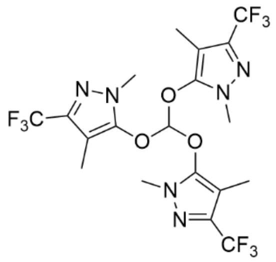

tris((1,4-dimethyl-3-(trifluoromethyl)-1*H*-pyrazol-5-yl)oxy)methane

Compound **8** (0.22 g, 4%) was eluted with ethyl acetate 15%, hexanes 75%, CH_2Cl_2 10%; colorless crystals; mp 90-92 °C; ^{1}H NMR (400 MHz, $CDCl_3$) 5.95 (1 H, s), 3.74 (9 H, s), 1.83 (9 H, s); ^{19}F NMR (376 MHz, $CDCl_3$) -63.26 (9 F, s); ^{13}C NMR (126 MHz, $CDCl_3$) 144.76 (3 C, s), 139.54 (3 C, q, $^2J_{C\text{-}F}$ 37), 121.12 (3 CF_3, q, $^1J_{C\text{-}F}$ 270), 117.01 (1 C, s), 102.72 (3 C, s), 35.52 (3 CH_3, s), 6.05 (3 CH_3, s); IR (neat) ν_{max}/cm^{-1} 1285, 1273, 1160, 1115, 1040, 1012, 725; HRMS (ESI) obs'd 551.1456 ($MH^+ = C_{19}H_{20}F_9N_6O_3^+$) calc'd 551.1453.

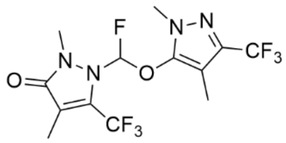

1-(((1,4-dimethyl-3-(trifluoromethyl)-1*H*-pyrazol-5-yl)oxy)fluoromethyl)-2,4-dimethyl-5-(trifluoromethyl)-1,2-dihydro-3*H*-pyrazol-3-one

Compound **7** (0.28 g, 5%) was eluted with ethyl acetate 30%, hexanes 60%, CH_2Cl_2 10%; colorless oil; ^{1}H NMR (500 MHz, $CDCl_3$) 6.53 (1 H, d, $^2J_{H\text{-}F}$ 74), 3.75(3 H, s), 3.63 (3 H, s), 2.11 (3 H, q, $^5J_{H\text{-}F}$, 2.1), 2.08 (3 H, s); ^{19}F NMR (376 MHz, $CDCl_3$) -59.93 (3 F, s), -63.27 (3 F, s), -102.76 (1 F, d, $^2J_{H\text{-}F}$ 60); ^{13}C NMR (126 MHz, $CDCl_3$) 166.09 (1 C, s), 145.77 (1 C, s), 139.35 (1 C, q, $^2J_{C\text{-}F}$ 37), 136.40 (1 C, q, $^2J_{C\text{-}F}$ 37), 121.81 (1 C, s), 121.39 (1 CF_3, q, $^1J_{C\text{-}F}$ 270), 119.94 (1 CF_3, q, $^1J_{C\text{-}F}$ 274), 109.23 (1 CHF, dd, *J* 264, 2), 102.89 (1 C, s), 35.49 (1 CH_3, s), 33.91 (1 CH_3, s), 7.84 (1 CH_3, s), 6.29 (1 CH_3, s); IR (neat) ν_{max}/cm^{-1} 1690, 1279, 1226, 1167, 1118, 1060, 1013; HRMS (ESI) obs'd 391.1010 ($MH^+ = C_{13}H_{14}F_7N_4O_2^+$) calc'd 391.1005.

III. Synthesis of compound **3**

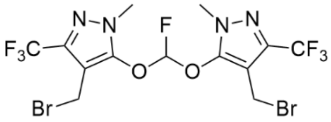

5,5'-((fluoromethylene)bis(oxy))bis(4-(bromomethyl)-1-methyl-3-(trifluoromethyl)-1*H*-pyrazole)

Compound **1** (4.98 g, 13 mmol, 1.0 equiv) was taken up in dry 1,2-dichloroethane (120 g) and sparging with nitrogen commenced. Dibromohydantoin (8.0 g, 28 mmol, 2.2 equiv) then benzoyl peroxide (0.82 g, 0.20 equiv) were added. After 10 minutes of sparging with nitrogen the reaction was heated to reflux (4 h). The reaction was cooled to ambient temperature and quenched with 5% aqueous sodium thiosulfate (200 mL). Hexane (100 mL) was added and the organic phase washed with water (200 mL), brine (100 mL) and then passed through a 5 cm silica plug, washing with 50% ethyl acetate/ 50% hexane (100 mL). Volatiles were removed in vacuo and the crude compound **2** (9.45 g; 65% chromatographic purity by HPLC, integration at 230 nm; 88%) used directly for the next reaction. An analytical sample was obtained by flash column chromatography (ethyl acetate 10%, hexanes 90%); colorless oil; ^{1}H NMR (400 MHz, $CDCl_3$) 6.92 (1 H, d, $^{2}J_{H\text{-}F}$ 73), 4.41 (4 H, s), 3.82 (6 H, s); ^{19}F NMR (376 MHz, $CDCl_3$) -62.75 (6 F, s), -81.73 (1 F, d, $^{2}J_{H\text{-}F}$ 73); ^{13}C NMR (101 MHz, $CDCl_3$) 144.13 (2 C, s), 138.93 (2 C, q, $^{2}J_{C\text{-}F}$ 38), 120.69 (2 CF_3, q, $^{1}J_{C\text{-}F}$ 270), 114.09 (1 CHF, d, $^{1}J_{C\text{-}F}$ 266), 105.94 (2 C, s), 36.03 (2 CH_3, s), 17.76 (2 CH_2, s); IR (neat) ν_{max}/cm^{-1} 1737, 1501, 1305, 1204, 1177,1129, 1074, 949, 931, 817; HRMS (ESI) obs'd 546.9215 ($MH^+ = C_{13}H_{12}{}^{79}Br_2F_7N_4O_2{}^+$) calc'd 546.9215.

IV. Synthesis of compound **2**

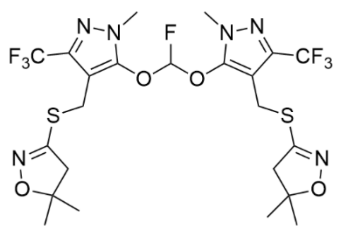

3,3'-(((((fluoromethylene)bis(oxy))bis(1-methyl-3-(trifluoromethyl)-1*H*-pyrazole-5,4-diyl))bis(methylene))bis(sulfanediyl))bis(5,5-dimethyl-4,5-dihydroisoxazole)

Compound **2** (9.45 g, 65% purity, 11 mmol) was taken up in MeCN (100 mL) and the solution heated to 50 °C. A solution of potassium carbonate (11 g, 80 mmol, 7.1 equiv) in water (50 mL) was added. Over 30 minutes, a solution of 5,5-dimethyl-3-(4*H*-isoxazolyl) carbamimidothioate hydrochloride (7.4 g, 35 mmol, 3.1 equiv) in water (50 mL) was added. Stirring was continued at 50 °C for 1 hour. The acetonitrile layer was concentrated in vacuo. The aqueous layer was extracted with ethyl acetate (2 × 50 mL) and the combined organic extracts added to the concentrate of the acetonitrile partition of the reaction mixture and the solution obtained washed with saturated aqueous sodium hydrogencarbonate (100 mL), brine (100 mL) and concentrated in vacuo. Column chromatography (ethyl acetate 20%, hexane 80%) gave compound **2** as a light-yellow solid (5.3 g, 73%); ^{1}H NMR (500 MHz, $CDCl_3$) 7.02 (1 H, d, $^{2}J_{H\text{-}F}$ 75), 4.23 (2 H, d, $^{2}J_{H\text{-}H}$ 14), 4.16 (2 H, d, $^{2}J_{H\text{-}H}$ 14), 3.80 (6 H, s), 2.75 (4 H, s), 1.38 (12 H, s); ^{19}F NMR (376 MHz, $CDCl_3$) -62.25 (6 F, s), -82.27 (1 F, d, $^{2}J_{H\text{-}F}$ 75); ^{13}C NMR (126 MHz, $CDCl_3$) 154.11 (2 C, s), 144.36 (2 C, s), 139.04 (2 C, q, $^{2}J_{C\text{-}F}$ 38), 120.94 (2 CF_3, q, $^{1}J_{C\text{-}F}$ 270), 115.50 (1 CHF, d, $^{1}J_{C\text{-}F}$ 265), 103.60 (2 C, s), 84.95 (2 C, s), 50.08 (2 CH_2), 35.99 (2 CH_3), 26.77 (4 C, CH_3), 22.20 (2 CH_2, s); IR (neat) ν_{max}/cm^{-1} 1370, 1285, 1171, 1124, 1066, 1023, 888, 791; HRMS (ESI) obs'd 649.151 ($MH^+ = C_{23}H_{28}F_7N_6O_4S_2^+$) calc'd 649.150.

V. Synthesis of compound **1**

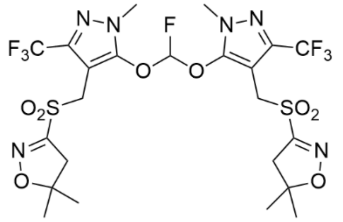

3,3'-((((fluoromethylene)bis(oxy))bis(1-methyl-3-(trifluoromethyl)-1*H*-pyrazole-5,4-diyl))bis(methylenesulfonyl))bis(5,5-dimethyl-4,5-dihydroisoxazole)

Compound **2** (1.0 g, 1.5 mmol, 1.0 equiv.) was taken up in MeOH (20 mL) and a solution of sodium tungstate dihydrate (100 mg, 0.30 mmol, 0.2 equiv) in water (1 mL) added. The mixture was heated to 45 °C and hydrogen peroxide (30%, 3.2 mL, 20 equiv) added in 3 portions at 3 hour intervals. After stirring at 45 °C for a further 24 hours the solution was cooled to ambient temperature, diluted with water (200 mL) and extracted with ethyl acetate (3 × 30 mL). The combined ethyl acetate extracts were washed with 5% aqueous sodium thiosulfate (100 mL), brine (100 mL) and concentrated in vacuo. Column chromatography (ethyl acetate 30%, hexane 70%, CH_2Cl_2 10%) gave compound **1** as a colorless glass (0.73 g, 66%). A powder may be obtained by recrystallization from methanol/water; mp 82-86 °C; ^{1}H NMR (500 MHz, $CDCl_3$) 7.04 (1 H, d, $^2J_{H\text{-}F}$ 76), 4.58 (2 H, d, $^2J_{H\text{-}H}$ 18.5), 4.55 (2 H, d, $^2J_{H\text{-}H}$ 18.5), 3.81 (6 H, s), 3.03 (2 H, d, $^2J_{H\text{-}H}$ 17.5), 2.98 (2 H, d, $^2J_{H\text{-}H}$ 17.5), 1.43 (6 H, s), 1.42 (6 H, s); ^{19}F NMR (376 MHz, $CDCl_3$) -61.71 (6 F, s), -82.10 (1 F, d, $^2J_{H\text{-}F}$ 76); ^{13}C NMR (126 MHz, $CDCl_3$) 157.13 (2 C, s), 145.07 (2 C, s), 139.37 (2 C, q, $^2J_{C\text{-}F}$ 38), 119.55 (2 CF_3, q, $^1J_{C\text{-}F}$ 270), 115.87 (1 CHF, d, $^1J_{C\text{-}F}$ 264), 92.49 (2 C, s), 89.41 (2 C, s), 46.80 (2 CH_2), 42.53 (2 CH_2), 35.42 (4 C, CH_3), 26.01 (2 CH_3, s), 25.96 (2 CH_3, s); ^{15}N NMR (71 MHz, $CDCl_3$) 11.1 (C=**N**-O), -82.1 (C=**N**-N), -182.3 (N-**N**(Me)-C); IR (neat) ν_{max}/cm^{-1} 2985, 1328, 1252, 1180, 1124, 1099, 1072, 945, 910, 724, 539; HRMS (ESI) obs'd 712.1220 ($M^+ = C_{23}H_{27}F_7N_6O_8S_2^+$) calc'd 712.1220.

VI. Unsuccessful reaction of **5** and **6**

Compound **6** (1.6 g) and Compound **5** (2.0 g) were taken up in dioxane (25 mL) and water (25 mL) and $Ca(OH)_2$ (5.0 g) added. The mixture was heated to reflux (72 h) then filtered, washing the filter cake with ethyl acetate (100 mL). The ethyl acetate wash was combined with the filtrate and water (100 mL) added. After shaking, the water layer was washed with ethyl acetate (50 mL) and the two ethyl acetate extracts combined and washed with 5% aqueous NaOH (100 mL), brine (50 mL) and dried over $MgSO_4$. Evaporation of the solvent gave a crude oil which was heated to 40 °C, with stirring, at 5 mbar pressure, to remove excess **5**. Flash column chromatography of the residue (ethyl acetate 20%, hexanes 80%) gave compound **1** (0.17 g, 5%).

VII. Unsuccessful reaction of **6** and ClF_2CO_2Na

Compound **6** (70.9 g, 0.39 mol, 2.0 equiv) was taken up in DMF (150 mL) and potassium carbonate (103 g, 0.75 mol, 3.8 equiv) added. Sodium chlorodifluoroacetate (30 g, 0.20 mol, 1 equiv) was added. The reaction was heated to 95 °C, with stirring (3 h). Approximately 120 mL of DMF was distilled from the reaction mixture in vacuo and 500 mL of water added to the residual paste. The resulting mixture was extracted with ethyl acetate (2 × 250 mL) wand the combined ethyl acetate extracts washed with water (500 mL), dried over $MgSO_4$ and concentrated. Flash column chromatography of the residue (ethyl acetate 20%, hexanes 80%) and collection of the fractions containing compound **4** followed by evaporation gave 19.9 g of an oil comprising ca. 19% of **4** by HPLC (ca. 5% yield).

VIII. Synthesis of compound **9**.

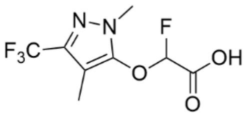

2-((1,4-dimethyl-3-(trifluoromethyl)-1*H*-pyrazol-5-yl)oxy)-2-fluoroacetic acid

To Cs_2CO_3 (20 g) in DMF (100 mL) was added ethyl α-bromo-α-fluoroacetate (4.0 mL) and compound **6** (6.0 g). The mixture was stirred at 35 °C (16 h) then filtered and *ca*. 50 mL of DMF removed in vacuo. Water (50 mL) was added and the resulting suspension stirred at 50 °C for 4 hours then washed with MTBE (2 × 50 mL). The remaining aqueous phase was acidified to pH 2 with 10 M HCl and extracted with EtOAc (2 × 50 mL), then the EtOAc extracts dried over $MgSO_4$ and the solvent removed in vacuo to give a residue which was recrystallized from methanol/water to give Compound **9** (6.9 g, 80%) as colorless crystals; mp 136-138 °C; ^{1}H NMR (500 MHz, DMSO-d_6) 6.22 (1 H, d, $^{2}J_{H\text{-}F}$ 57), 3.74 (3 H, s), 2.02 (3 H, s); ^{19}F NMR (376 MHz, $CDCl_3$) -62.95 (3 F, s), -129.04 (1 F, d, $^{2}J_{H\text{-}F}$ 57); ^{13}C NMR (126 MHz, DMSO-d_6) 164.38 (1 C, d, $^{2}J_{C\text{-}F}$ 30), 147.85 (1 C, s), 136.82 (1 C, q, $^{2}J_{C\text{-}F}$ 36), 121.50 (1 CF_3, q, $^{1}J_{C\text{-}F}$ 269), 104.65 (1 CHF, d, $^{1}J_{C\text{-}F}$ 237), 101.74 (1 C, s), 35.11 (1 CH_3, s), 5.96 (1 CH_3, s); IR (neat) ν_{max}/cm^{-1} ~2900 (br), 1776, 1586, 1128, 1070, 1043, 736, 701; HRMS (ESI) obs'd 256.0471 ($M^+ = C_8H_8F_4N_2O_3^+$) calc'd 256.0471.

*Prior to heating in a mixture of DMF and aqueous base some of the intermediate ester (compound **9** ethyl ester) may be observed, although ester cleavage occurs concurrently with the O-alkylation:*

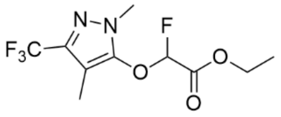

ethyl 2-((1,4-dimethyl-3-(trifluoromethyl)-1*H*-pyrazol-5-yl)oxy)-2-fluoroacetate

Compound **9** ethyl ester can be isolated from the MTBE washes during workup as a colorless liquid; ^{1}H NMR (400 MHz, $CDCl_3$) 5.78 (1 H, d, $^{2}J_{H\text{-}F}$ 57), 4.38 (2 H, q, $^{3}J_{H\text{-}H}$ 7.2), 3.79 (3 H, s), 2.09 (3 H, s), 1.38 (3 H, t, $^{3}J_{H\text{-}H}$ 7.2); ^{19}F NMR (376 MHz, $CDCl_3$) -63.33 (3 F, s), -129.57 (1 F, d, $^{2}J_{H\text{-}F}$ 57); ^{13}C NMR (151 MHz, $CDCl_3$) 163.10 (1 C, d, $^{2}J_{C\text{-}F}$ 31), 148.29 (1 C, s), 139.22 (1 C, q, $^{2}J_{C\text{-}F}$ 37), 121.79 (1 CF_3, q, $^{1}J_{C\text{-}F}$ 269), 104.92 (1 CHF, d, $^{1}J_{C\text{-}F}$ 240), 102.56 (1 C, s), 63.37 (1 CH_2, s), 35.44 (1 CH_3, s), 13.99 (1 CH_3, s), 6.29 (1 CH_3, s); IR (neat) ν_{max}/cm^{-1} 1765, 1484, 1384, 1278, 1167, 1120, 1059, 1016, 855, 734; HRMS (ESI) obs'd 284.0784 (M^+ = $C_{10}H_{12}F_4N_2O_3^+$) calc'd 284.0784.

IX. General procedure for the synthesis of bis(aryloxy)fluoromethanes.

To potassium hydroxide (4 mmol), in a 21 mL vial, at ambient temperature, was added acetonitrile (5 mL), followed by the phenol (1.3 mmol). Dibromofluoromethane (1.3 mmol) was added and the vial capped. After stirring (16 h) the vial was uncapped and volatile components removed in vacuo. Ethyl acetate (5 mL) and water (10 mL) were added. After stirring for one minute the ethyl acetate layer was added to silica (2 g, 0.015–0.04 µm) and ethyl acetate removed in vacuo. Automated column chromatography using gradient elution (0→40% EtOAc in hexanes) gave, after evaporation, the product. Unreacted phenol may be recovered by acidification of the water layer and filtration or extraction.

X. Compound **11**

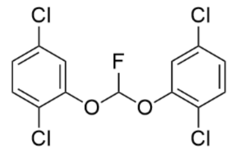

2,2'-((fluoromethylene)bis(oxy))bis(1,4-dichlorobenzene)

Compound **11** was produced according to the general procedure; colorless powder (0.25 g, 54% or 68% based on recovered 2,5-dichlorophenol); mp 214-215 °C; ^{1}H NMR (400 MHz, DMSO-d_6) 7.63-7.64 (2 H, m), 7.62 (2 H, d, $^{3}J_{H-H}$ 8.8), 7.56 (1 H, d, $^{2}J_{H-F}$ 74), 7.33 (2 H, dd, $^{3}J_{H-H}$ 8.8, $^{4}J_{H-H}$ 2.4); ^{19}F NMR (376 MHz, DMSO-d_6) -87.12 (1 F, d, $^{2}J_{H-F}$ 74); ^{13}C NMR (101 MHz, DMSO-d_6) 148.26 (2 C, s), 132.25 (2 C, s), 131.62 (2 CH, s), 125.61 (2 CH, s), 122.50 (2 CH, s), 119.37 (2 C, s), 113.84 (1 CHF, d, $^{1}J_{C-F}$ 252); IR (neat) ν_{max}/cm^{-1} 1580, 1469, 1343, 1222, 1095, 842, 805, 574; HRMS (APCI) obs'd 355.915 ($M^{+} = C_{13}H_{7}{}^{35}Cl_{3}{}^{37}ClFO_{2}^{-}$) calc'd 355.915.

XI. Compound **12**

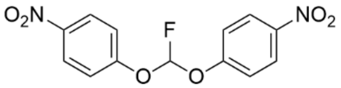

4,4'-((fluoromethylene)bis(oxy))bis(nitrobenzene)

Compound **12** was produced according to the general procedure; colorless crystals (0.21 g, 52% or 64% based on recovered 4-nitrophenol); mp 111 °C; ^{1}H NMR (400 MHz, $CDCl_3$) 8.27 (2 H, d, $^{3}J_{H-H}$ 8.8), 7.28 (2 H, d, $^{3}J_{H-H}$ 8.8), 6.85 (1 H, d, $^{2}J_{H-F}$ 72); ^{19}F NMR (376 MHz, $CDCl_3$) -85.76 (1 F, d, $^{2}J_{H-F}$ 72); ^{13}C NMR (101 MHz, $CDCl_3$) 156.92 (2 C, s), 144.42 (2 C, s), 131.62 (4 CH, s), 125.901 (4 CH, s), 118.49 (2 CH, s), 113.024 (1 CHF, d, $^{1}J_{C-F}$ 253); IR (neat) ν_{max}/cm^{-1} 1515, 1344, 1207, 1123, 1108, 967, 860, 846; HRMS (APCI) obs'd 353.043 ($M.HCOO^{-} = C_{14}H_{10}FN_{2}O_{8}^{-}$) calc'd 353.042.

XII. Synthesis of compound **4** by the general procedure

To potassium hydroxide (224 mg, 4.0 mmol, 3.1 equiv), in a 21 mL vial, at ambient temperature, was added acetonitrile (5 mL), followed by the compound **6** (234 mg, 1.3 mmol, 1.0 equiv). Dibromofluoromethane (103 µL, 1.3 mmol, 1.0 equiv) was added and the vial capped. After stirring (16 h) the vial was uncapped and volatile components removed in vacuo. Ethyl acetate (5 mL) and water (10 mL) were added. The ethyl acetate fraction was analyzed by HPLC, and found to contain an approximately 3:2:0.05 ratio of compounds **4**:**7**:**8** by HPLC integration at 230 nm.
