# Supplementary material for: Synthesis of bis(aryloxy)fluoromethanes using a heterodihalocarbene strategy
Source: Beilstein J Org Chem. 2021 Apr 12;17:813–8. doi: 10.3762/bjoc.17.70 (PMC8056069; doi:10.3762/bjoc.17.70)
Supplement: File 2 — Copies of NMR spectra. [file Beilstein_J_Org_Chem-17-813-s002.pdf]

BEILSTEIN JOURNAL OF ORGANIC CHEMISTRY

Supporting Information

for

Synthesis of bis(aryloxy)fluoromethanes using a heterodihalocarbene strategy

Carl Recsei and Yaniv Barda

Beilstein J. Org. Chem. **2021,** *17,* 813–818. doi:10.3762/bjoc.17.70

Copies of NMR spectra

License and Terms: This is a supporting information file under the terms of the Creative Commons Attribution License (https://creativecommons.org/licenses/by/4.0). Please note that the reuse, redistribution and reproduction in particular requires that the author(s) and source are credited and that individual graphics may be subject to special legal provisions.
The license is subject to the *Beilstein Journal of Organic Chemistry* terms and conditions: (https://www.beilstein-journals.org/bjoc/terms)

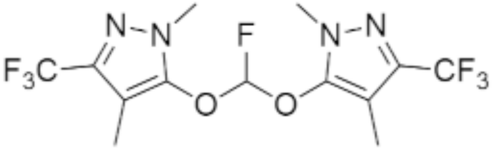

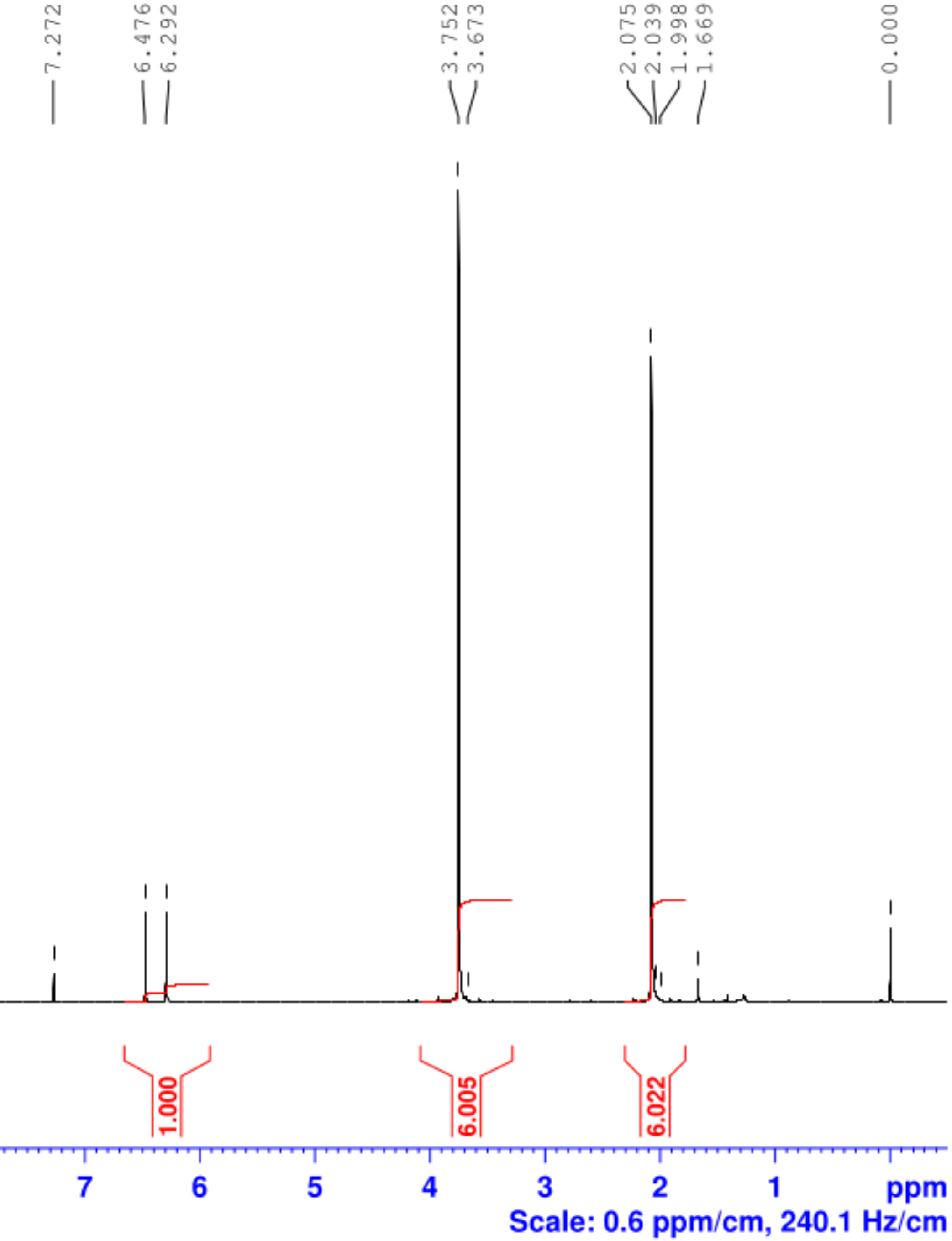

NAME	P7K8.12.19
EXPNO	1
PROCNO	1
Date_	20191208
Time	13.13
INSTRUM	spect
PROBHD	5 mm PABBO BB-
PULPROG	zg30
TD	65536
SOLVENT	CDCl3
NS	24
DS	0
SWH	7183.908 Hz
FIDRES	0.109618 Hz
AQ	4.5613556 sec
RG	128
DW	69.600 use
DE	6.50 use
TE	300.0 K
D1	0.10000000 sec
TD0	1

======== CHANNEL f1 ========

NUC1	1H
P1	10.10 use
PL1	0.00 dB
SFO1	400.1032008 MHz
SI	32768
SF	400.1000058 MHz
WDW	EM
SSB	0
LB	0.00 Hz
GB	0
PC	1.00

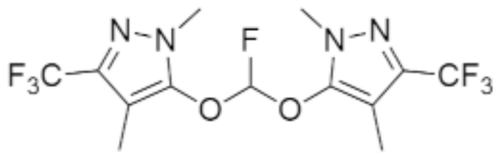

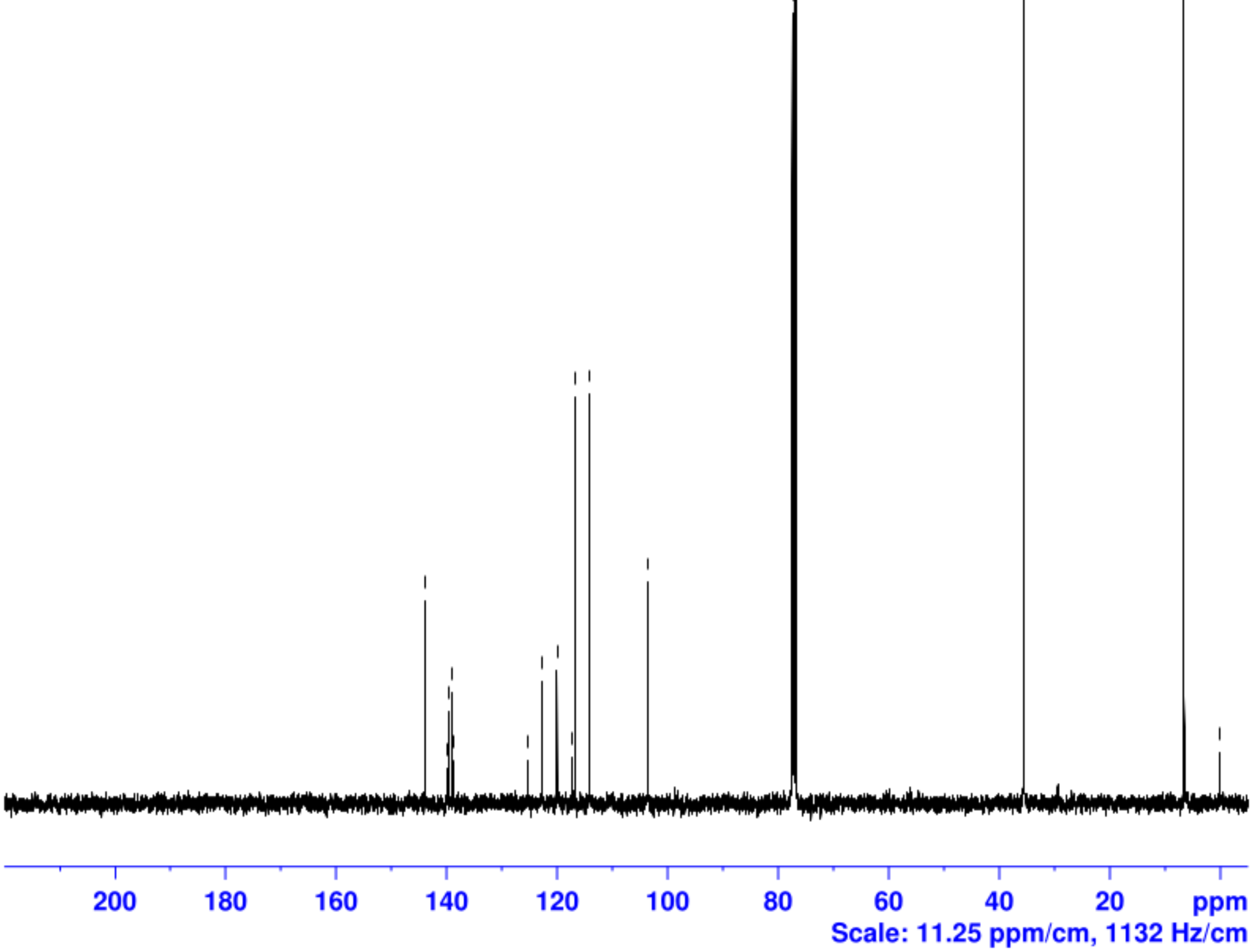

NAME	P7K8.12.19
EXPNO	6
PROCNO	1
Date_	20191209
Time	10.45
INSTRUM	spect
PROBHD	5 mm PABBO BB-
PULPROG	zgdc30
TD	32768
SOLVENT	CDCl3
NS	6076
DS	0
SWH	24154.590 Hz
FIDRES	0.737140 Hz
AQ	0.6783476 se
RG	71.8
DW	20.700 us
DE	29.57 us
TE	301.1 K
D1	0.10000000 se
D11	0.03000000 se
TD0	1
======== CHANNEL f1 ======	
NUC1	13C
P1	9.20 us
PL1	3.00 dB
SFO1	100.6152865 MH
======== CHANNEL f2 ======	
CPDPRG2	waltz16
NUC2	1H
PCPD2	80.00 us
PL2	0.00 dB
PL12	18.00 dB
SFO2	400.1009202 MH
SI	32768
SF	100.6052174 MH
WDW	EM
SSB	0
LB	2.00 Hz
GB	0
PC	1.00

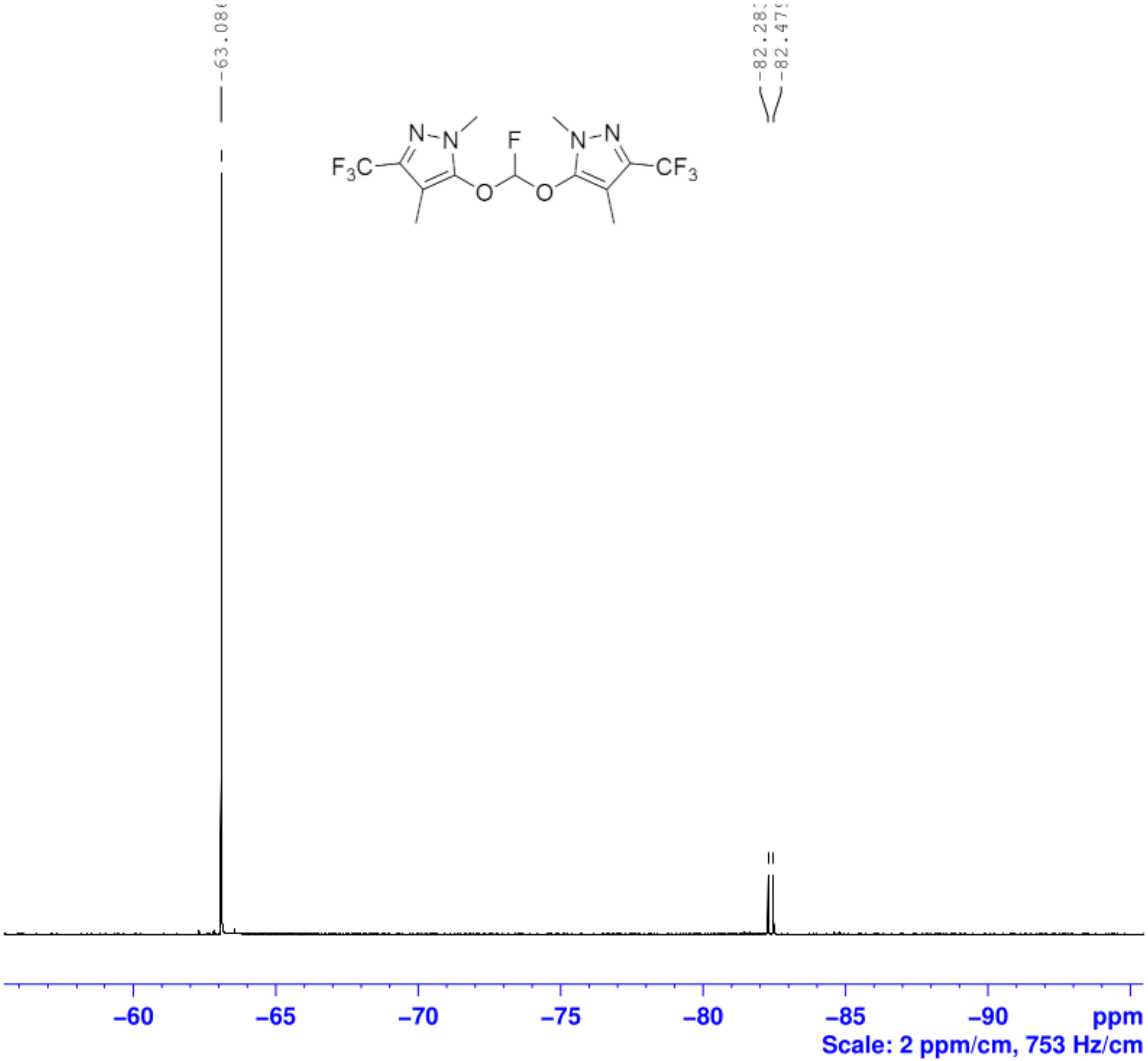

BRUKER

NAME	P7K8.12.19	
EXPNO	5	
PROCNO	1	
Date_	20191208	
Time	13.59	
INSTRUM	spect	
PROBHD	5 mm PABBO BB-	
PULPROG	zg30	
TD	32768	
SOLVENT	CDCl3	
NS	8	
DS	0	
SWH	15060.241	Hz
FIDRES	0.459602	Hz
AQ	1.0879476	se
RG	1149.4	
DW	33.200	us
DE	6.32	us
TE	300.0	K
D1	0.10000000	se
TD0	1	

======== CHANNEL f1 ======

NUC1	19F	
P1	7.90	us
PL1	0.00	dB
SFO1	376.4419017	MH
SI	32768	
SF	376.4703092	MH
WDW	EM	
SSB	0	
LB	0.50	Hz
GB	0	
PC	1.00	

HMBC

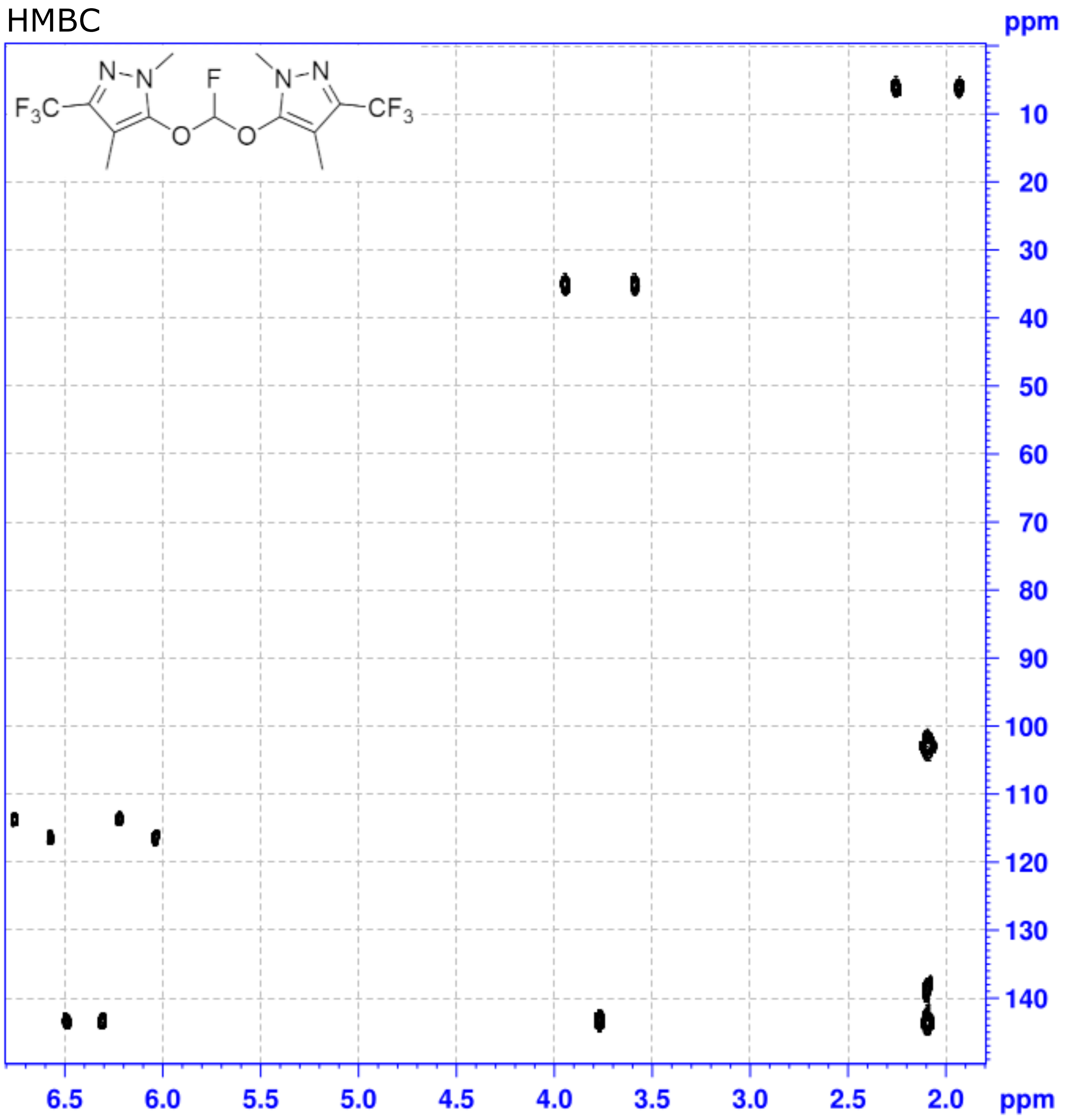

NAME	P7K8.12.19	
EXPNO	4	
PROCNO	1	
Date_	20191208	
Time	13.49	
INSTRUM	spect	
PROBHD	5 mm PABBO BB-	
PULPROG	hmbcgplpndqf	
TD	1024	
SOLVENT	CDCl3	
NS	4	
DS	2	
SWH	2003.205	Hz
FIDRES	1.956255	Hz
AQ	0.2556404	sec
RG	46341	
DW	249.600	usec
DE	241.25	usec
TE	300.1	K
CNST2	145.0000000	
CNST13	8.0000000	
D0	0.00000300	sec
D1	1.00000000	sec
D2	0.00344828	sec
D6	0.06250000	sec
D16	0.00020000	sec
IN0	0.00003315	sec
======== CHANNEL f1 ========		
NUC1	1H	
P1	10.10	usec
P2	20.20	usec
PL1	0.00	dB
SFO1	400.1017204	MHz
======== CHANNEL f2 ========		
NUC2	13C	
P3	9.20	usec
PL2	0.00	dB
SFO2	100.6127714	MHz
====== GRADIENT CHANNEL =====		
GPNAM1	sine.100	
GPNAM2	sine.100	
GPNAM3	sine.100	
GPZ1	50.00	%
GPZ2	30.00	%
GPZ3	40.10	%
P16	1000.00	usec
ND0	2	
TD	67	
SFO1	100.6128	MHz
FIDRES	225.252472	Hz
SW	150.000	ppm
FnMODE	QF	
SI	1024	
SF	400.1000005	MHz
WDW	QSINE	
SSB	0	
LB	0.00	Hz
GB	0	
PC	1.00	
SI	512	
MC2	QF	
SF	100.6052572	MHz
WDW	QSINE	
SSB	0	
LB	0.00	Hz
GB	0	

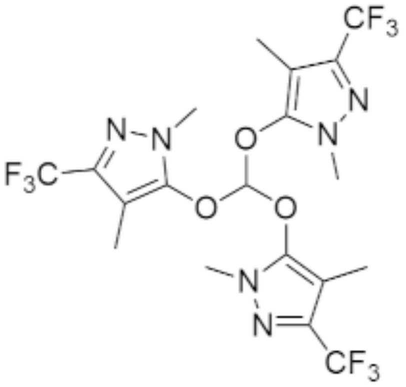

contains *ca.* 5% Compound **4**

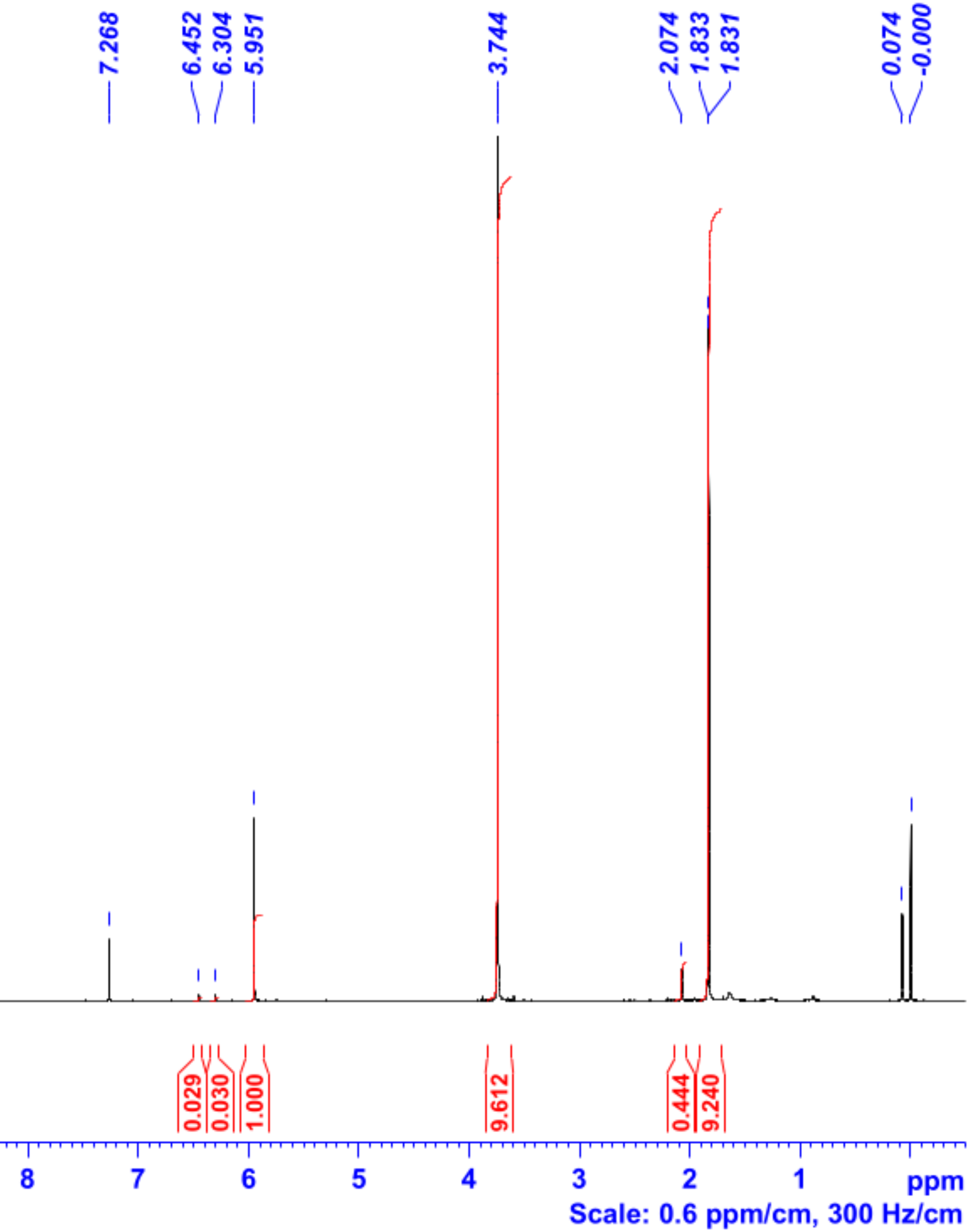


```
Current Data Parameters
NAME            P7K1.12.20
EXPNO                    1
PROCNO                   1

F2 - Acquisition Parameters
Date_             20201201
Time                10.26 h
INSTRUM              spect
PROBHD    Z8420_0082 (PH
PULPROG               zg30
TD                   65536
SOLVENT              CDCl3
NS                      16
DS                       0
SWH               7002.801 Hz
FIDRES            0.213709 Hz
AQ               4.6792703 sec
RG                     322
DW                  71.400 usec
DE                    6.50 usec
TE                   300.0 K
D1              0.10000000 sec
TD0                      1
SFO1           500.0430002 MHz
NUC1                    1H
P1                   10.32 usec
PLW1           15.00000000 W

F2 - Processing parameters
SI                   65536
SF             500.0400079 MHz
WDW                     EM
SSB       0
LB        0 Hz
GB        0
PC                    1.00
```

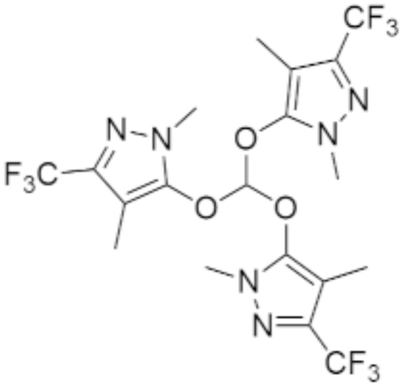

contains *ca*. 5% Compound **4**

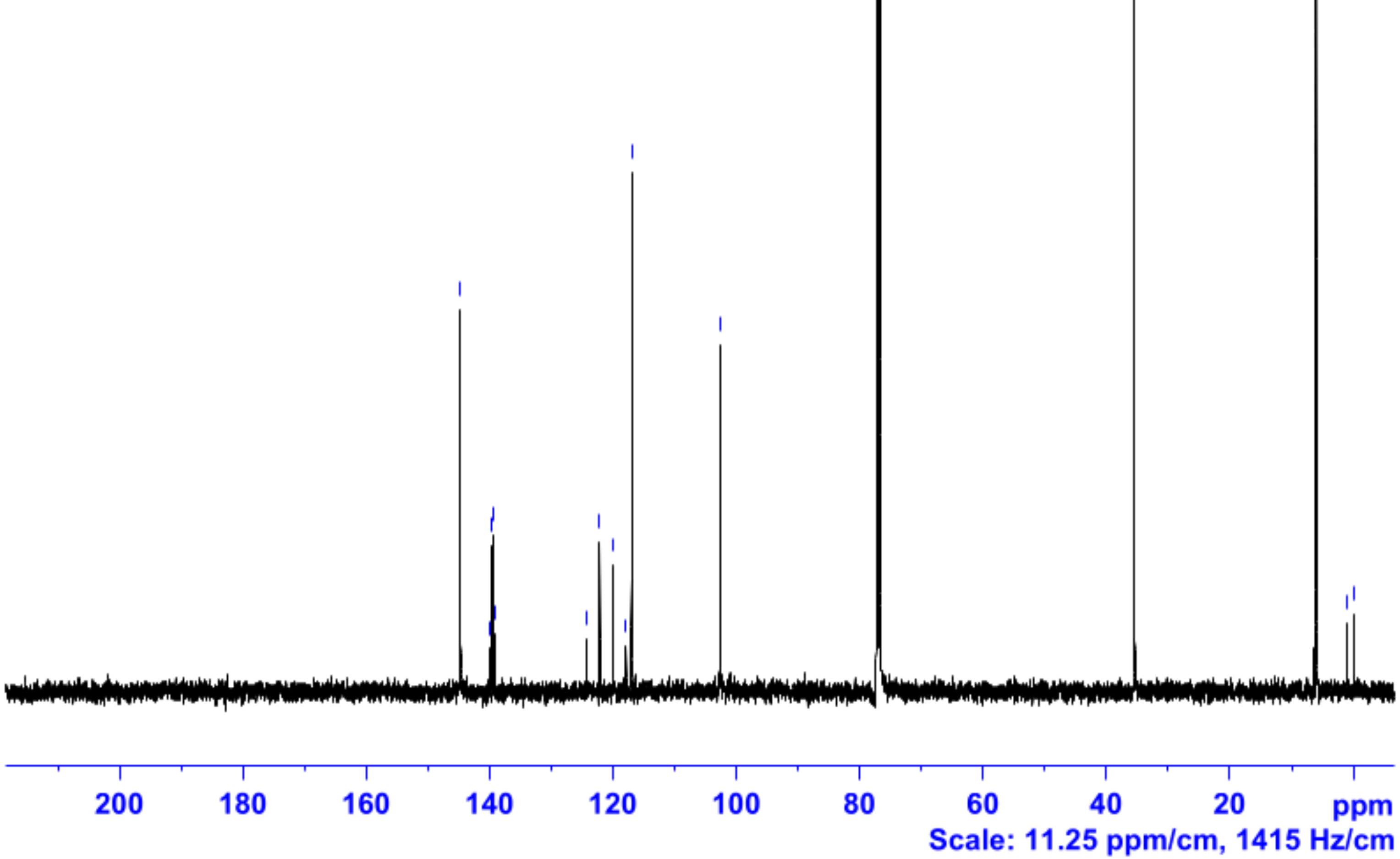

BRUKER

```
Current Data Parameters
NAME            P7K1.12.20
EXPNO                    2
PROCNO                   1

F2 - Acquisition Parameters
Date_             20201201
Time               10.49 h
INSTRUM              spect
PROBHD    Z8420_0082 (PH
PULPROG            zgdc30
TD                   65536
SOLVENT              CDCl3
NS                    1046
DS                       0
SWH          29761.904 Hz
FIDRES        0.908261 Hz
AQ           1.1010048 sec
RG                    2050
DW             16.800 usec
DE               6.50 usec
TE                300.0 K
D1         0.10000000 sec
D11        0.03000000 sec
TD0                      1
SFO1       125.7477315 MHz
NUC1                   13C
P1              13.00 usec
PLW1        55.00000000 W
SFO2       500.0425002 MHz
NUC2                    1H
CPDPRG[2           waltz16
PCPD2           80.00 usec
PLW2        15.00000000 W
PLW12        0.24962001 W

F2 - Processing parameters
SI                   32768
SF         125.7351531 MHz
WDW                     EM
SSB        0
LB                 2.00 Hz
GB         0
PC                    1.00
```

HMBC

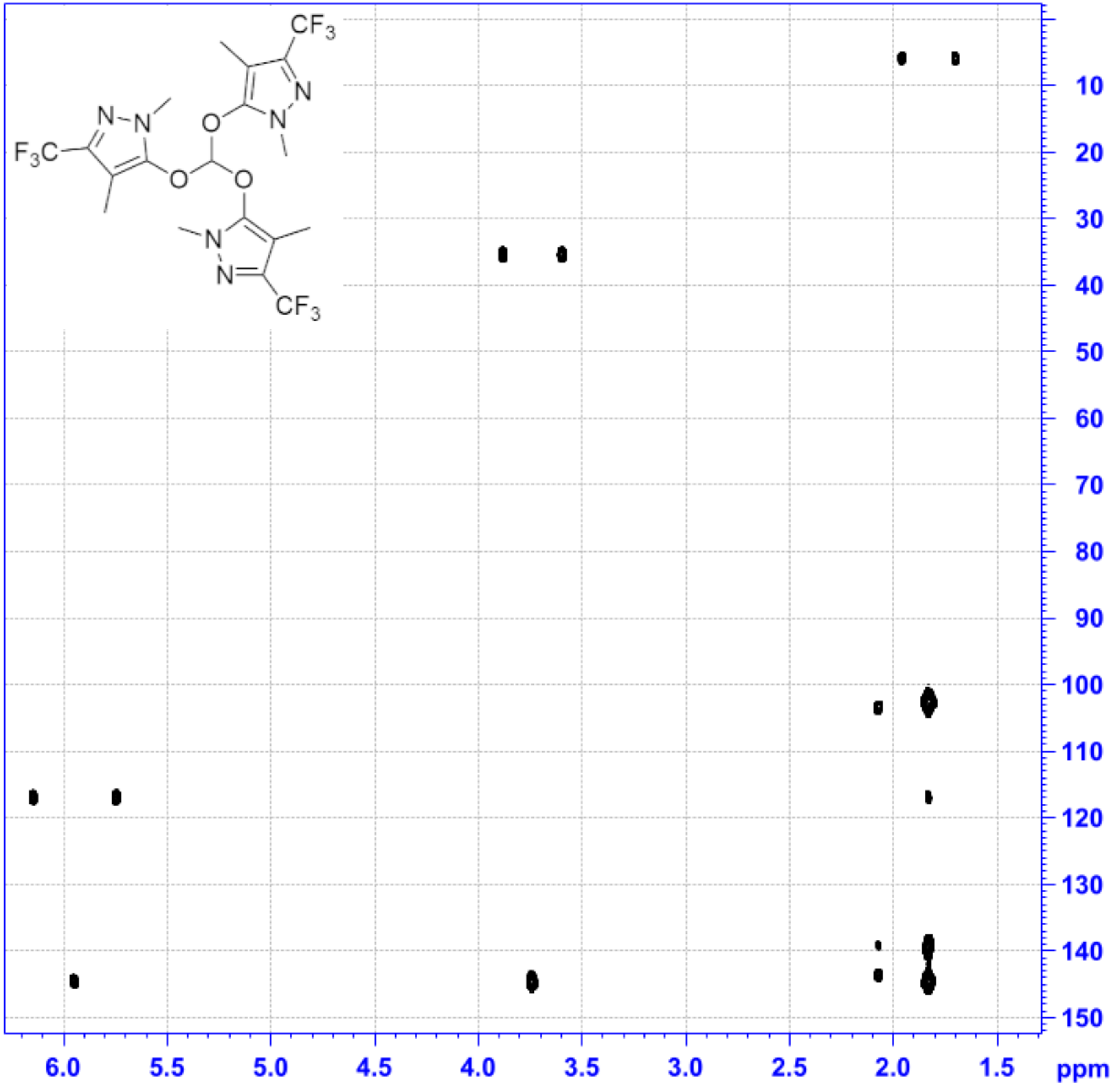


```
Current Data Parameters
NAME          P7K1.12.20
EXPNO                  5
PROCNO                 1

F2 - Acquisition Parameters
Date_           20201201
Time               11.21 h
INSTRUM            spect
PROBHD   Z8420_0082 (PH
PULPROG      hmbcgplpndqf
TD                  1024
SOLVENT            CDCl3
NS                     8
DS                     2
SWH             2500.000 Hz
FIDRES          4.882813 Hz
AQ             0.2048000 sec
RG                  2050
DW               200.000 usec
DE                  6.50 usec
TE                 300.0 K
CNST2        145.0000000
CNST13         8.0000000
D0            0.00000300 sec
D1            1.00000000 sec
D2            0.00344828 sec
D6            0.06250000 sec
D16           0.00020000 sec
IN0           0.00002570 sec
TDav                   1
SFO1         500.0419002 MHz
NUC1                  1H
P1                 10.32 usec
P2                 20.64 usec
PLW1         15.00000000 W
SFO2         125.7445881 MHz
NUC2                 13C
P3                 13.00 usec
PLW2         55.00000000 W
GPNAM[1]      SMSQ10.100
GPZ1               50.00 %
GPNAM[2]      SMSQ10.100
GPZ2               30.00 %
GPNAM[3]      SMSQ10.100
GPZ3               40.10 %
P16              1000.00 usec

F1 - Acquisition parameters
TD                   128
SFO1            125.7446 MHz
FIDRES        303.988312 Hz
SW               154.720 ppm
FnMODE                QF

F2 - Processing parameters
SI                  1024
SF           500.0400090 MHz
WDW                QSINE
SSB       0
LB        0 Hz
GB        0
PC                  1.00

F1 - Processing parameters
SI                   512
MC2                   QF
SF           125.7351530 MHz
WDW                QSINE
SSB       0
LB        0 Hz
GB        0
```

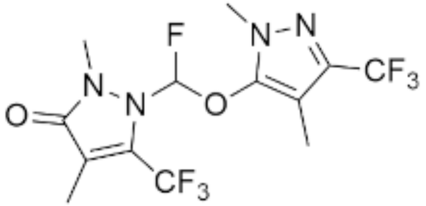

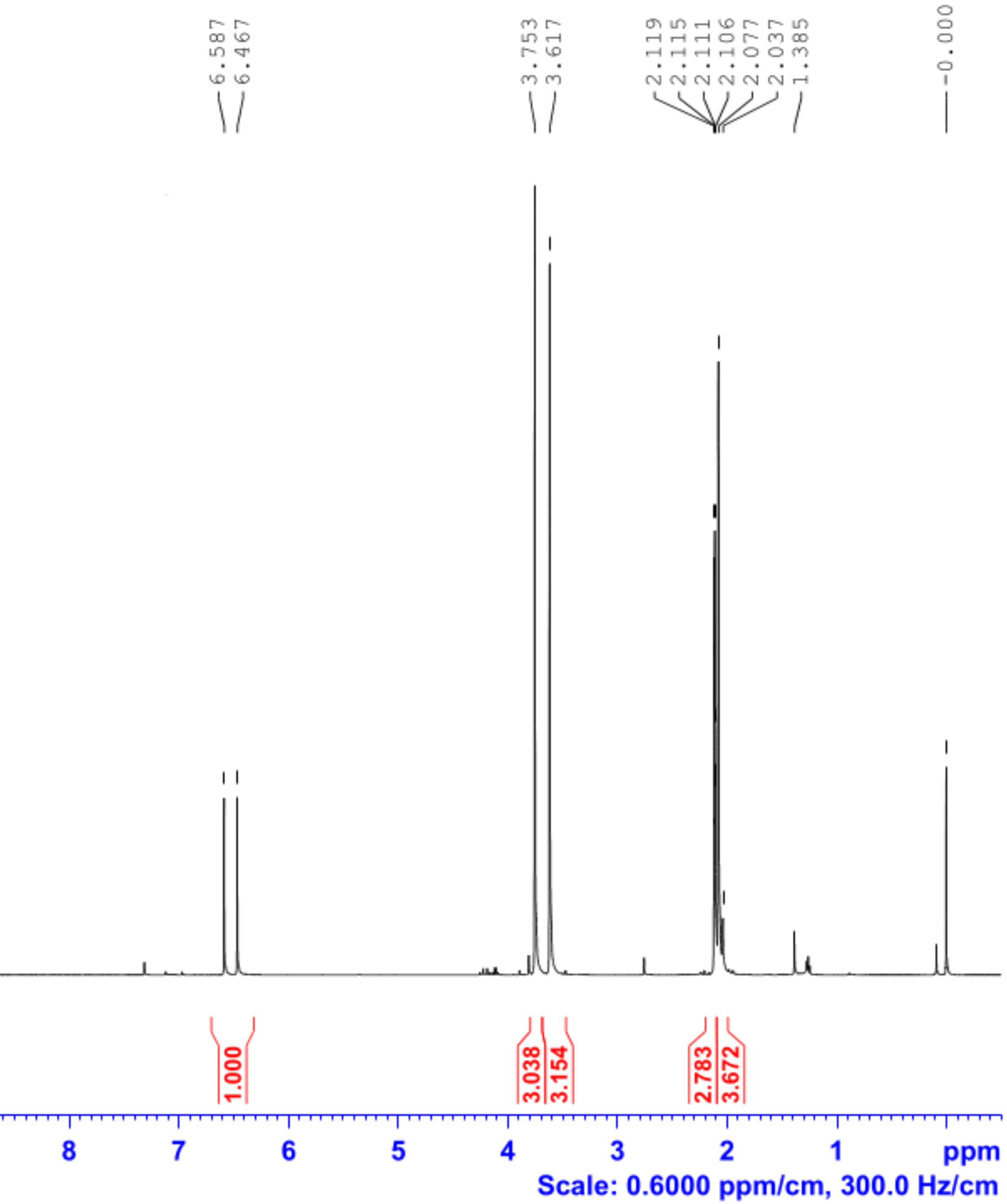

Current Data Parameters	
NAME	P7K10.2.21
EXPNO	1
PROCNO	1

F2 - Acquisition Parameters	
Date_	20210210
Time	14.31 h
INSTRUM	spect
PROBHD	Z8420_0082 (PH
PULPROG	zg30
TD	65536
SOLVENT	CDCl3
NS	24
DS	0
SWH	7002.801 Hz
FIDRES	0.213709 Hz
AQ	4.6792703 sec
RG	45.2
DW	71.400 usec
DE	6.50 usec
TE	300.0 K
D1	0.10000000 sec
TD0	1
SFO1	500.0430002 MHz
NUC1	1H
P1	10.32 usec
PLW1	15.00000000 W

F2 - Processing parameters	
SI	65536
SF	500.0399851 MHz
WDW	EM
SSB	0
LB	0 Hz
GB	0
PC	1.00

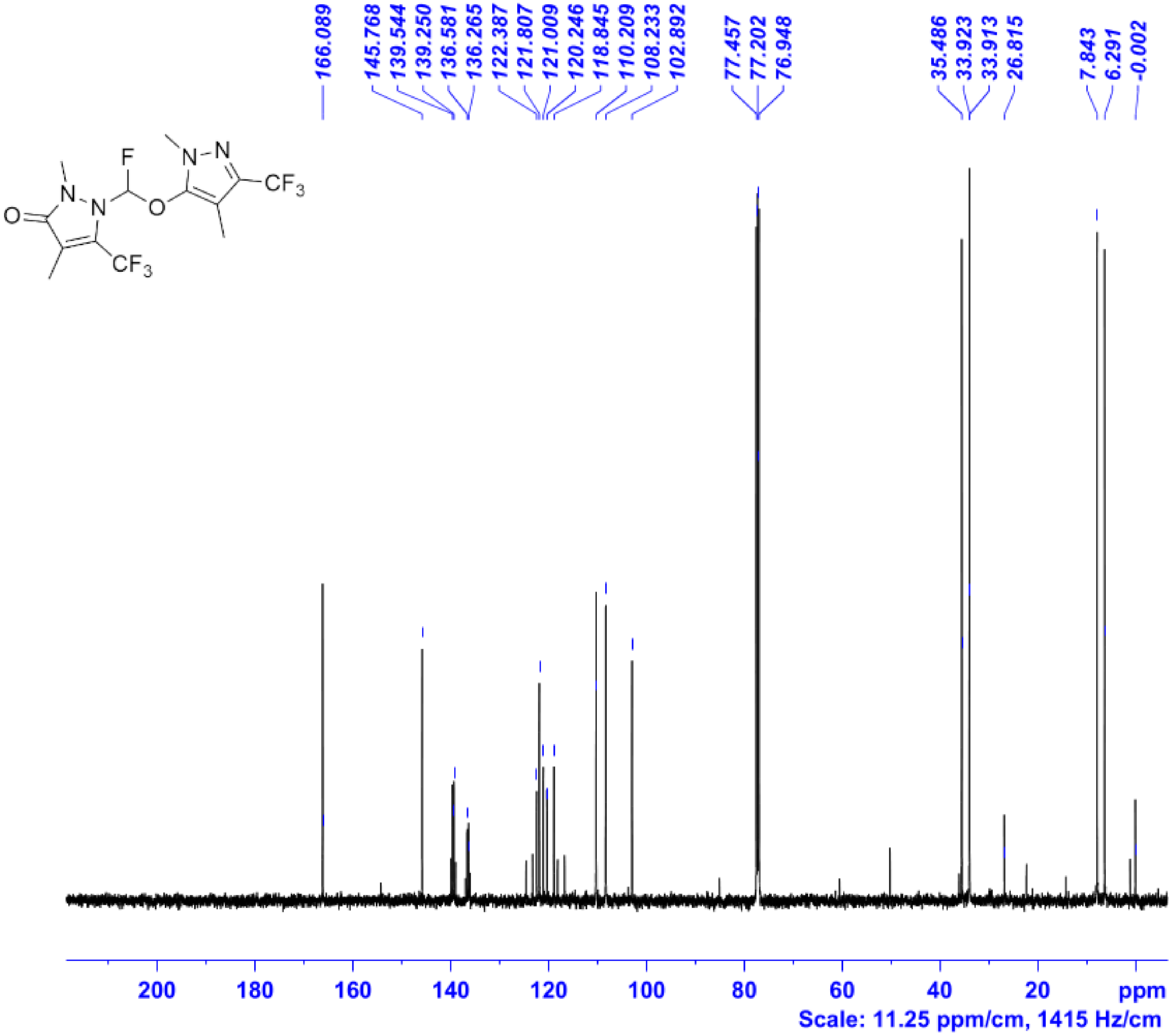

Current Data Parameters	
NAME	P7K10.2.21
EXPNO	2
PROCNO	1

F2 - Acquisition Parameters	
Date_	20210210
Time	15.51 h
INSTRUM	spect
PROBHD	Z8420_0082 (PH
PULPROG	zgdc30
TD	65536
SOLVENT	CDCl3
NS	900
DS	0
SWH	29761.904 Hz
FIDRES	0.908261 Hz
AQ	1.1010048 sec
RG	2050
DW	16.800 usec
DE	6.50 usec
TE	300.0 K
D1	0.10000000 sec
D11	0.03000000 sec
TD0	1
SFO1	125.7477315 MHz
NUC1	13C
P1	13.00 usec
PLW1	55.00000000 W
SFO2	500.0425002 MHz
NUC2	1H
CPDPRG[2	waltz16
PCPD2	80.00 usec
PLW2	15.00000000 W
PLW12	0.24962001 W

F2 - Processing parameters	
SI	32768
SF	125.7351385 MHz
WDW	EM
SSB	0
LB	2.00 Hz
GB	0
PC	1.00

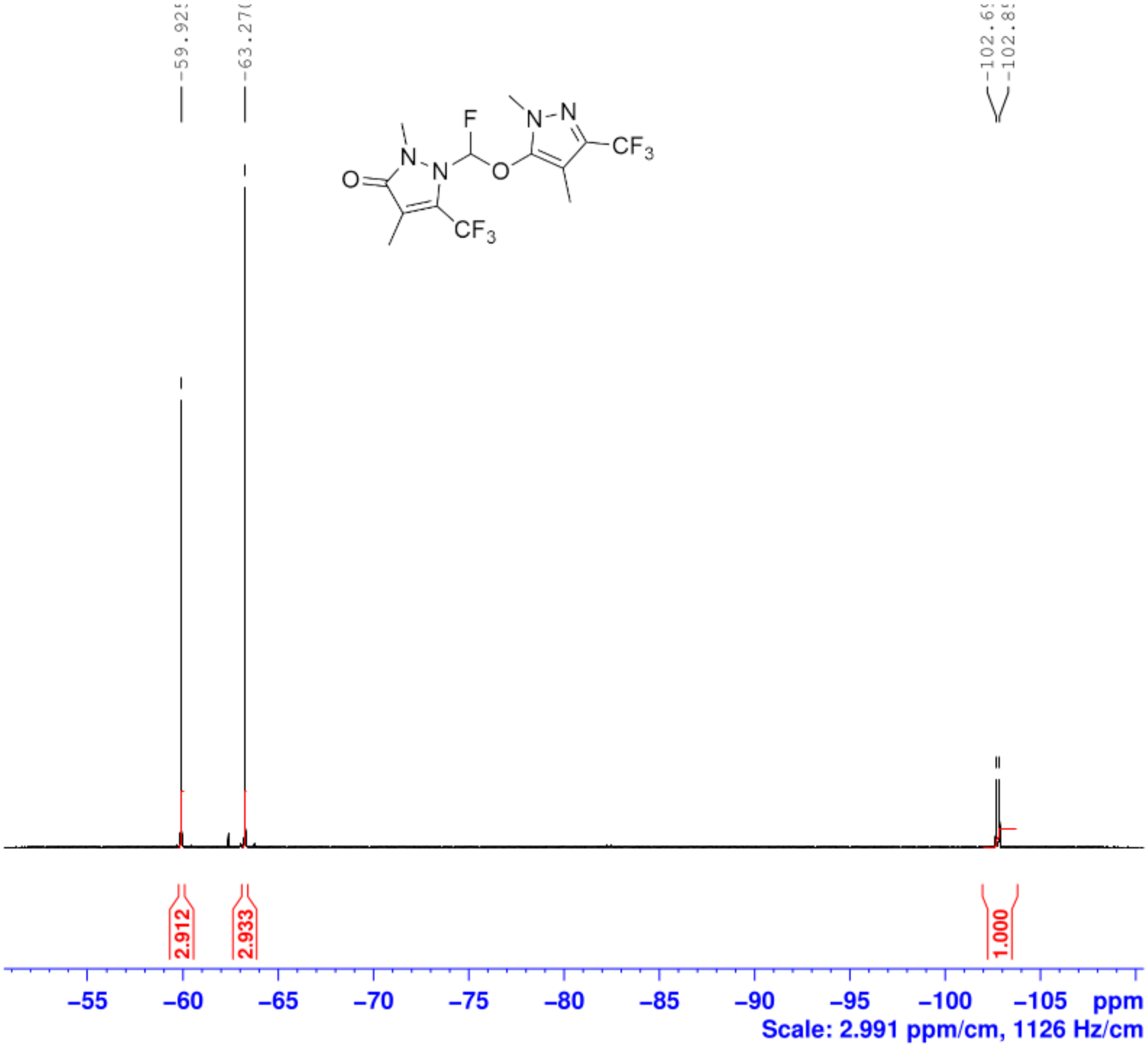

NAME	P7K10.2.21
EXPNO	3
PROCNO	1
Date_	20210214
Time	11.24
INSTRUM	spect
PROBHD	5 mm PABBO BB-
PULPROG	zg30
TD	32768
SOLVENT	CDCl3
NS	48
DS	0
SWH	22522.523 Hz
FIDRES	0.687333 Hz
AQ	0.7274996 se
RG	1625.5
DW	22.200 us
DE	6.32 us
TE	300.1 K
D1	0.10000000 se
TD0	1

======== CHANNEL f1 ======

NUC1	19F
P1	7.90 us
PL1	0.00 dE
SFO1	376.4400194 MH
SI	32768
SF	376.4703301 MH
WDW	EM
SSB	0
LB	0.50 Hz
GB	0
PC	1.00

HMBC

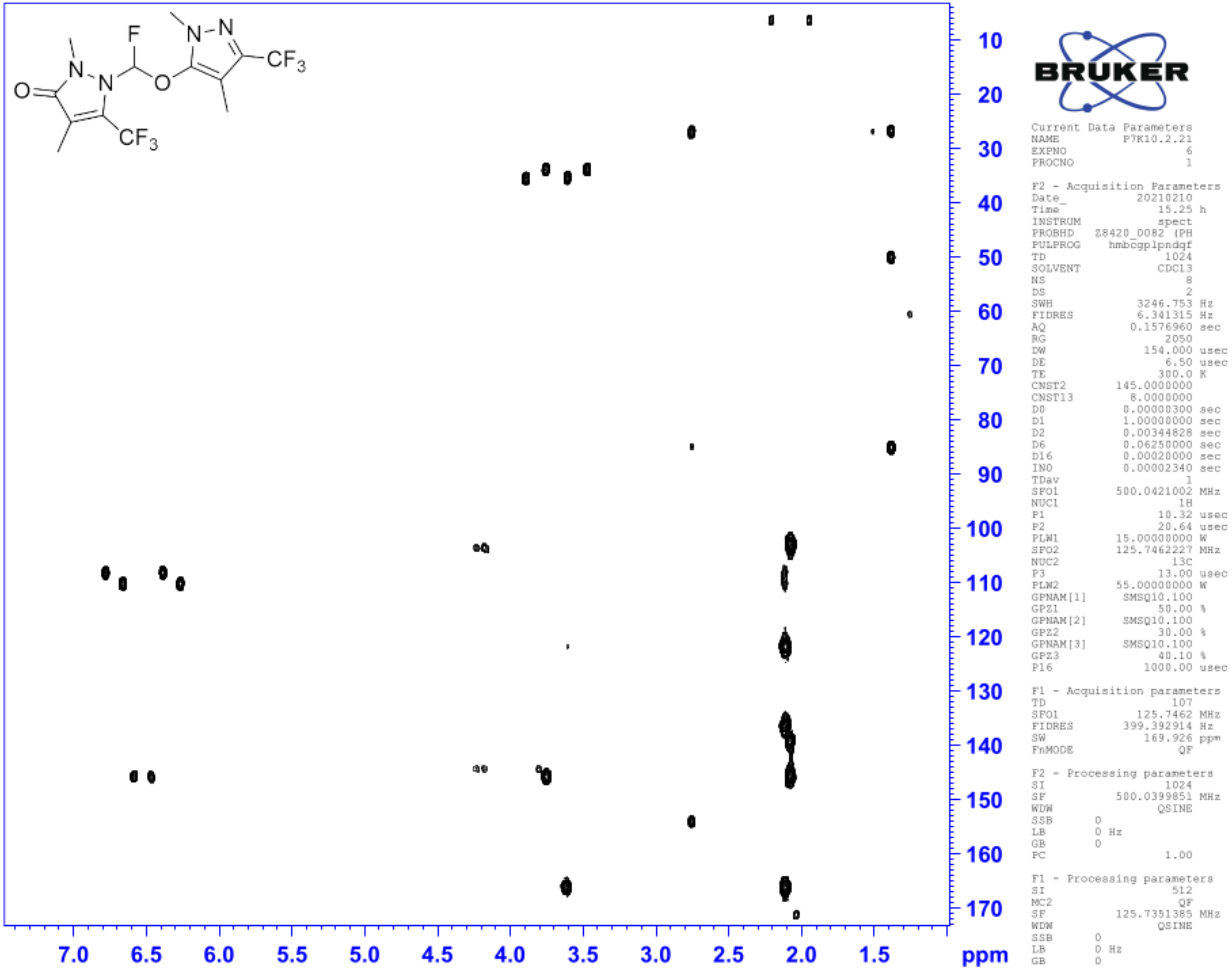
F
N
N
O
N
N
CF3
O
O
CF3
BRUKER
Current Data Parameters
NAME P7K10.2.21
EXPNO 6
PROCNO 1
F2 - Acquisition Parameters
Date_ 20210210
Time 15.25 h
INSTRUM spect
PROBHD Z8420_0082 (PH
PULPROG hmbcgplpndqf
TD 1024
SOLVENT CDCl3
NS 8
DS 2
SWH 3246.753 Hz
FIDRES 6.341315 Hz
AQ 0.1576960 sec
RG 2050
DW 154.000 usec
DE 6.50 usec
TE 300.0 K
CNST2 145.0000000
CNST13 8.0000000
D0 0.00000300 sec
D1 1.00000000 sec
D2 0.00344828 sec
D6 0.06250000 sec
D16 0.00020000 sec
IN0 0.00002340 sec
TDav 1
SFO1 500.0421002 MHz
NUC1 1H
P1 10.32 usec
P2 20.64 usec
PLW1 15.00000000 W
SFO2 125.7462227 MHz
NUC2 13C
P3 13.00 usec
PLW2 55.00000000 W
GPNAM[1] SMSQ10.100
GPZ1 50.00 %
GPNAM[2] SMSQ10.100
GPZ2 30.00 %
GPNAM[3] SMSQ10.100
GPZ3 40.10 %
P16 1000.00 usec
F1 - Acquisition parameters
TD 107
SFO1 125.7462 MHz
FIDRES 399.392914 Hz
SW 169.926 ppm
FnMODE QF
F2 - Processing parameters
SI 1024
SF 500.0399851 MHz
WDW QSINE
SSB 0
LB 0 Hz
GB 0
PC 1.00
F1 - Processing parameters
SI 512
MC2 QF
SF 125.7351385 MHz
WDW QSINE
SSB 0
LB 0 Hz
GB 0
ppm
10
20
30
40
50
60
70
80
90
100
110
120
130
140
150
160
170
7.0
6.5
6.0
5.5
5.0
4.5
4.0
3.5
3.0
2.5
2.0
1.5
ppm

HMQC

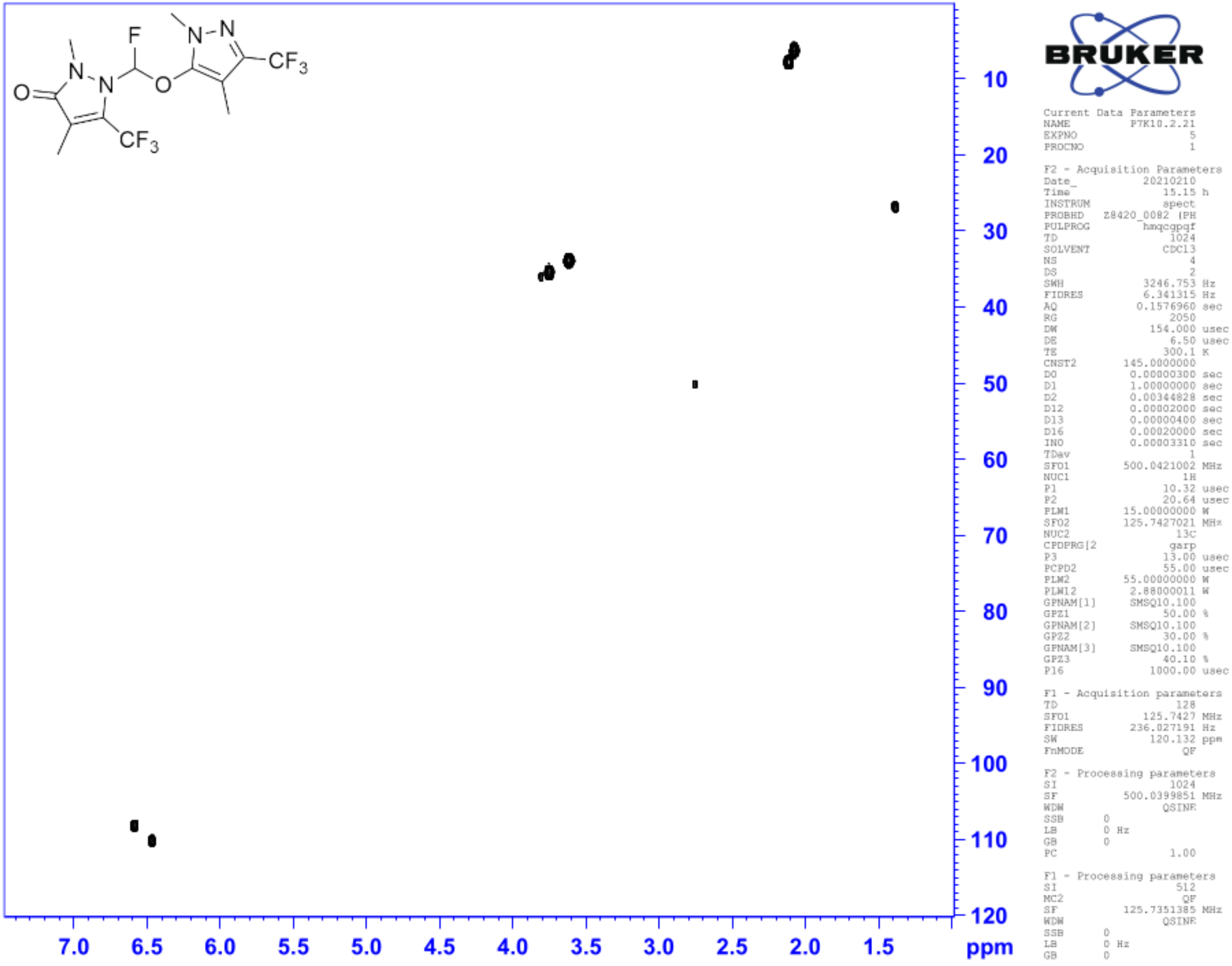

F
N
N
O
N
N
O
CF$_3$
CF$_3$
ppm
10
20
30
40
50
60
70
80
90
100
110
120
7.0
6.5
6.0
5.5
5.0
4.5
4.0
3.5
3.0
2.5
2.0
1.5
ppm
Current Data Parameters
NAME P7K10.2.21
EXPNO 5
PROCNO 1
F2 - Acquisition Parameters
Date_ 20210210
Time 15.15 h
INSTRUM spect
PROBHD Z8420_0082 (PH
PULPROG hmqcgpqf
TD 1024
SOLVENT CDCl3
NS 4
DS 2
SWH 3246.753 Hz
FIDRES 6.341315 Hz
AQ 0.1576960 sec
RG 2050
DW 154.000 usec
DE 6.50 usec
TE 300.1 K
CNST2 145.0000000
D0 0.00000300 sec
D1 1.00000000 sec
D2 0.00344828 sec
D12 0.00002000 sec
D13 0.00000400 sec
D16 0.00020000 sec
IN0 0.00003310 sec
TDav 1
SFO1 500.0421002 MHz
NUC1 1H
P1 10.32 usec
P2 20.64 usec
PLW1 15.00000000 W
SFO2 125.7427021 MHz
NUC2 13C
CPDPRG[2 garp
P3 13.00 usec
PCPD2 55.00 usec
PLW2 55.00000000 W
PLW12 2.88000011 W
GPNAM[1] SMSQ10.100
GPZ1 50.00 %
GPNAM[2] SMSQ10.100
GPZ2 30.00 %
GPNAM[3] SMSQ10.100
GPZ3 40.10 %
P16 1000.00 usec
F1 - Acquisition parameters
TD 128
SFO1 125.7427 MHz
FIDRES 236.027191 Hz
SW 120.132 ppm
FnMODE QF
F2 - Processing parameters
SI 1024
SF 500.0399851 MHz
WDW QSINE
SSB 0
LB 0 Hz
GB 0
PC 1.00
F1 - Processing parameters
SI 512
MC2 QF
SF 125.7351385 MHz
WDW QSINE
SSB 0
LB 0 Hz
GB 0

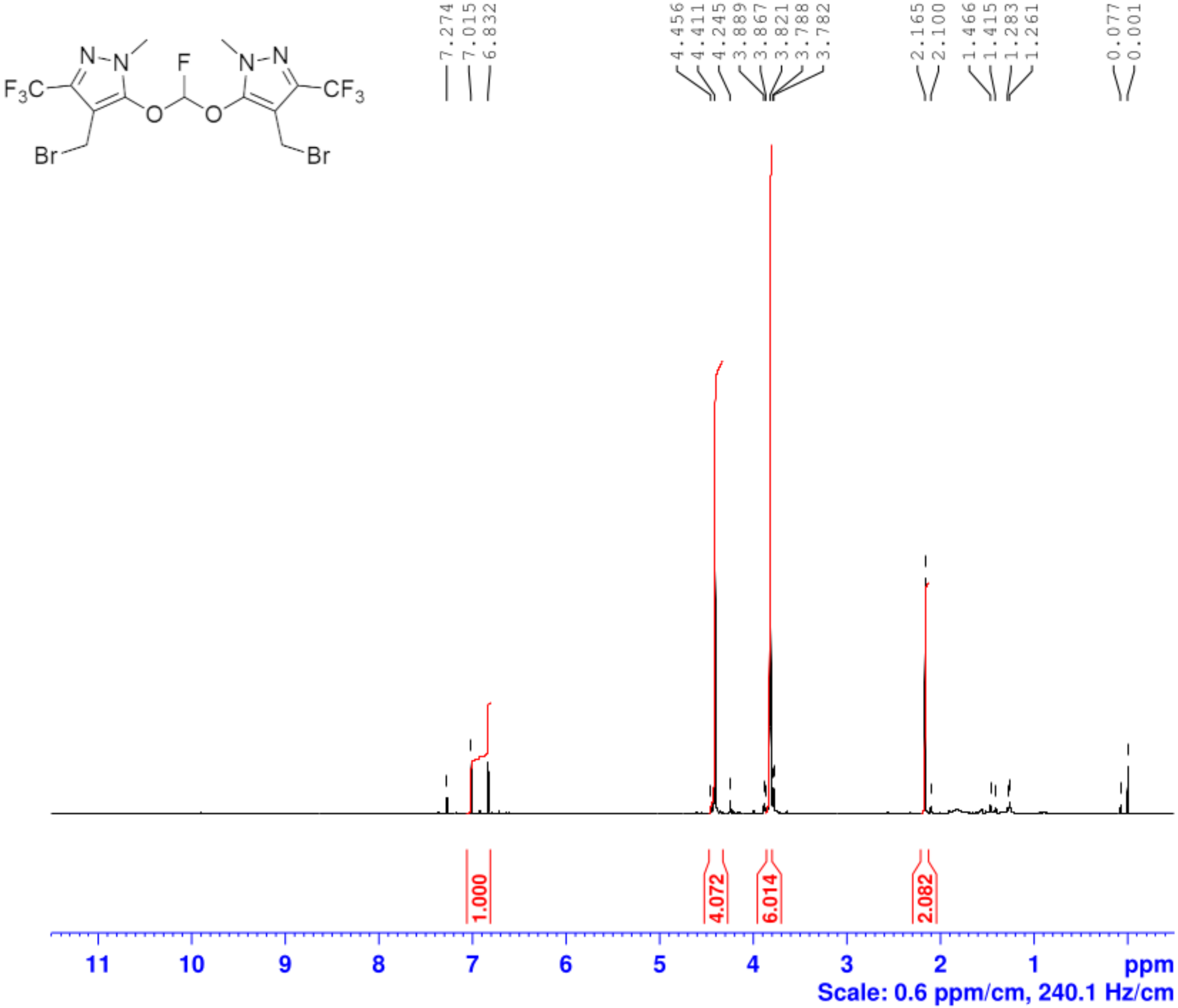

NAME	P7K4.5.20	
EXPNO	1	
PROCNO	1	
Date_	20200504	
Time	11.18	
INSTRUM	spect	
PROBHD	5 mm PABBO BB-	
PULPROG	zg30	
TD	65536	
SOLVENT	CDCl3	
NS	24	
DS	0	
SWH	7183.908	Hz
FIDRES	0.109618	Hz
AQ	4.5613556	sec
RG	128	
DW	69.600	use
DE	6.50	use
TE	301.0	K
D1	0.10000000	sec
TD0	1	

======== CHANNEL f1 ========

NUC1	1H	
P1	10.10	use
PL1	0.00	dB
SFO1	400.1032008	MHz
SI	32768	
SF	400.1000046	MHz
WDW	EM	
SSB	0	
LB	0.00	Hz
GB	0	
PC	1.00	

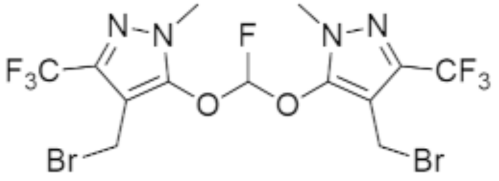

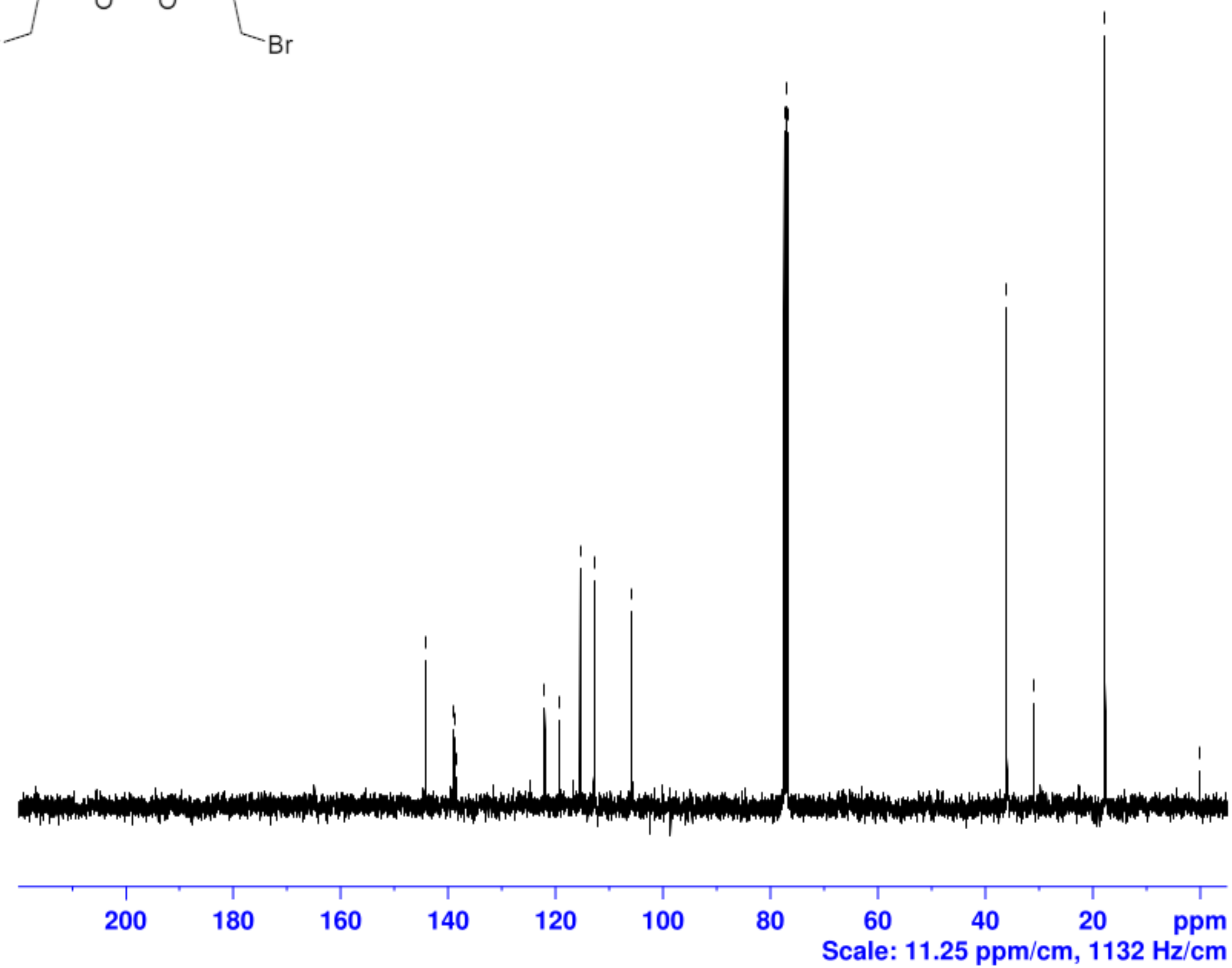

NAME	P7K4.5.20
EXPNO	3
PROCNO	1
Date_	20200504
Time	11.27
INSTRUM	spect
PROBHD	5 mm PABBO BB-
PULPROG	zgdc30
TD	32768
SOLVENT	CDCl3
NS	2375
DS	0
SWH	24154.590 Hz
FIDRES	0.737140 Hz
AQ	0.6783476 se
RG	71.8
DW	20.700 us
DE	29.57 us
TE	302.3 K
D1	0.10000000 se
D11	0.03000000 se
TD0	1

======== CHANNEL f1 ======

NUC1	13C
P1	9.20 us
PL1	3.00 dB
SFO1	100.6152865 MH

======== CHANNEL f2 ======

CPDPRG2	waltz16
NUC2	1H
PCPD2	80.00 us
PL2	0.00 dB
PL12	18.00 dB
SFO2	400.1009202 MH
SI	32768
SF	100.6052190 MH
WDW	EM
SSB	0
LB	2.00 Hz
GB	0
PC	1.00

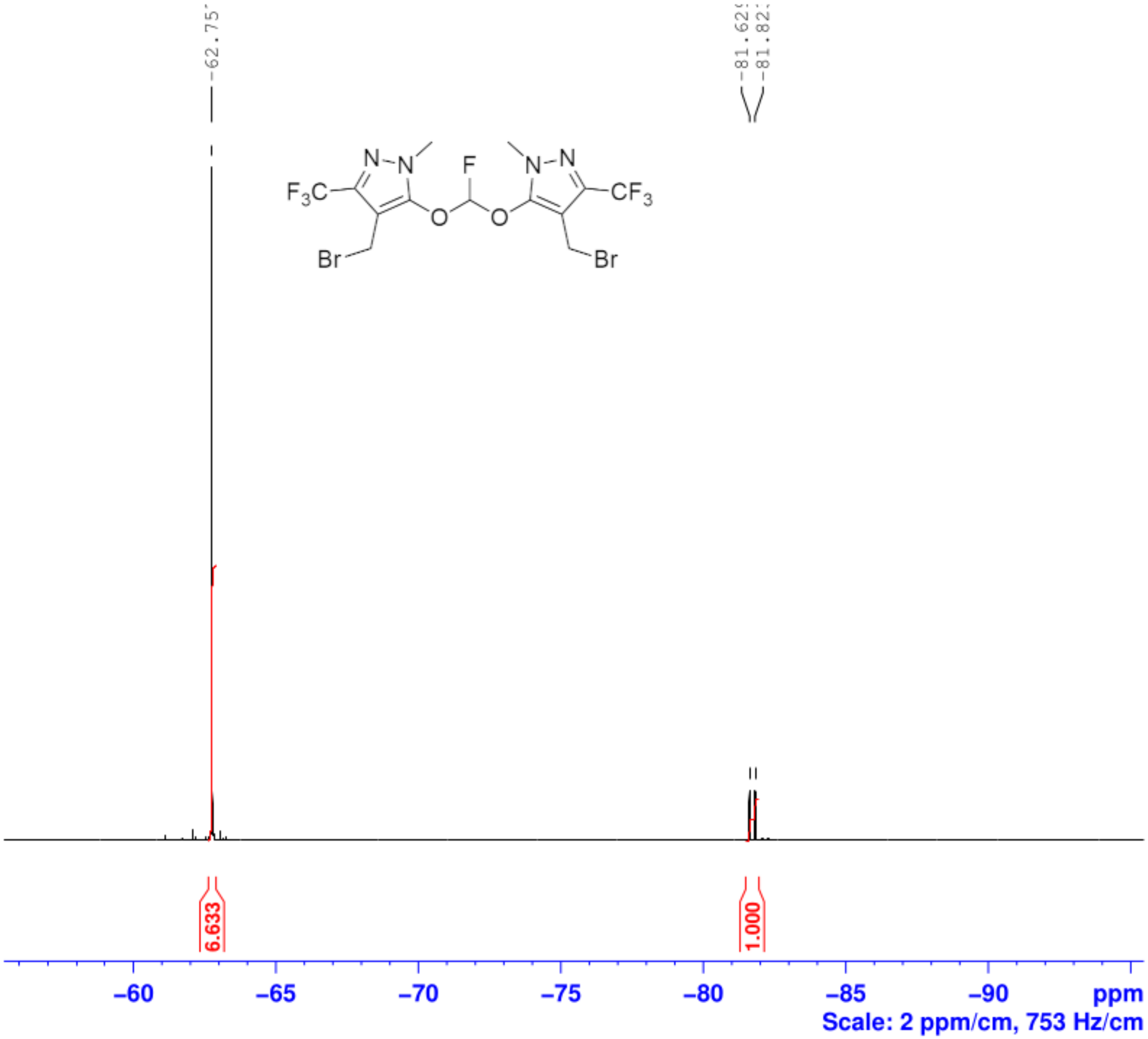

NAME	P7K4.5.20
EXPNO	2
PROCNO	1
Date_	20200504
Time	11.19
INSTRUM	spect
PROBHD	5 mm PABBO BB-
PULPROG	zg30
TD	32768
SOLVENT	CDCl3
NS	24
DS	0
SWH	15060.241 Hz
FIDRES	0.459602 Hz
AQ	1.0879476 sec
RG	71.8
DW	33.200 usec
DE	6.32 usec
TE	301.0 K
D1	0.10000000 sec
TD0	1

======== CHANNEL f1 ========

NUC1	19F
P1	7.90 usec
PL1	0.00 dB
SFO1	376.4419017 MHz
SI	32768
SF	376.4703092 MHz
WDW	EM
SSB	0
LB	0.50 Hz
GB	0
PC	1.00

HMBC

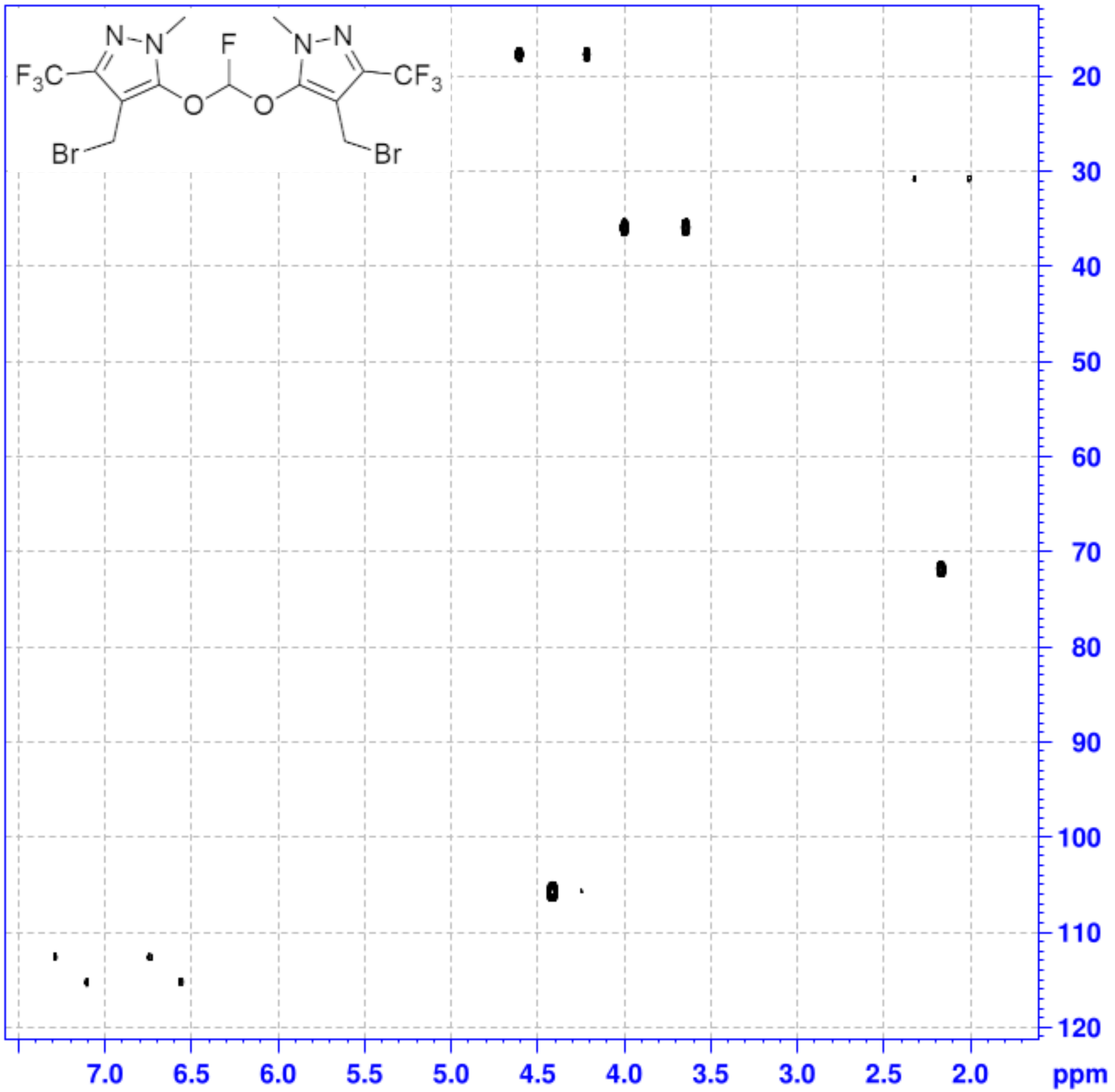


```
NAME            P7K4.5.20
EXPNO                   6
PROCNO                  1
Date_            20200504
Time                12.26
INSTRUM             spect
PROBHD   5 mm PABBO BB-
PULPROG      hmbcgplpndqf
TD                   1024
SOLVENT             CDCl3
NS                     10
DS                      2
SWH              2394.636 Hz
FIDRES           2.338512 Hz
AQ              0.2138612 sec
RG                  46341
DW                208.800 usec
DE                 241.25 usec
TE                  302.2 K
CNST2         145.0000000
CNST13          8.0000000
D0             0.00000300 sec
D1             1.00000000 sec
D2             0.00344828 sec
D6             0.06250000 sec
D16            0.00020000 sec
IN0            0.00003680 sec

======== CHANNEL f1 ========
NUC1                   1H
P1                  10.10 usec
P2                  20.20 usec
PL1                  0.00 dB
SFO1          400.1018405 MHz

======== CHANNEL f2 ========
NUC2                  13C
P3                   9.20 usec
PL2                  0.00 dB
SFO2          100.6132744 MHz

====== GRADIENT CHANNEL =====
GPNAM1           sine.100
GPNAM2           sine.100
GPNAM3           sine.100
GPZ1                50.00 %
GPZ2                30.00 %
GPZ3                40.10 %
P16               1000.00 usec
ND0                     2
TD                    128
SFO1             100.6133 MHz
FIDRES         106.115562 Hz
SW                135.000 ppm
FnMODE                 QF
SI                   1024
SF            400.1000046 MHz
WDW                 QSINE
SSB                     0
LB                   0.00 Hz
GB                      0
PC                   1.00
SI                    512
MC2                    QF
SF            100.6052190 MHz
WDW                 QSINE
SSB                     0
LB                   0.00 Hz
GB                      0
```

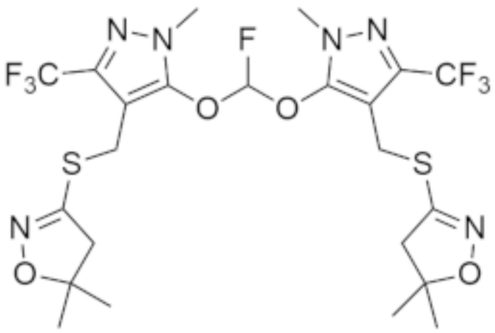

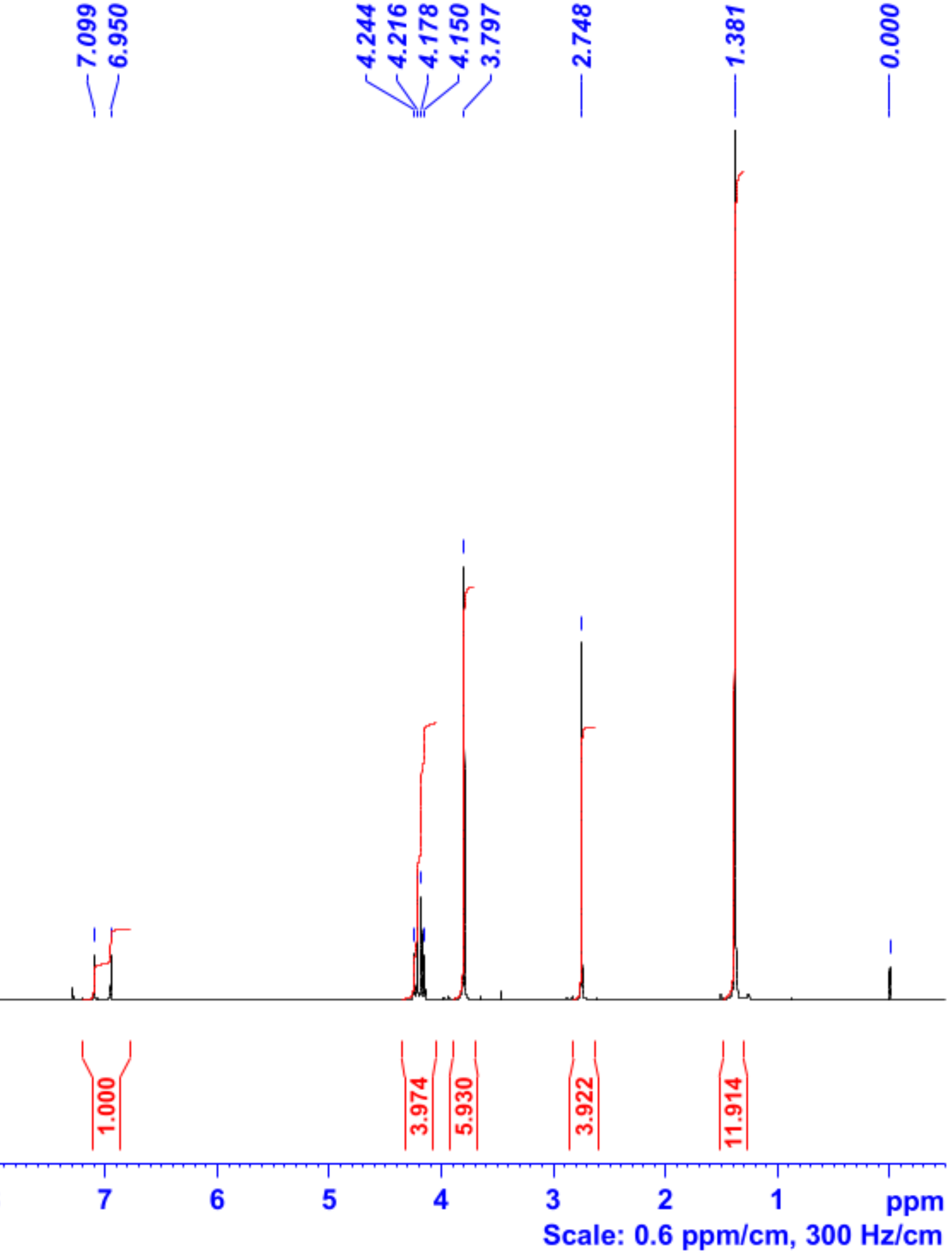


```
Current Data Parameters
NAME              P7K4.3.21
EXPNO                     1
PROCNO                    1

F2 - Acquisition Parameters
Date_              20210304
Time                  11.00 h
INSTRUM               spect
PROBHD   Z8420_0082 (PH
PULPROG                zg30
TD                    65536
SOLVENT               CDCl3
NS                       24
DS                        0
SWH                7002.801 Hz
FIDRES             0.213709 Hz
AQ                4.6792703 sec
RG                      128
DW                   71.400 usec
DE                     6.50 usec
TE                    300.0 K
D1               0.10000000 sec
TD0                       1
SFO1            500.0430002 MHz
NUC1                     1H
P1                    10.32 usec
PLW1            15.00000000 W

F2 - Processing parameters
SI                    65536
SF              500.0399969 MHz
WDW                      EM
SSB        0
LB         0 Hz
GB         0
PC                     1.00
```

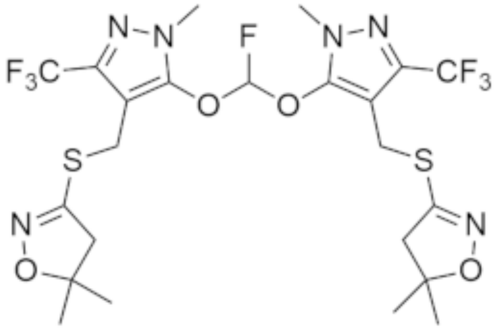

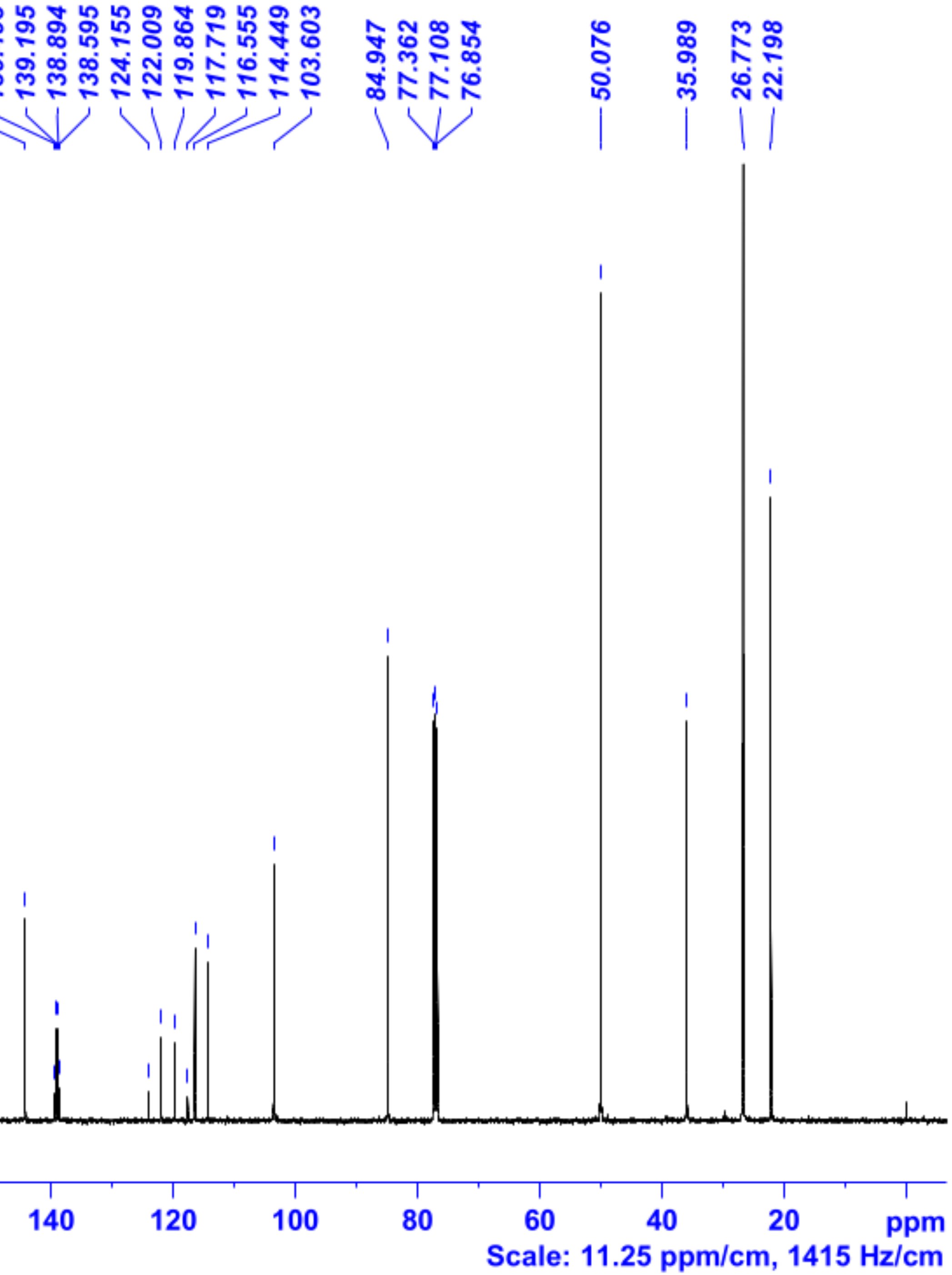


```
Current Data Parameters
NAME              P7K4.3.21
EXPNO                     2
PROCNO                    1

F2 - Acquisition Parameters
Date_              20210304
Time                11.31 h
INSTRUM               spect
PROBHD    Z8420_0082 (PH
PULPROG             zgdc30
TD                    65536
SOLVENT               CDCl3
NS                     1500
DS                        0
SWH              29761.904 Hz
FIDRES            0.908261 Hz
AQ               1.1010048 sec
RG                     2050
DW                   16.800 usec
DE                     6.50 usec
TE                    300.0 K
D1               0.10000000 sec
D11              0.03000000 sec
TD0                       1
SFO1           125.7477315 MHz
NUC1                    13C
P1                    13.00 usec
PLW1            55.00000000 W
SFO2           500.0425002 MHz
NUC2                     1H
CPDPRG[2            waltz16
PCPD2                 80.00 usec
PLW2            15.00000000 W
PLW12            0.24962001 W

F2 - Processing parameters
SI                    32768
SF             125.7351521 MHz
WDW                      EM
SSB        0
LB                     2.00 Hz
GB         0
PC                     1.00
```

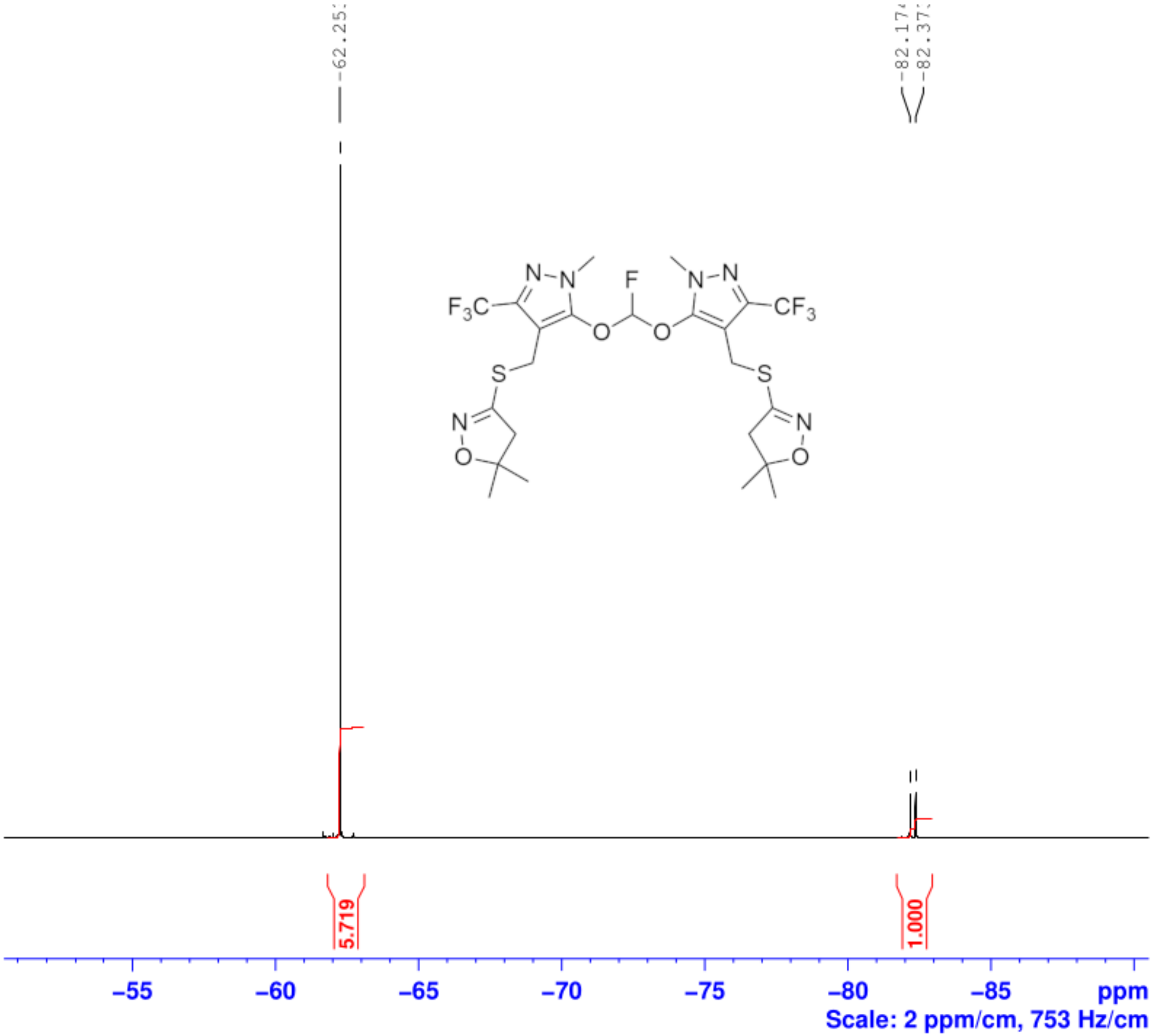

NAME	P7K4.3.21
EXPNO	5
PROCNO	1
Date_	20210304
Time	12.11
INSTRUM	spect
PROBHD	5 mm PABBO BB-
PULPROG	zg30
TD	32768
SOLVENT	CDCl3
NS	24
DS	0
SWH	15060.241 Hz
FIDRES	0.459602 Hz
AQ	1.0879476 se
RG	1625.5
DW	33.200 us
DE	6.32 us
TE	299.9 K
D1	0.10000000 se
TD0	1

======== CHANNEL f1 ======

NUC1	19F
P1	7.90 us
PL1	0.00 dB
SFO1	376.4437841 MH
SI	32768
SF	376.4703301 MH
WDW	EM
SSB	0
LB	0.50 Hz
GB	0
PC	1.00

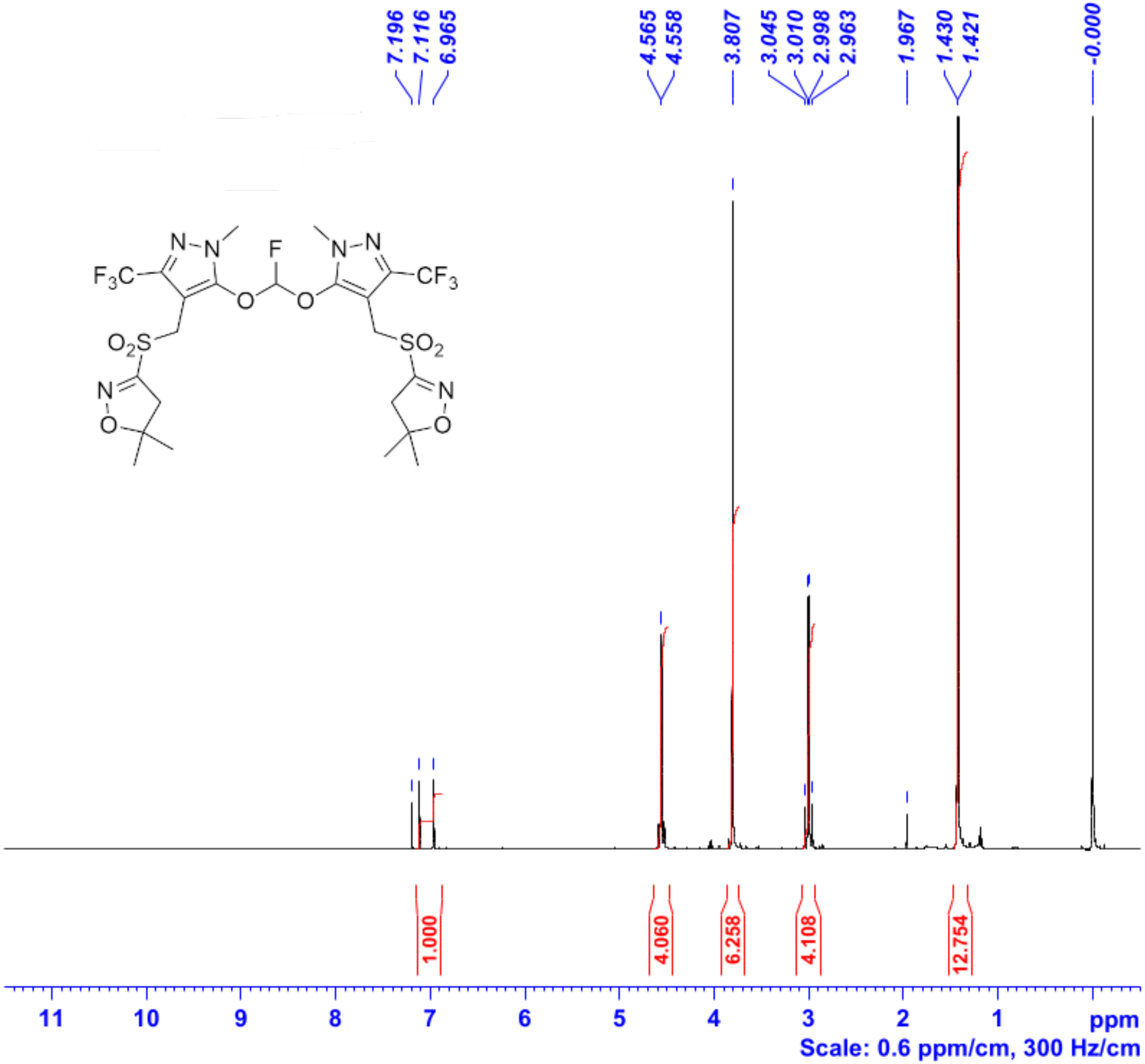


```
Current Data Parameters
NAME            P7K3.11.20
EXPNO                    1
PROCNO                   1

F2 - Acquisition Parameters
Date_             20201103
Time_              16.47 h
INSTRUM              spect
PROBHD     Z8420_0082 (PH
PULPROG               zg30
TD                   65536
SOLVENT              CDCl3
NS                      48
DS                       0
SWH            7002.801 Hz
FIDRES         0.213709 Hz
AQ            4.6792703 sec
RG                     181
DW              71.400 usec
DE                6.50 usec
TE                 300.0 K
D1          0.10000000 sec
TD0                      1
SFO1        500.0430002 MHz
NUC1                    1H
P1               10.32 usec
PLW1        15.00000000 W

F2 - Processing parameters
SI                   65536
SF          500.0400437 MHz
WDW                     EM
SSB         0
LB          0 Hz
GB          0
PC                    1.00
```

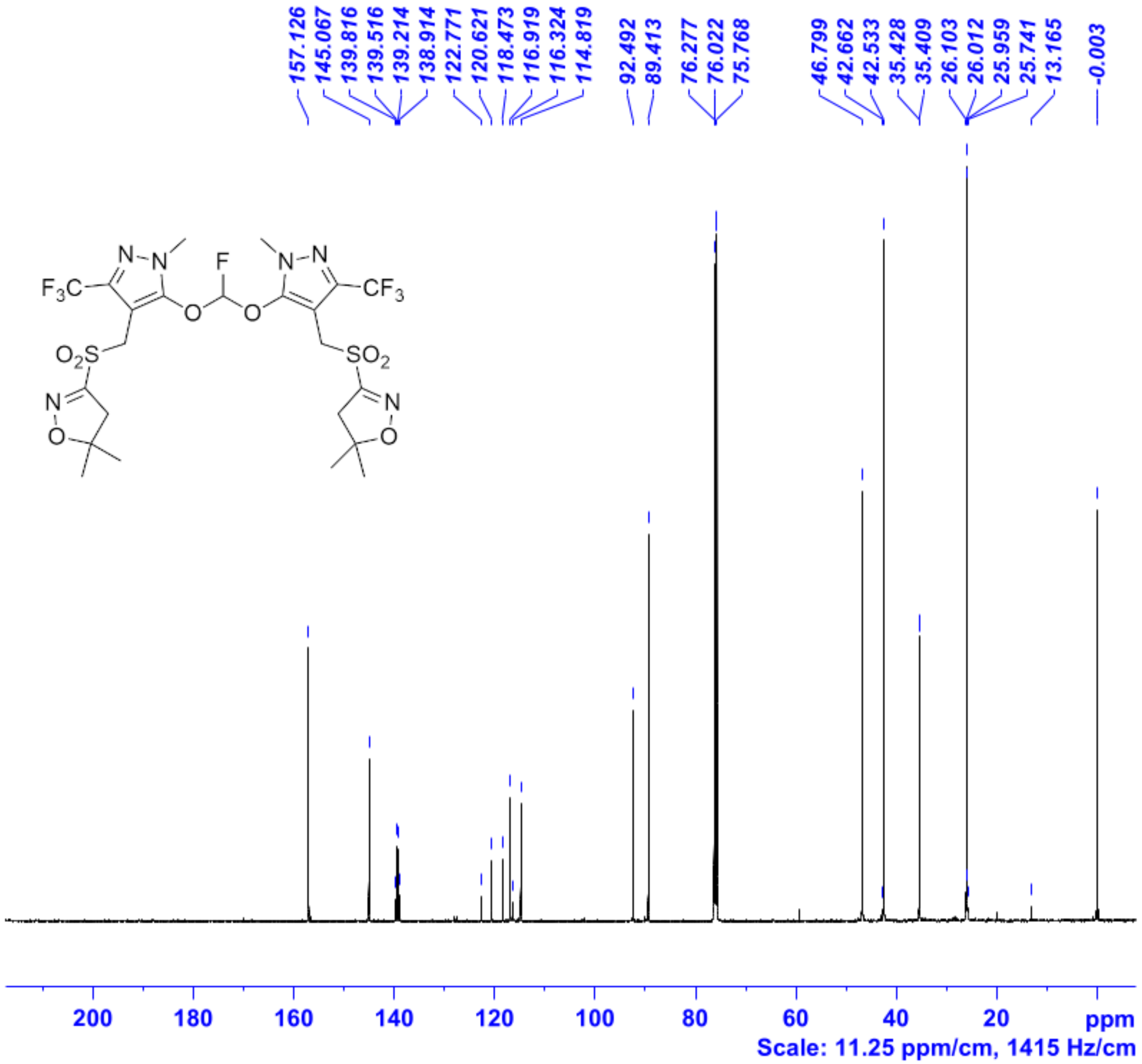


```
Current Data Parameters
NAME          P7K3.11.20
EXPNO                  3
PROCNO                 1

F2 - Acquisition Parameters
Date_           20201104
Time_             7.59 h
INSTRUM            spect
PROBHD   Z8420_0082 (PH
PULPROG          zgdc30
TD                65536
SOLVENT           CDCl3
NS                33323
DS                    0
SWH           29761.904 Hz
FIDRES         0.908261 Hz
AQ            1.1010048 sec
RG                 2050
DW               16.800 usec
DE                 6.50 usec
TE                300.0 K
D1           0.10000000 sec
D11          0.03000000 sec
TD0                   1
SFO1        125.7477315 MHz
NUC1                13C
P1                13.00 usec
PLW1        55.00000000 W
SFO2        500.0425002 MHz
NUC2                 1H
CPDPRG[2        waltz16
PCPD2             80.00 usec
PLW2        15.00000000 W
PLW12        0.24962001 W

F2 - Processing parameters
SI                32768
SF          125.7352853 MHz
WDW                  EM
SSB      0
LB                 2.00 Hz
GB       0
PC                 1.00
```

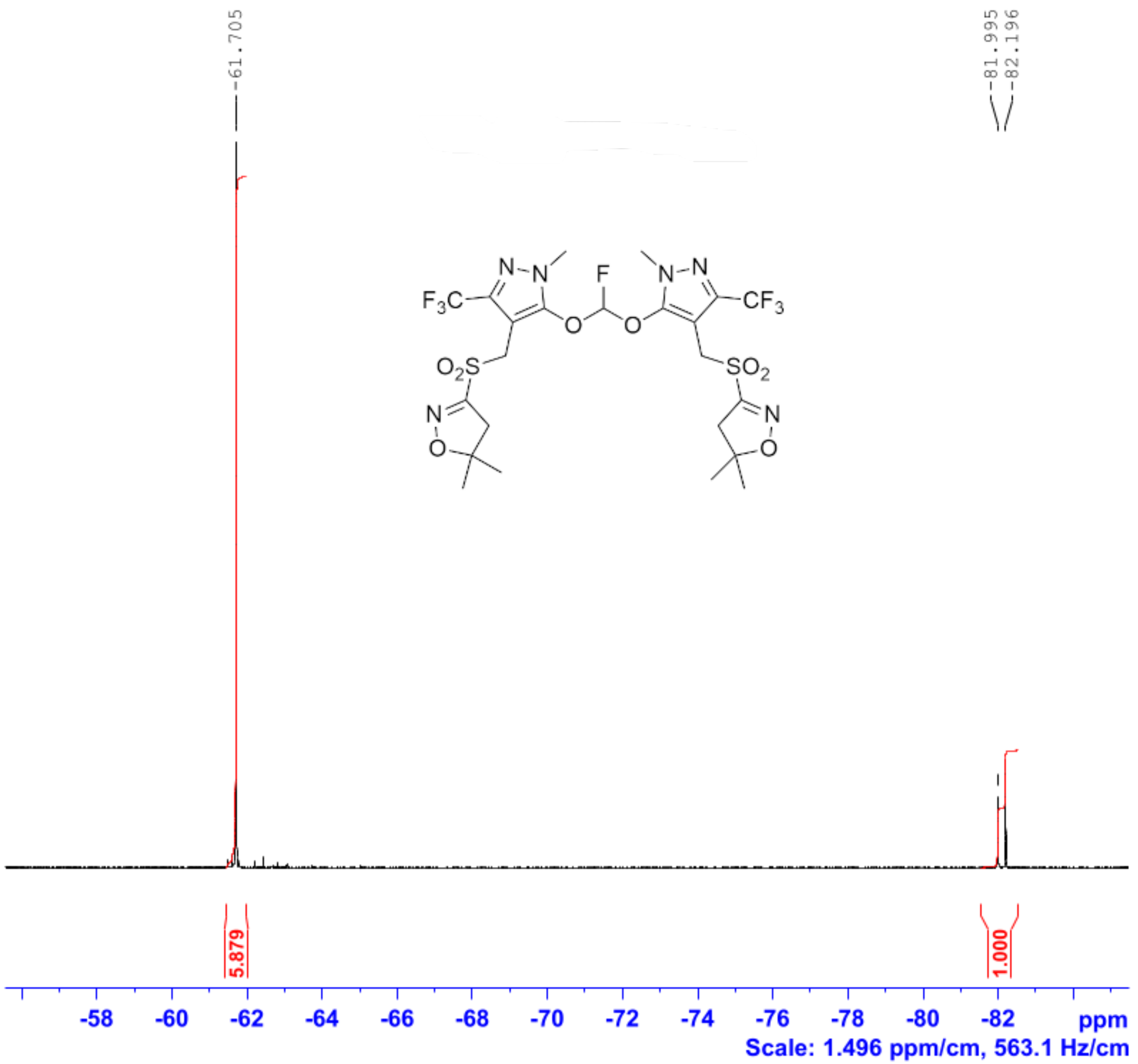


```
Current Data Parameters
NAME              P7K3.11.20
EXPNO                      5
PROCNO                     1

F2 - Acquisition Parameters
Date_               20201103
Time                   14.34
INSTRUM                spect
PROBHD     5 mm PABBO BB-
PULPROG                 zg30
TD                     32768
SOLVENT                CDCl3
NS                         8
DS                         0
SWH            11261.262 Hz
FIDRES          0.343666 Hz
AQ             1.4548992 sec
RG                      3251
DW               44.400 usec
DE                 6.32 usec
TE                 301.0 K
D1            0.10000000 sec
TD0                        1

======== CHANNEL f1 ========
NUC1                     19F
P1                 7.90 usec
PL1         0 dB
SFO1         376.4437841 MHz

F2 - Processing parameters
SI                     32768
SF           376.4703301 MHz
WDW                       EM
SSB         0
LB                   0.50 Hz
GB          0
PC                      1.00
```

HMBC

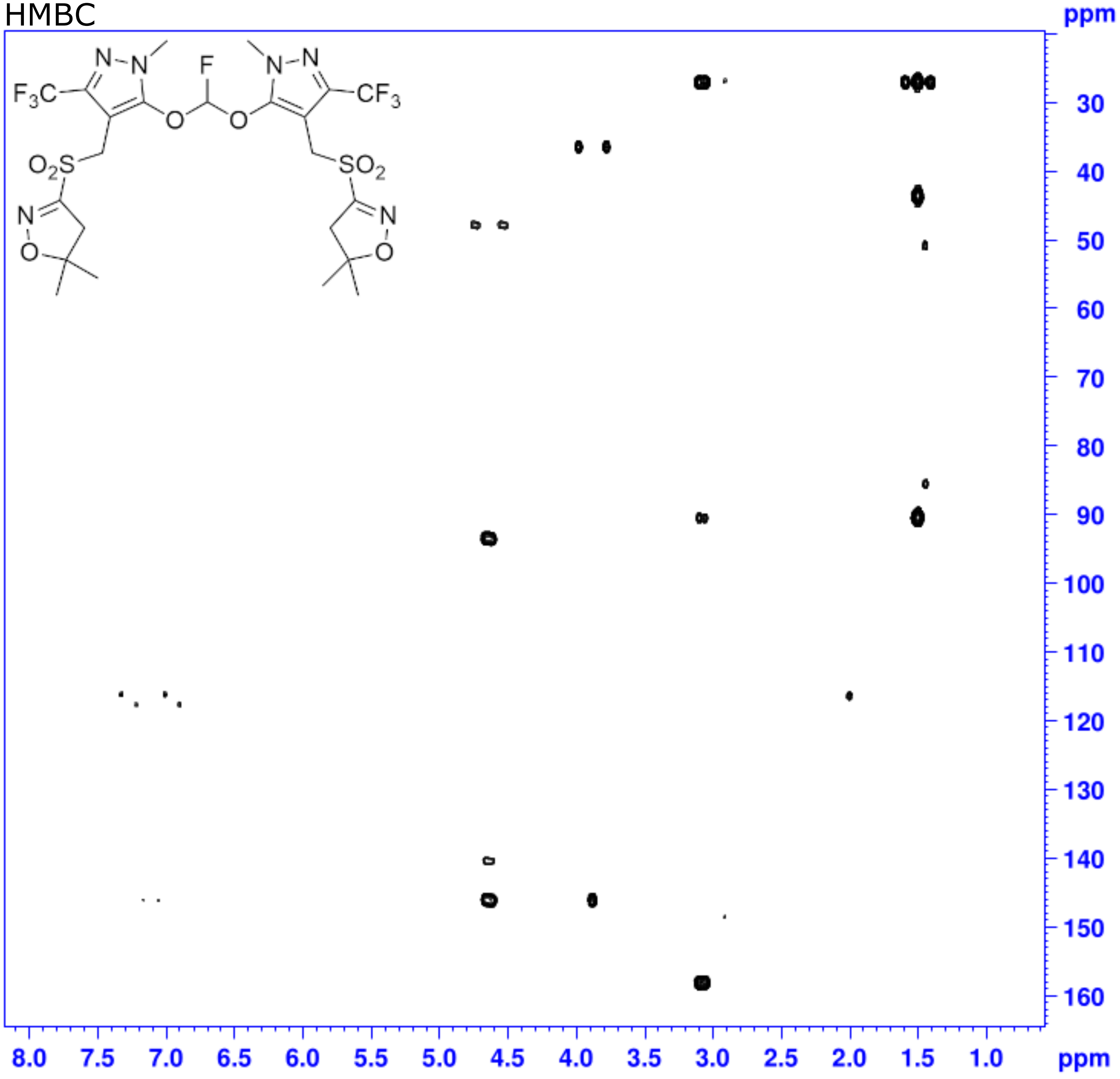

Current Data Parameters
NAME P7K3.9.19
EXPNO 6
PROCNO 1

F2 - Acquisition Parameters
Date_ 20190903
Time 9.20 h
INSTRUM spect
PROBHD Z44908_0030 (C
PULPROG hmbcgplpndqf
TD 1024
SOLVENT CDCl3
NS 4
DS 2
SWH 5326.705 Hz
FIDRES 10.403720 Hz
AQ 0.0961195 sec
RG 2050
DW 93.867 usec
DE 10.00 usec
TE 300.0 K
CNST2 145.0000000
CNST13 8.0000000
D0 0.00000300 sec
D1 1.00000000 sec
D2 0.00344828 sec
D6 0.06250000 sec
D16 0.00020000 sec
IN0 0.00001960 sec
TDav 1
SFO1 700.4530820 MHz
NUC1 1H
P1 9.32 usec
P2 18.64 usec
PLW1 9.00000000 W
SFO2 176.1444968 MHz
NUC2 13C
P3 14.00 usec
PLW2 18.94499969 W
GPNAM[1] SINE.100
GPZ1 50.00 %
GPNAM[2] SINE.100
GPZ2 30.00 %
GPNAM[3] SINE.100
GPZ3 40.10 %
P16 1000.00 usec

F1 - Acquisition parameters
TD 128
SFO1 176.1445 MHz
FIDRES 398.596924 Hz
SW 144.825 ppm
FnMODE QF

F2 - Processing parameters
SI 1024
SF 700.4500162 MHz
WDW QSINE
SSB 0
LB 0 Hz
GB 0
PC 1.00

F1 - Processing parameters
SI 512
MC2 QF
SF 176.1282756 MHz
WDW QSINE
SSB 0
LB 0 Hz
GB 0

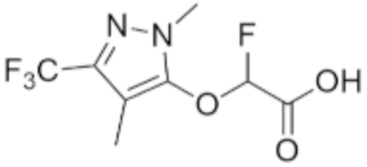

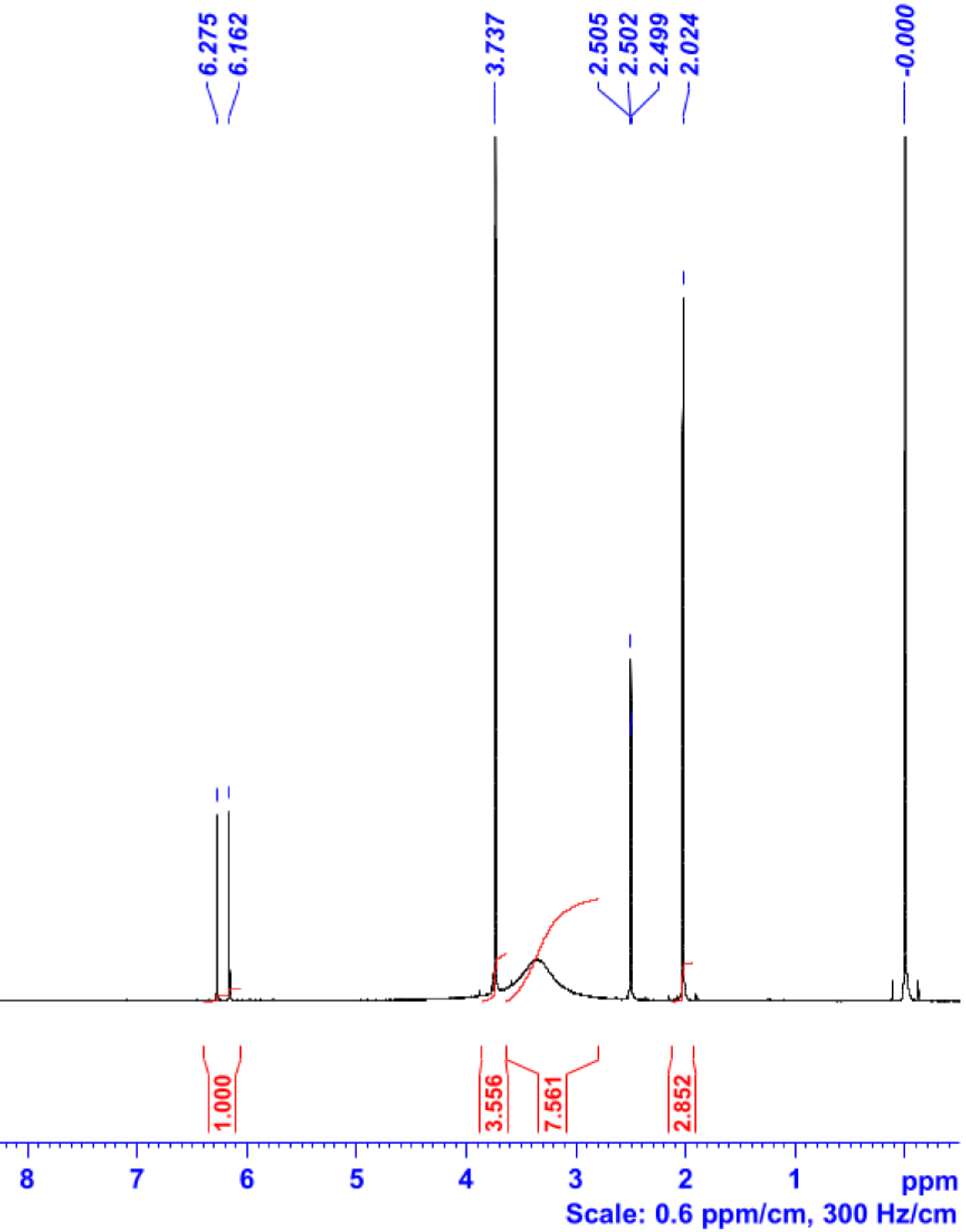


```
Current Data Parameters
NAME              P7K18.2.21
EXPNO                     11
PROCNO                     1

F2 - Acquisition Parameters
Date_               20210220
Time_                 3.36 h
INSTRUM                spect
PROBHD     Z8420_0082 (PH
PULPROG                 zg30
TD                     65536
SOLVENT                 DMSO
NS                        24
DS                         0
SWH                 7002.801 Hz
FIDRES              0.213709 Hz
AQ                 4.6792703 sec
RG                       512
DW                    71.400 usec
DE                      6.50 usec
TE                     300.0 K
D1                0.10000000 sec
TD0                        1
SFO1             500.0430002 MHz
NUC1                      1H
P1                     10.32 usec
PLW1             15.00000000 W

F2 - Processing parameters
SI                     65536
SF               500.0400026 MHz
WDW                       EM
SSB       0
LB                      0.20 Hz
GB        0
PC                      1.00
```

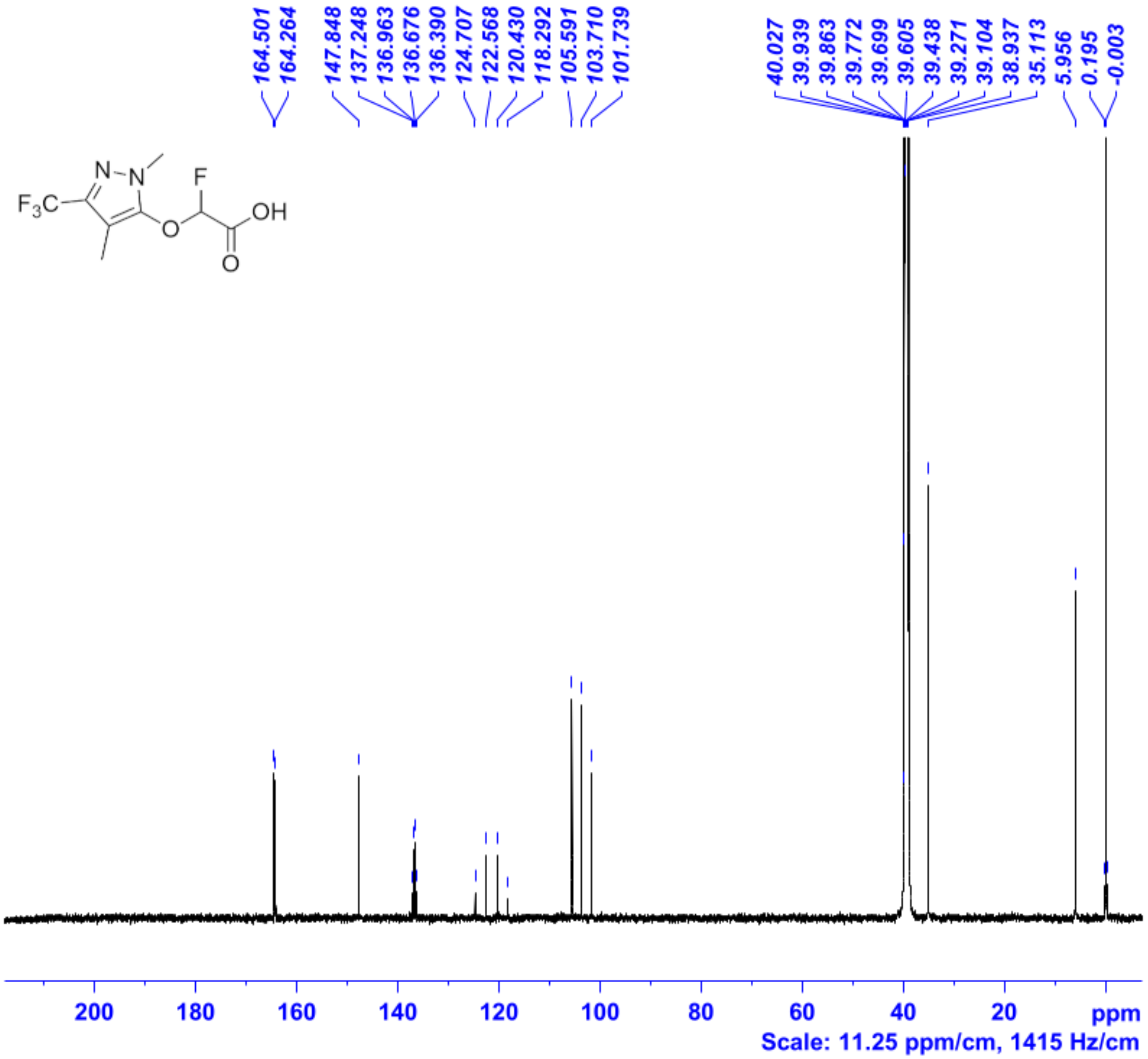

Current Data Parameters	
NAME	P7K18.2.21
EXPNO	10
PROCNO	1

F2 - Acquisition Parameters	
Date_	20210220
Time	3.34 h
INSTRUM	spect
PROBHD	Z8420_0082 (PH
PULPROG	zgdc30
TD	65536
SOLVENT	DMSO
NS	100000
DS	0
SWH	29761.904 Hz
FIDRES	0.908261 Hz
AQ	1.1010048 sec
RG	2050
DW	16.800 usec
DE	6.50 usec
TE	300.0 K
D1	0.10000000 sec
D11	0.03000000 sec
TD0	1
SFO1	125.7477315 MHz
NUC1	13C
P1	13.00 usec
PLW1	55.00000000 W
SFO2	500.0425002 MHz
NUC2	1H
CPDPRG[2	waltz16
PCPD2	80.00 usec
PLW2	15.00000000 W
PLW12	0.24962001 W

F2 - Processing parameters	
SI	32768
SF	125.7352290 MHz
WDW	EM
SSB	0
LB	2.00 Hz
GB	0
PC	1.00

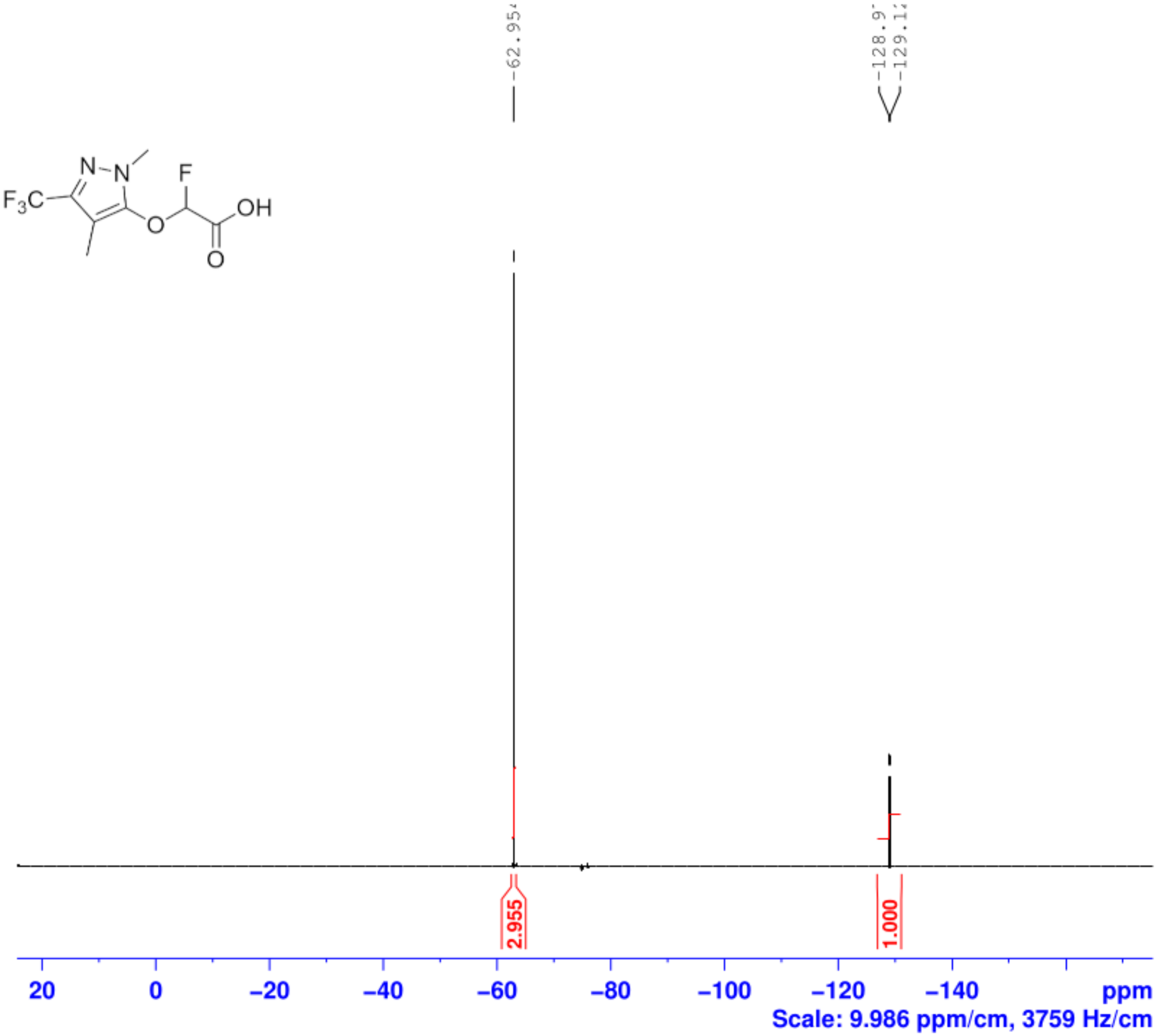

BRUKER

NAME	P7K2.9.19
EXPNO	1
PROCNO	1
Date_	20190902
Time	11.04
INSTRUM	spect
PROBHD	5 mm PABBO BB-
PULPROG	zg30
TD	32768
SOLVENT	CDCl3
NS	8
DS	0
SWH	75187.969 Hz
FIDRES	2.294555 Hz
AQ	0.2179572 se
RG	71.8
DW	6.650 us
DE	6.32 us
TE	300.2 K
D1	0.10000000 se
TD0	1

======== CHANNEL f1 ======

NUC1	19F
P1	7.90 us
PL1	0.00 dB
SFO1	376.4419017 MH
SI	32768
SF	376.4703092 MH
WDW	EM
SSB	0
LB	0.50 Hz
GB	0
PC	1.00

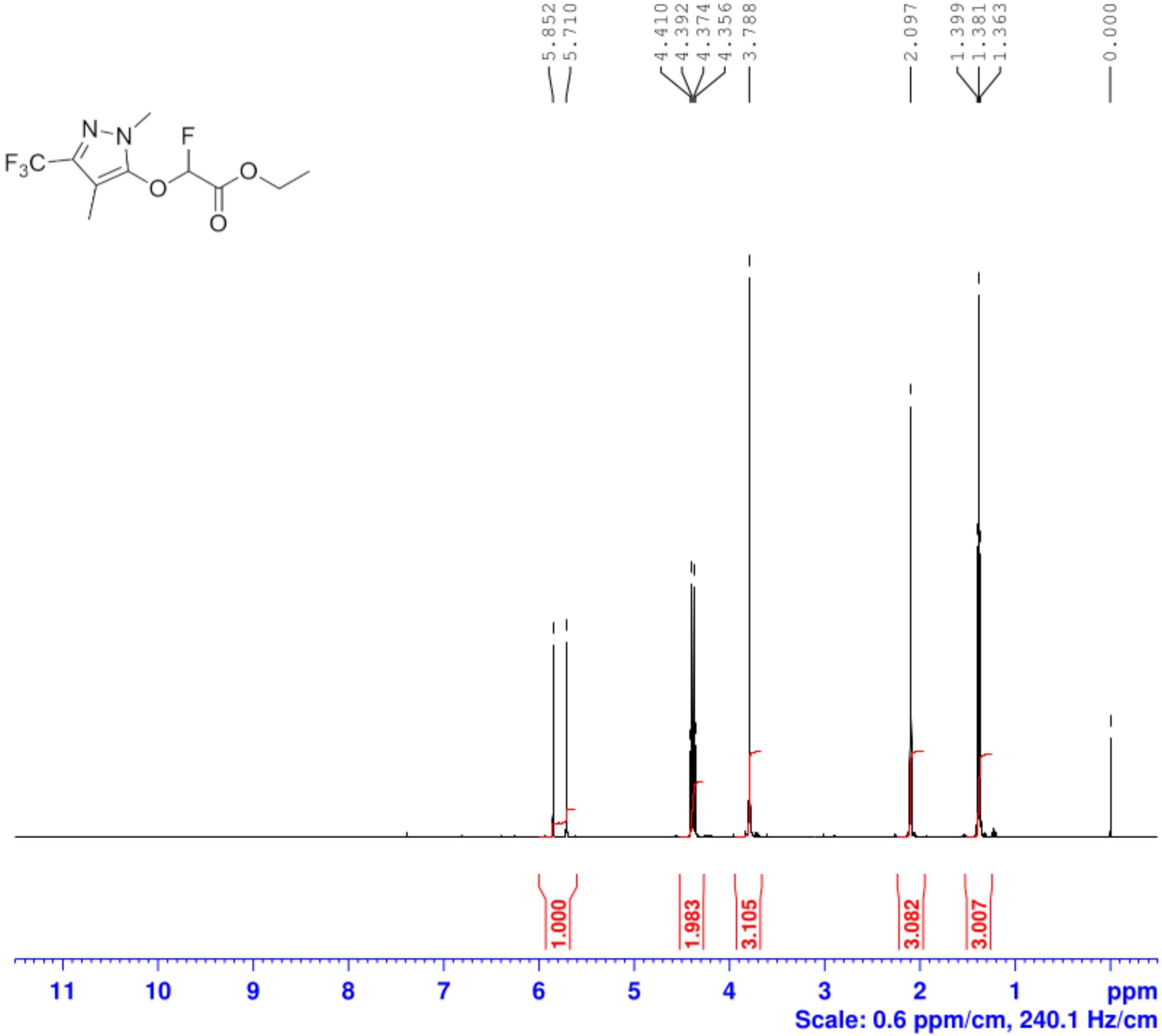

BRUKER

NAME	P7K15.2.21
EXPNO	15
PROCNO	1
Date_	20210216
Time	7.55
INSTRUM	spect
PROBHD	5 mm PABBO BB-
PULPROG	zg30
TD	65536
SOLVENT	CDCl3
NS	8
DS	0
SWH	7183.908 Hz
FIDRES	0.109618 Hz
AQ	4.5613556 sec
RG	20.2
DW	69.600 use
DE	6.50 use
TE	299.7 K
D1	0.10000000 sec
TD0	1

======== CHANNEL f1 ========

NUC1	1H
P1	10.10 use
PL1	0.00 dB
SFO1	400.1032008 MHz
SI	32768
SF	400.0999574 MHz
WDW	EM
SSB	0
LB	0.00 Hz
GB	0
PC	1.00

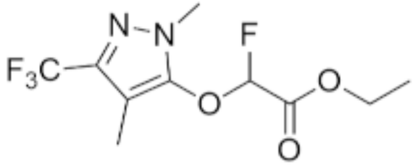

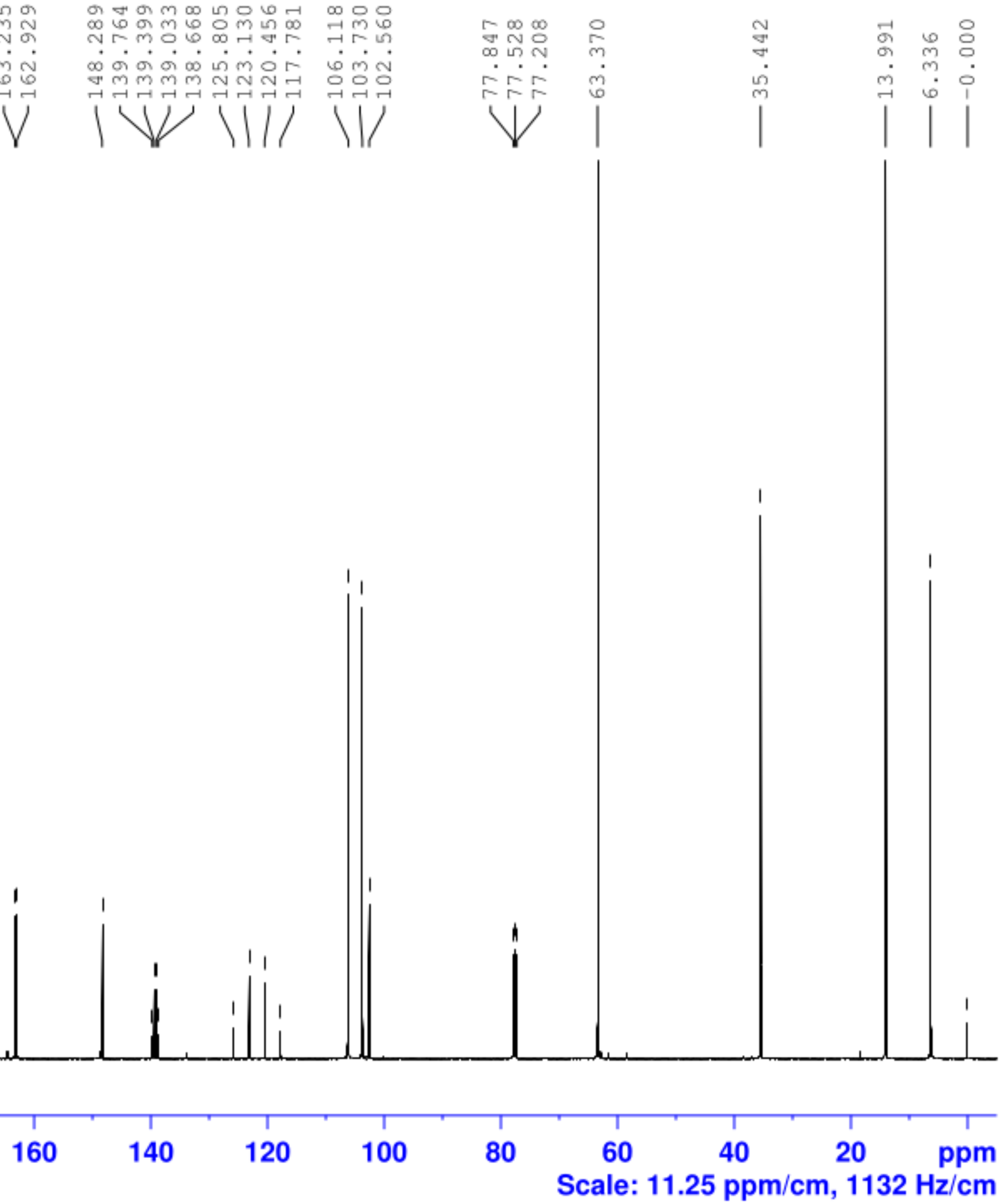

NAME	P7K15.2.21
EXPNO	14
PROCNO	1
Date_	20210216
Time	3.06
INSTRUM	spect
PROBHD	5 mm PABBO BB-
PULPROG	zgdc30
TD	32768
SOLVENT	CDCl3
NS	40000
DS	0
SWH	24154.590 Hz
FIDRES	0.737140 Hz
AQ	0.6783476 se
RG	71.8
DW	20.700 us
DE	29.57 us
TE	299.9 K
D1	0.10000000 se
D11	0.03000000 se
TD0	1

======== CHANNEL f1 ======

NUC1	13C
P1	9.20 us
PL1	3.00 dE
SFO1	100.6152865 MH

======== CHANNEL f2 ======

CPDPRG2	waltz16
NUC2	1H
PCPD2	80.00 us
PL2	0.00 dE
PL12	18.00 dE
SFO2	400.1009202 MH
SI	32768
SF	100.6051785 MH
WDW	EM
SSB	0
LB	2.00 Hz
GB	0
PC	1.00

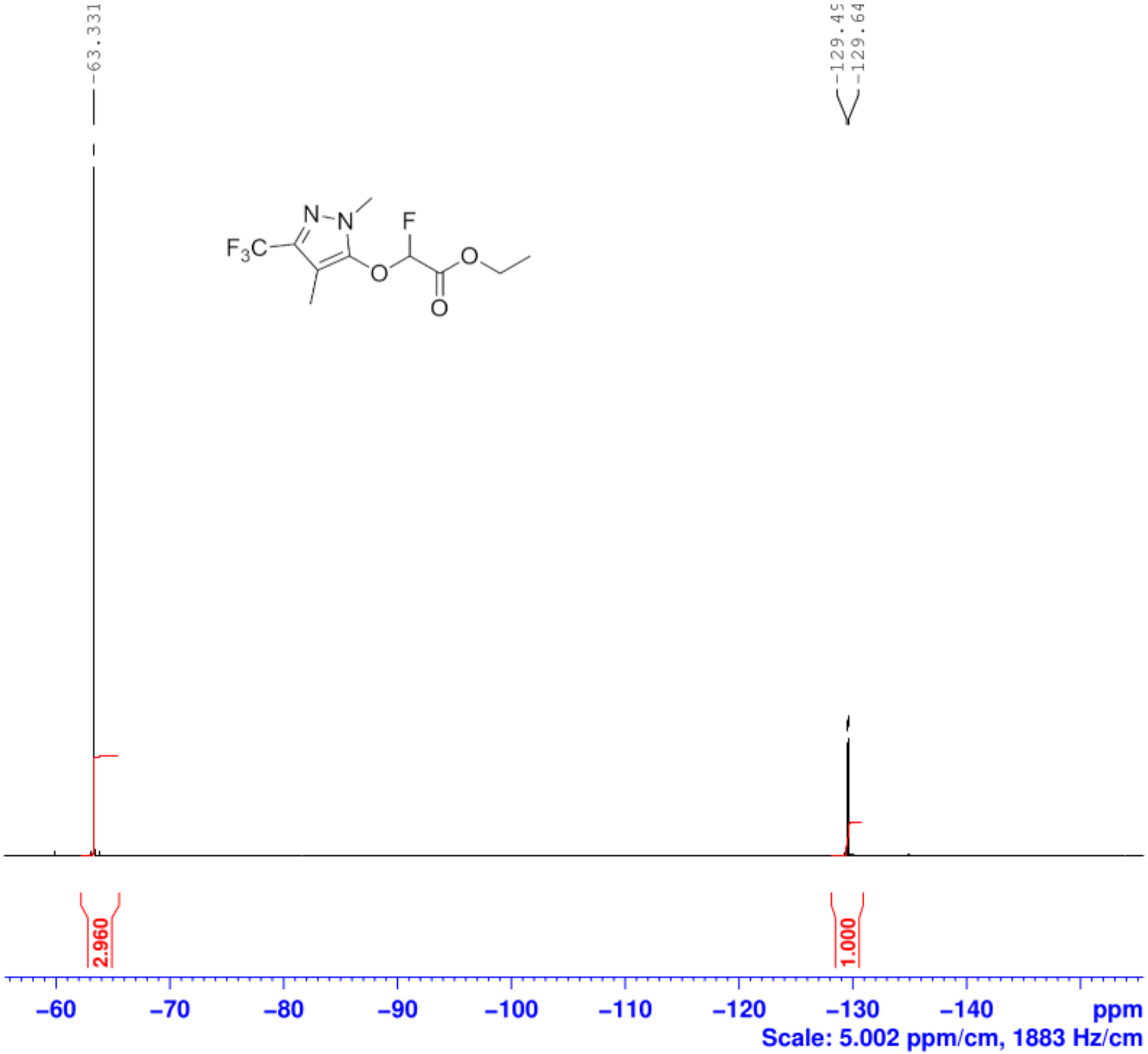

BRUKER

NAME	P7K15.2.21
EXPNO	6
PROCNO	1
Date_	20210215
Time	9.18
INSTRUM	spect
PROBHD	5 mm PABBO BB-
PULPROG	zg30
TD	32768
SOLVENT	CDCl3
NS	48
DS	0
SWH	37664.785 Hz
FIDRES	1.149438 Hz
AQ	0.4350452 se
RG	1290.2
DW	13.275 us
DE	6.32 us
TE	300.0 K
D1	0.10000000 se
TD0	1

======== CHANNEL f1 ======

NUC1	19F
P1	7.90 us
PL1	0.00 dB
SFO1	376.4306076 MH
SI	32768
SF	376.4703301 MH
WDW	EM
SSB	0
LB	0.50 Hz
GB	0
PC	1.00

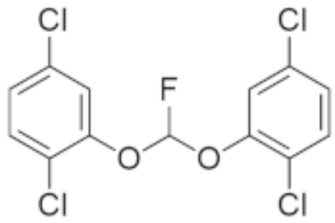

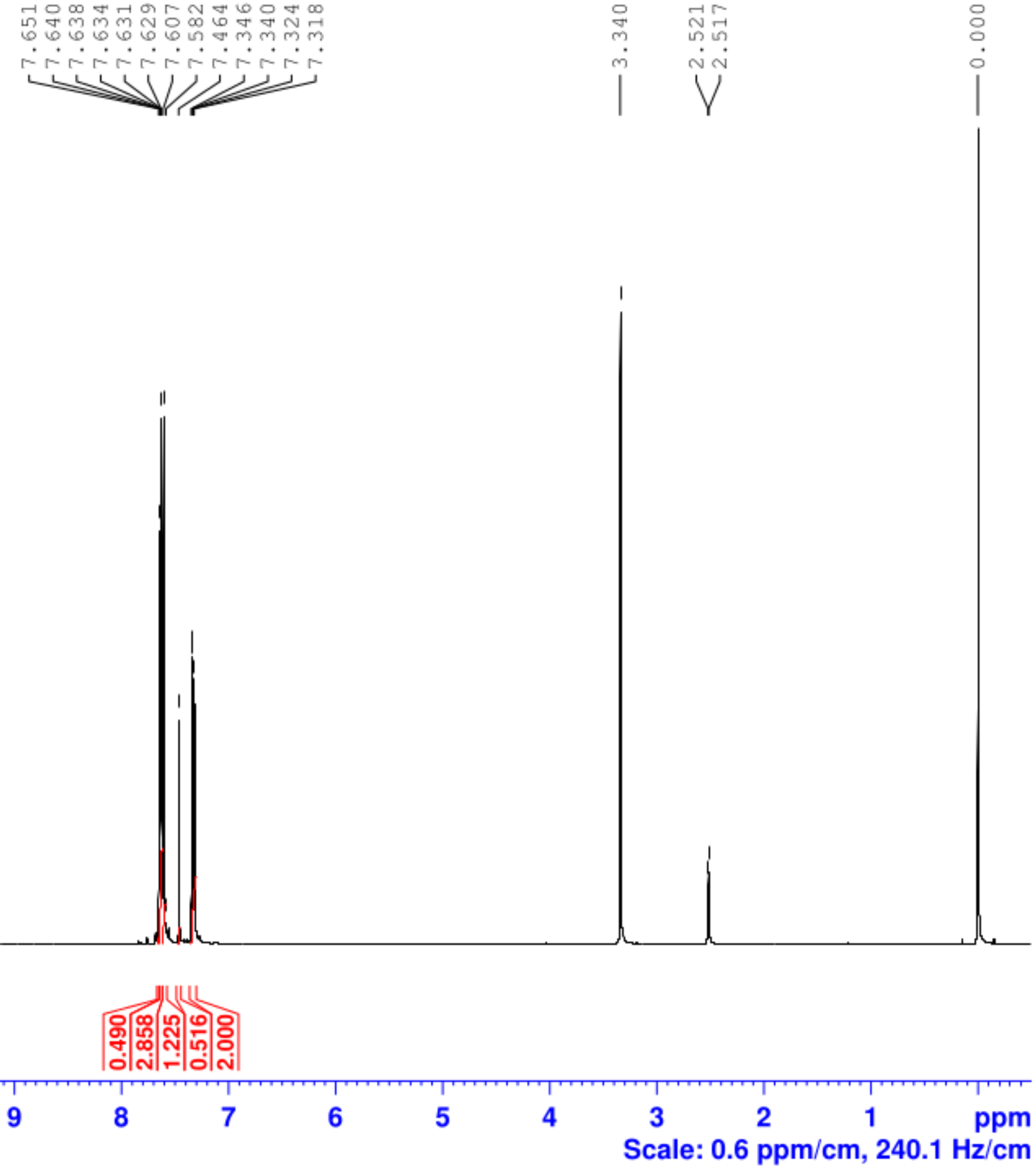

NAME	P7K26.1.21
EXPNO	4
PROCNO	1
Date_	20210126
Time	10.05
INSTRUM	spect
PROBHD	5 mm PABBO BB-
PULPROG	zg30
TD	65536
SOLVENT	DMSO
NS	24
DS	0
SWH	7183.908 Hz
FIDRES	0.109618 Hz
AQ	4.5613556 sec
RG	362
DW	69.600 use
DE	6.50 use
TE	299.9 K
D1	0.10000000 sec
TD0	1

======== CHANNEL f1 ========

NUC1	1H
P1	10.10 use
PL1	0.00 dB
SFO1	400.1032008 MHz
SI	32768
SF	400.0999959 MHz
WDW	EM
SSB	0
LB	0.30 Hz
GB	0
PC	1.00

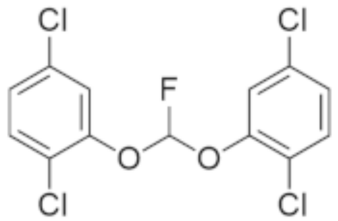

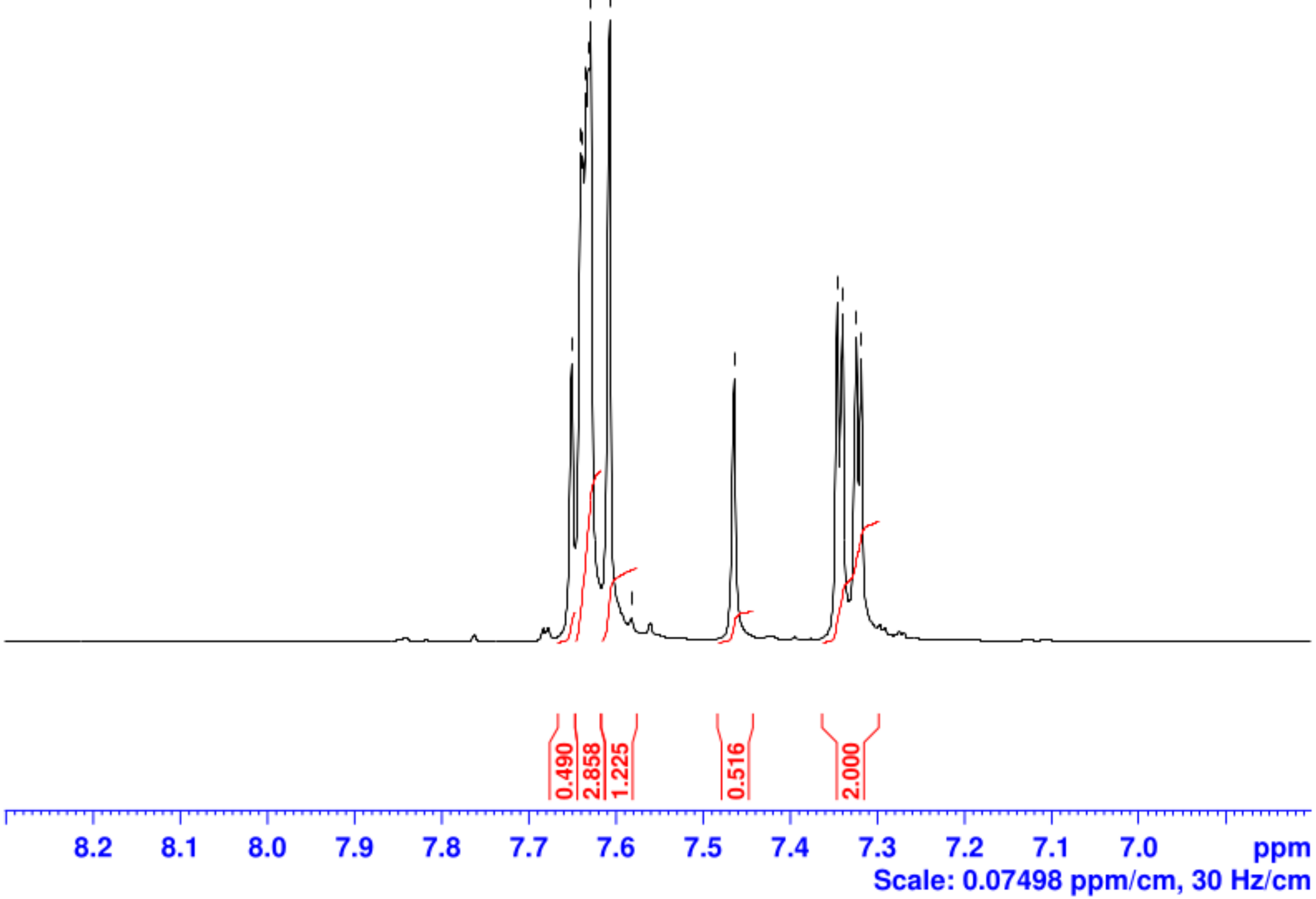

NAME	P7K26.1.21
EXPNO	4
PROCNO	1
Date_	20210126
Time	10.05
INSTRUM	spect
PROBHD	5 mm PABBO BB-
PULPROG	zg30
TD	65536
SOLVENT	DMSO
NS	24
DS	0
SWH	7183.908 Hz
FIDRES	0.109618 Hz
AQ	4.5613556 sec
RG	362
DW	69.600 use
DE	6.50 use
TE	299.9 K
D1	0.10000000 sec
TD0	1
======== CHANNEL f1 =======	
NUC1	1H
P1	10.10 use
PL1	0.00 dB
SFO1	400.1032008 MHz
SI	32768
SF	400.0999959 MHz
WDW	EM
SSB	0
LB	0.30 Hz
GB	0
PC	1.00

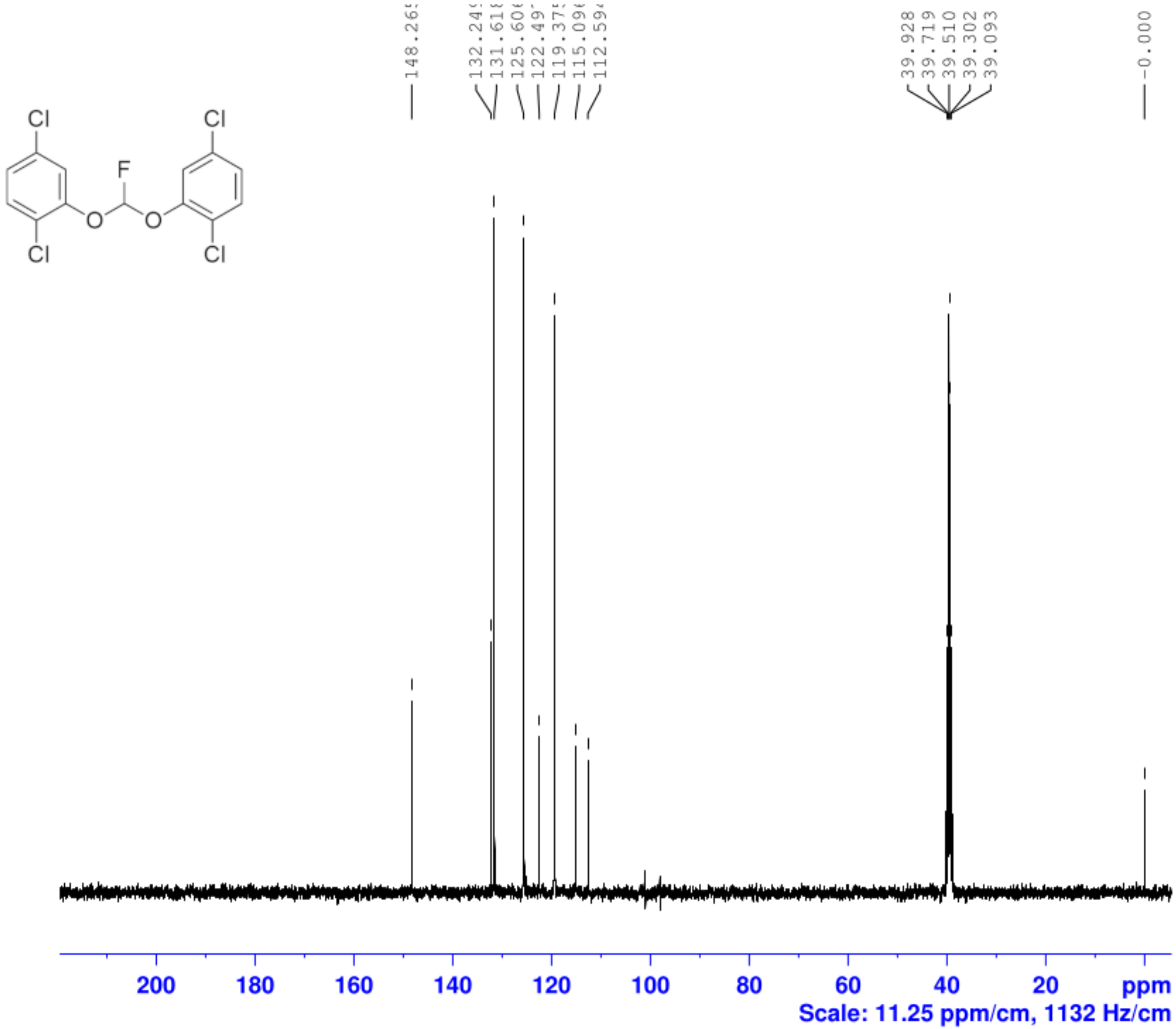

NAME	P7K26.1.21
EXPNO	7
PROCNO	1
Date_	20210126
Time	10.10
INSTRUM	spect
PROBHD	5 mm PABBO BB-
PULPROG	zgdc30
TD	32768
SOLVENT	DMSO
NS	1200
DS	0
SWH	24154.590 Hz
FIDRES	0.737140 Hz
AQ	0.6783476 se
RG	71.8
DW	20.700 us
DE	29.57 us
TE	300.5 K
D1	0.10000000 se
D11	0.03000000 se
TD0	1
======== CHANNEL f1 ======	
NUC1	13C
P1	9.20 us
PL1	3.00 dE
SFO1	100.6152865 MH
======== CHANNEL f2 ======	
CPDPRG2	waltz16
NUC2	1H
PCPD2	80.00 us
PL2	0.00 dE
PL12	18.00 dE
SFO2	400.1009202 MH
SI	32768
SF	100.6052769 MH
WDW	EM
SSB	0
LB	2.00 Hz
GB	0
PC	1.00

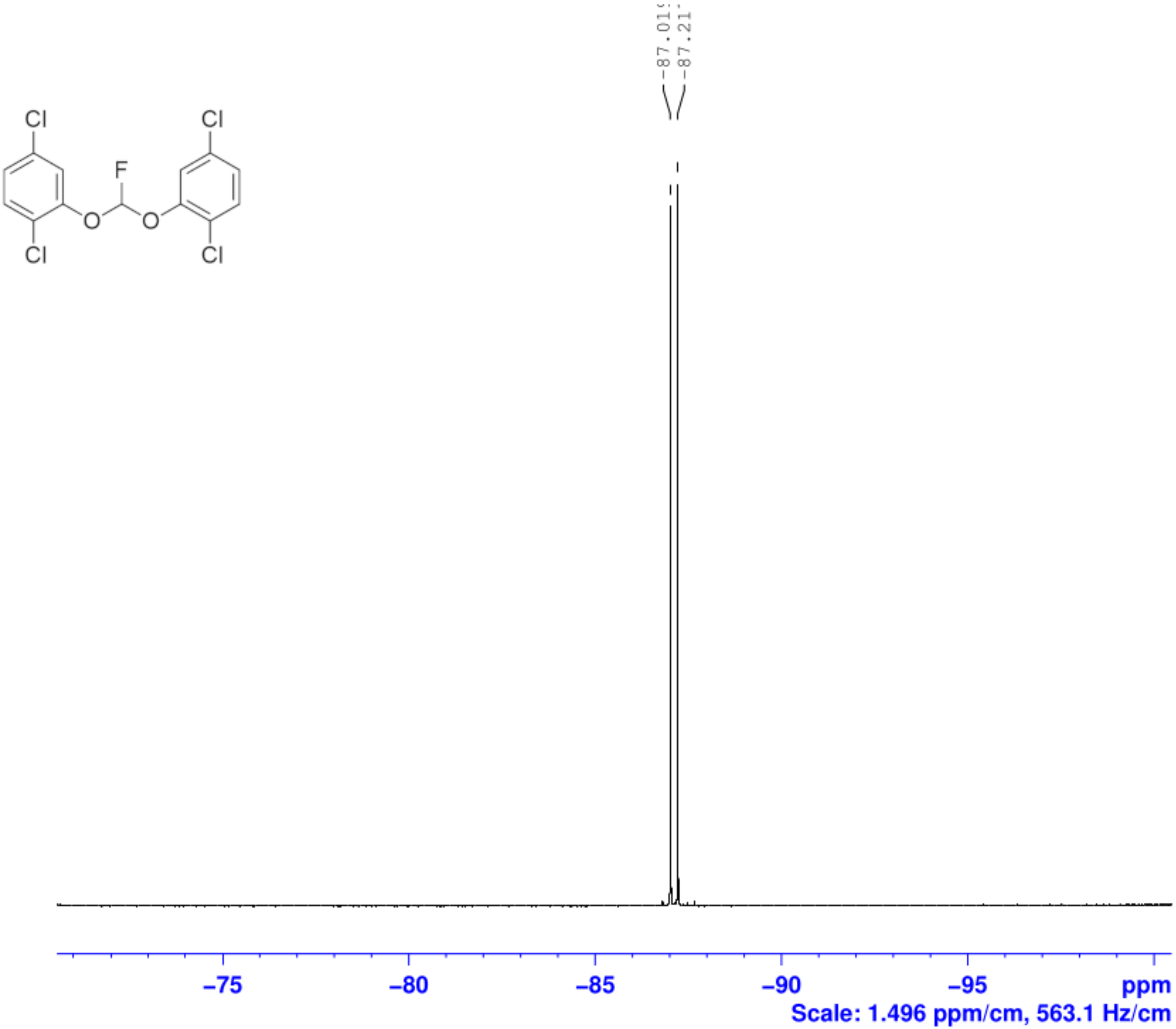

NAME	P7K26.1.21
EXPNO	6
PROCNO	1
Date_	20210126
Time	10.06
INSTRUM	spect
PROBHD	5 mm PABBO BB-
PULPROG	zg30
TD	32768
SOLVENT	DMSO
NS	24
DS	0
SWH	11261.262 Hz
FIDRES	0.343666 Hz
AQ	1.4549491 se
RG	1625.5
DW	44.400 us
DE	6.32 us
TE	299.9 K
D1	0.10000000 se
TD0	1

======== CHANNEL f1 ======

NUC1	19F
P1	7.90 us
PL1	0.00 d
SFO1	376.4381370 MH
SI	32768
SF	376.4703301 MH
WDW	EM
SSB	0
LB	0.50 Hz
GB	0
PC	1.00

HMBC

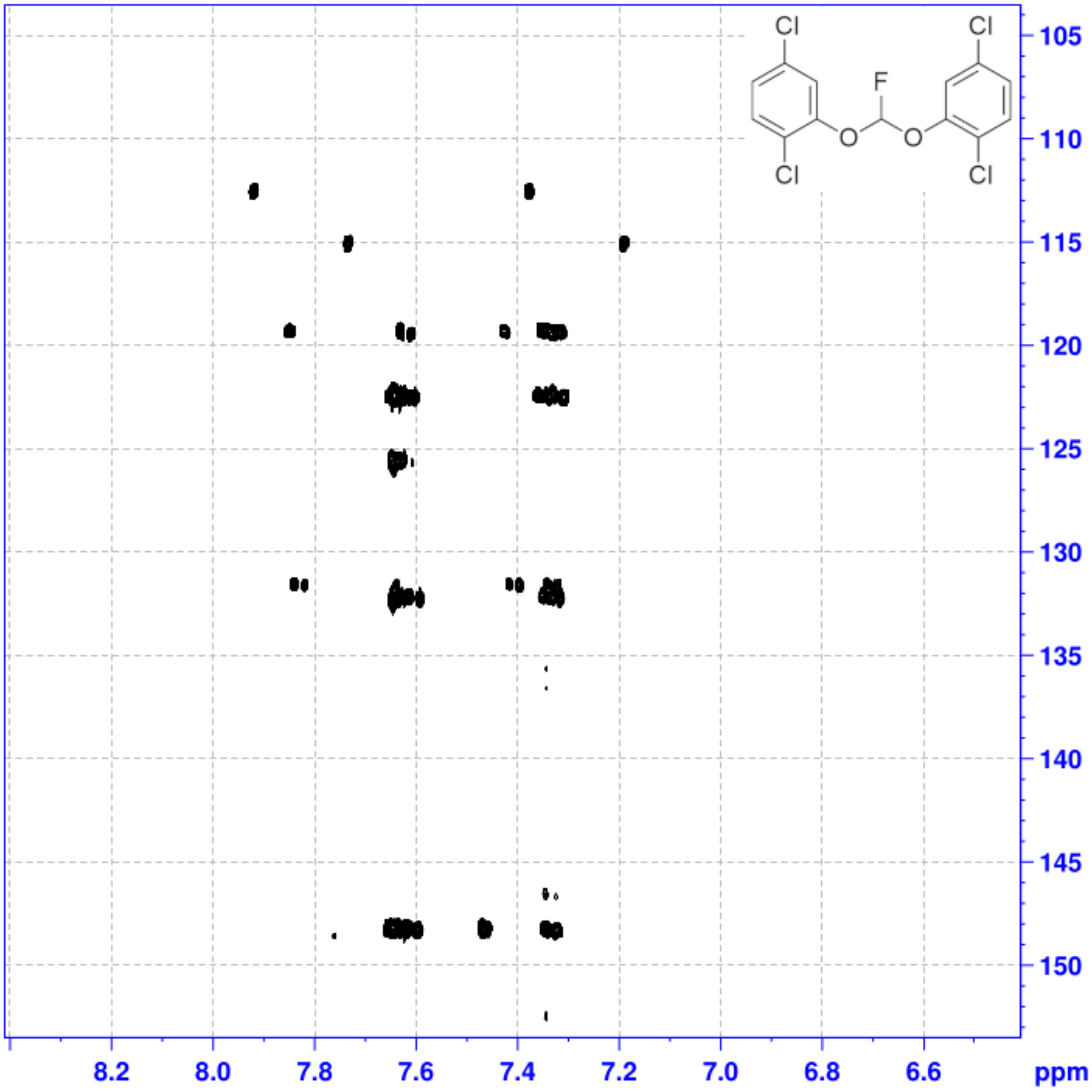

NAME	P7K26.1.21
EXPNO	11
PROCNO	1
Date_	20210126
Time	10.56
INSTRUM	spect
PROBHD	5 mm PABBO BB-
PULPROG	hmbcgplpndqf
TD	1024
SOLVENT	DMSO
NS	8
DS	2
SWH	801.282 Hz
FIDRES	0.782502 Hz
AQ	0.6390260 sec
RG	46341
DW	624.000 usec
DE	241.25 usec
TE	299.2 K
CNST2	145.0000000
CNST13	8.0000000
D0	0.00000300 sec
D1	1.00000000 sec
D2	0.00344828 sec
D6	0.06250000 sec
D16	0.00020000 sec
IN0	0.00009940 sec
======== CHANNEL f1 ========	
NUC1	1H
P1	10.10 usec
P2	20.20 usec
PL1	0.00 dB
SFO1	400.1029607 MHz
======== CHANNEL f2 ========	
NUC2	13C
P3	9.20 usec
PL2	0.00 dB
SFO2	100.6182041 MHz
====== GRADIENT CHANNEL =====	
GPNAM1	sine.100
GPNAM2	sine.100
GPNAM3	sine.100
GPZ1	50.00 %
GPZ2	30.00 %
GPZ3	40.10 %
P16	1000.00 usec
ND0	2
TD	128
SFO1	100.6182 MHz
FIDRES	39.303986 Hz
SW	50.000 ppm
FnMODE	QF
SI	1024
SF	400.0999959 MHz
WDW	QSINE
SSB	0
LB	0.00 Hz
GB	0
PC	1.00
SI	512
MC2	QF
SF	100.6052769 MHz
WDW	QSINE
SSB	0
LB	0.00 Hz
GB	0

HMQC

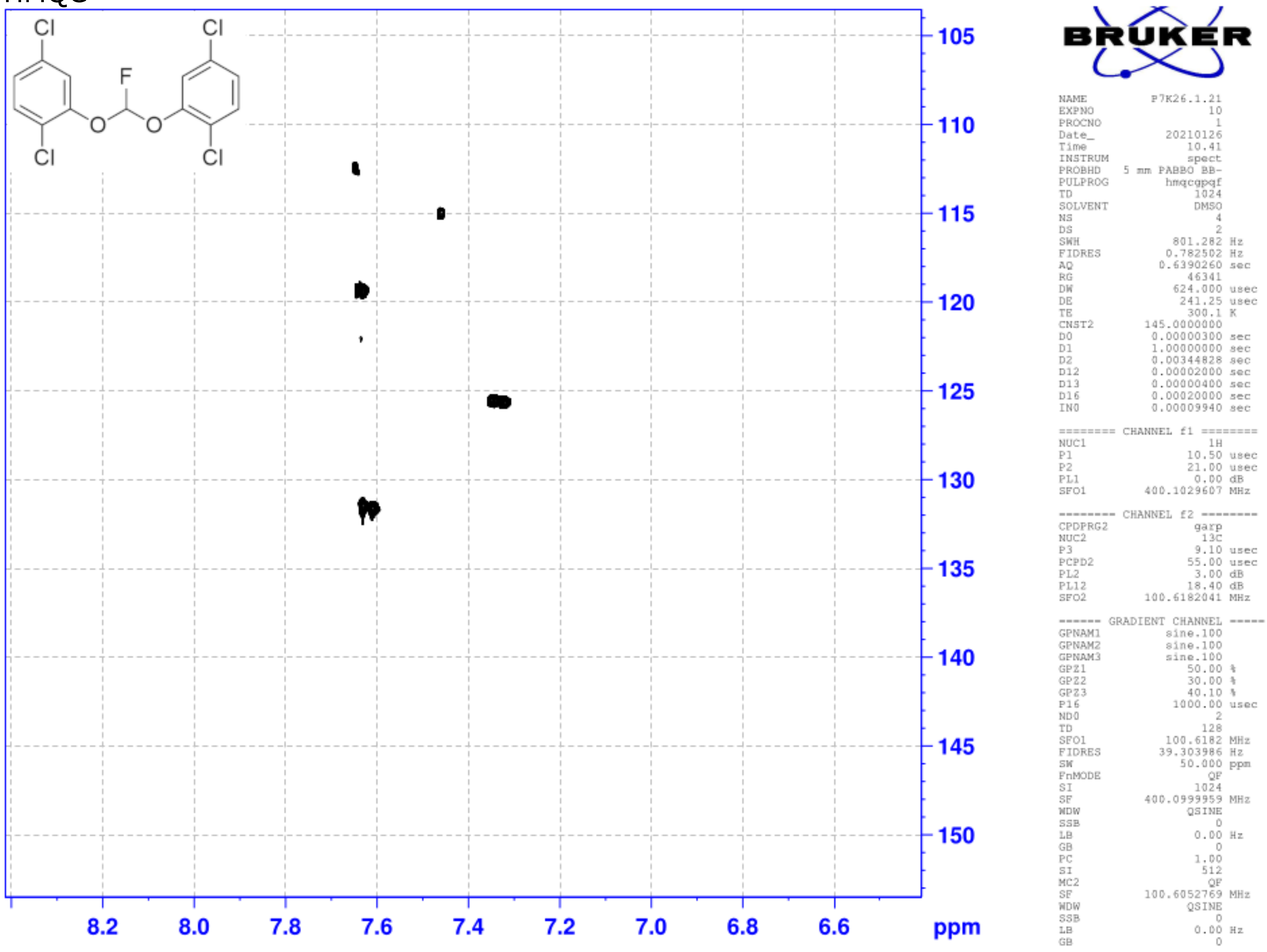
Cl
Cl
F
O
O
Cl
Cl
105
110
115
120
125
130
135
140
145
150
8.2
8.0
7.8
7.6
7.4
7.2
7.0
6.8
6.6
ppm
BRUKER
NAME P7K26.1.21
EXPNO 10
PROCNO 1
Date_ 20210126
Time 10.41
INSTRUM spect
PROBHD 5 mm PABBO BB-
PULPROG hmqcgpqf
TD 1024
SOLVENT DMSO
NS 4
DS 2
SWH 801.282 Hz
FIDRES 0.782502 Hz
AQ 0.6390260 sec
RG 46341
DW 624.000 usec
DE 241.25 usec
TE 300.1 K
CNST2 145.0000000
D0 0.00000300 sec
D1 1.00000000 sec
D2 0.00344828 sec
D12 0.00002000 sec
D13 0.00000400 sec
D16 0.00020000 sec
IN0 0.00009940 sec
======== CHANNEL f1 ========
NUC1 1H
P1 10.50 usec
P2 21.00 usec
PL1 0.00 dB
SFO1 400.1029607 MHz
======== CHANNEL f2 ========
CPDPRG2 garp
NUC2 13C
P3 9.10 usec
PCPD2 55.00 usec
PL2 3.00 dB
PL12 18.40 dB
SFO2 100.6182041 MHz
====== GRADIENT CHANNEL =====
GPNAM1 sine.100
GPNAM2 sine.100
GPNAM3 sine.100
GPZ1 50.00 %
GPZ2 30.00 %
GPZ3 40.10 %
P16 1000.00 usec
ND0 2
TD 128
SFO1 100.6182 MHz
FIDRES 39.303986 Hz
SW 50.000 ppm
FnMODE QF
SI 1024
SF 400.0999959 MHz
WDW QSINE
SSB 0
LB 0.00 Hz
GB 0
PC 1.00
SI 512
MC2 QF
SF 100.6052769 MHz
WDW QSINE
SSB 0
LB 0.00 Hz
GB 0

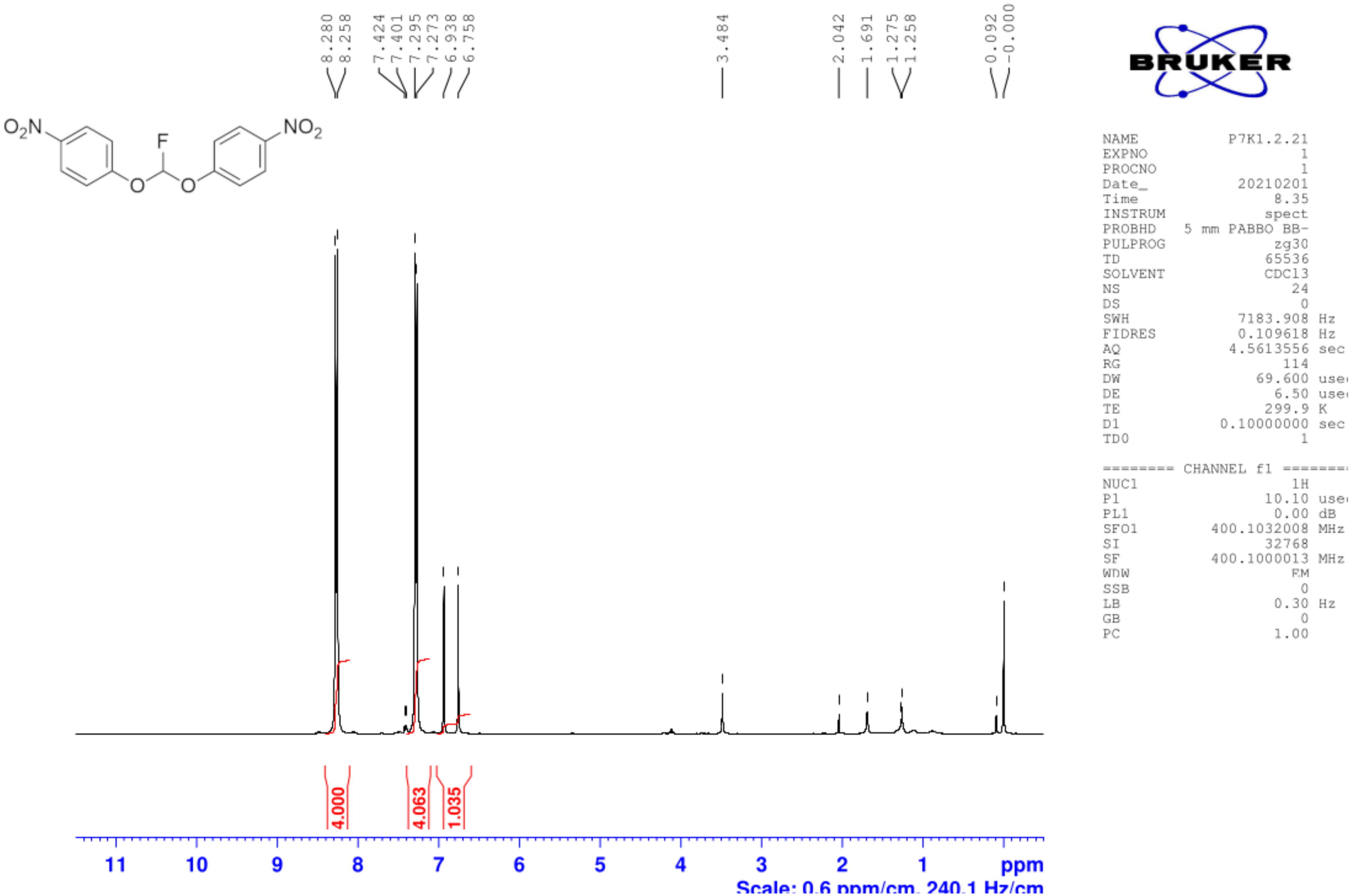
8.280
8.258
7.424
7.401
7.295
7.273
6.938
6.758
3.484
2.042
1.691
1.275
1.258
0.092
-0.000
O_2N
F
O
O
NO_2
4.000
4.063
1.035
11
10
9
8
7
6
5
4
3
2
1
ppm
Scale: 0.6 ppm/cm, 240.1 Hz/cm
BRUKER
NAME P7K1.2.21
EXPNO 1
PROCNO 1
Date_ 20210201
Time 8.35
INSTRUM spect
PROBHD 5 mm PABBO BB-
PULPROG zg30
TD 65536
SOLVENT CDCl3
NS 24
DS 0
SWH 7183.908 Hz
FIDRES 0.109618 Hz
AQ 4.5613556 sec
RG 114
DW 69.600 use
DE 6.50 use
TE 299.9 K
D1 0.10000000 sec
TD0 1
======== CHANNEL f1 ========
NUC1 1H
P1 10.10 use
PL1 0.00 dB
SFO1 400.1032008 MHz
SI 32768
SF 400.1000013 MHz
WDW EM
SSB 0
LB 0.30 Hz
GB 0
PC 1.00

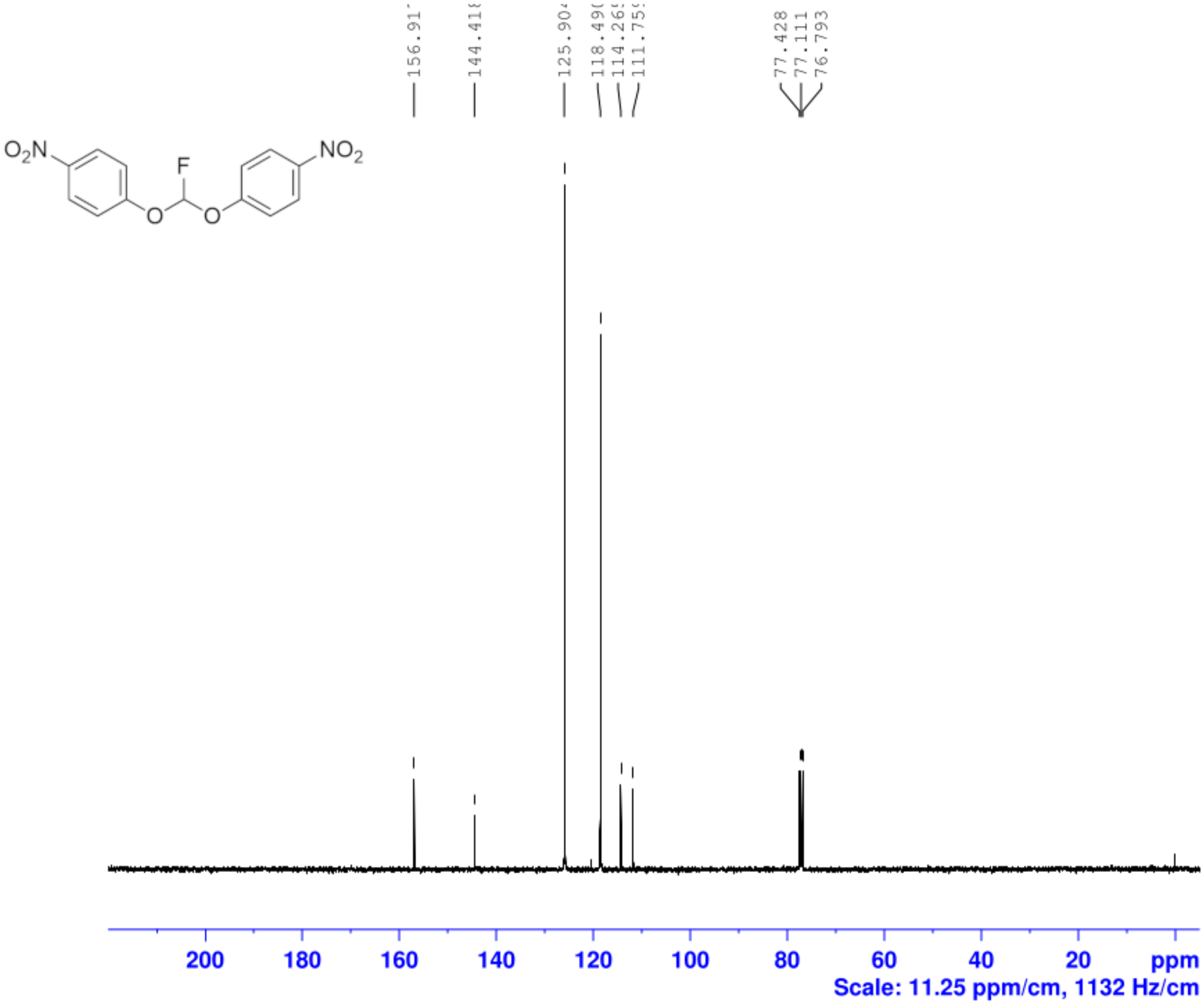

NAME	P7K1.2.21
EXPNO	3
PROCNO	1
Date_	20210201
Time	8.39
INSTRUM	spect
PROBHD	5 mm PABBO BB-
PULPROG	zgdc30
TD	32768
SOLVENT	CDCl3
NS	1000
DS	0
SWH	24154.590 Hz
FIDRES	0.737140 Hz
AQ	0.6783476 se
RG	71.8
DW	20.700 us
DE	29.57 us
TE	300.5 K
D1	0.10000000 se
D11	0.03000000 se
TD0	1
======== CHANNEL f1 ======	
NUC1	13C
P1	9.20 us
PL1	3.00 dB
SFO1	100.6152865 MH
======== CHANNEL f2 ======	
CPDPRG2	waltz16
NUC2	1H
PCPD2	80.00 us
PL2	0.00 dB
PL12	18.00 dB
SFO2	400.1009202 MH
SI	32768
SF	100.6052219 MH
WDW	EM
SSB	0
LB	2.00 Hz
GB	0
PC	1.00

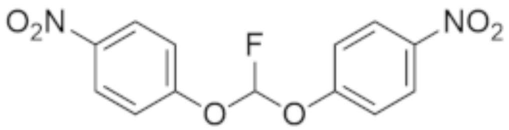

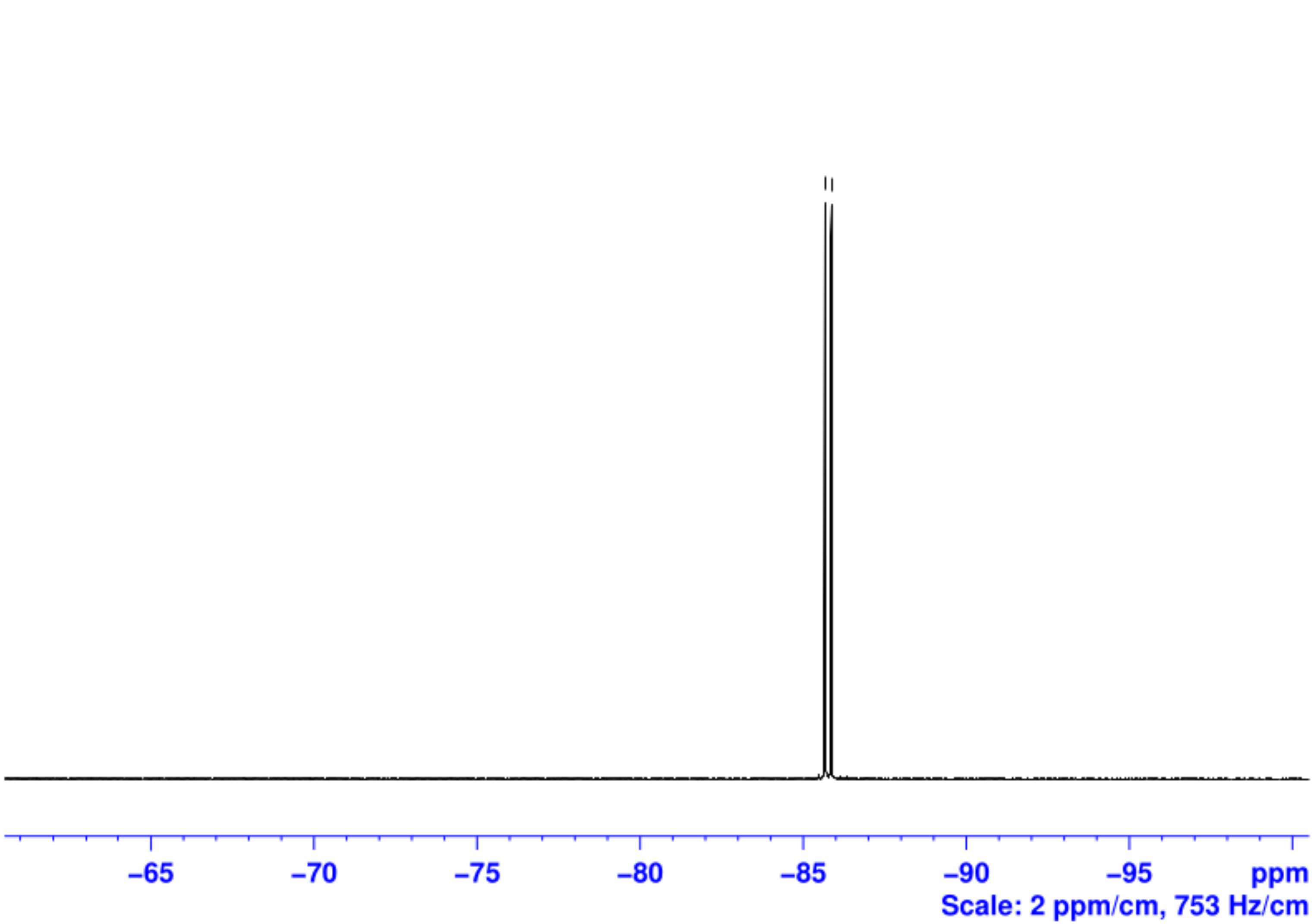

BRUKER

NAME	P7K1.2.21
EXPNO	2
PROCNO	1
Date_	20210201
Time	8.36
INSTRUM	spect
PROBHD	5 mm PABBO BB-
PULPROG	zg30
TD	32768
SOLVENT	CDCl3
NS	8
DS	0
SWH	15060.241 Hz
FIDRES	0.459602 Hz
AQ	1.0879476 se
RG	2580.3
DW	33.200 us
DE	6.32 us
TE	299.9 K
D1	0.10000000 se
TD0	1
======== CHANNEL f1 ======	
NUC1	19F
P1	7.90 us
PL1	0.00 dB
SFO1	376.4400194 MH
SI	32768
SF	376.4703301 MH
WDW	EM
SSB	0
LB	0.50 Hz
GB	0
PC	1.00

HMQC

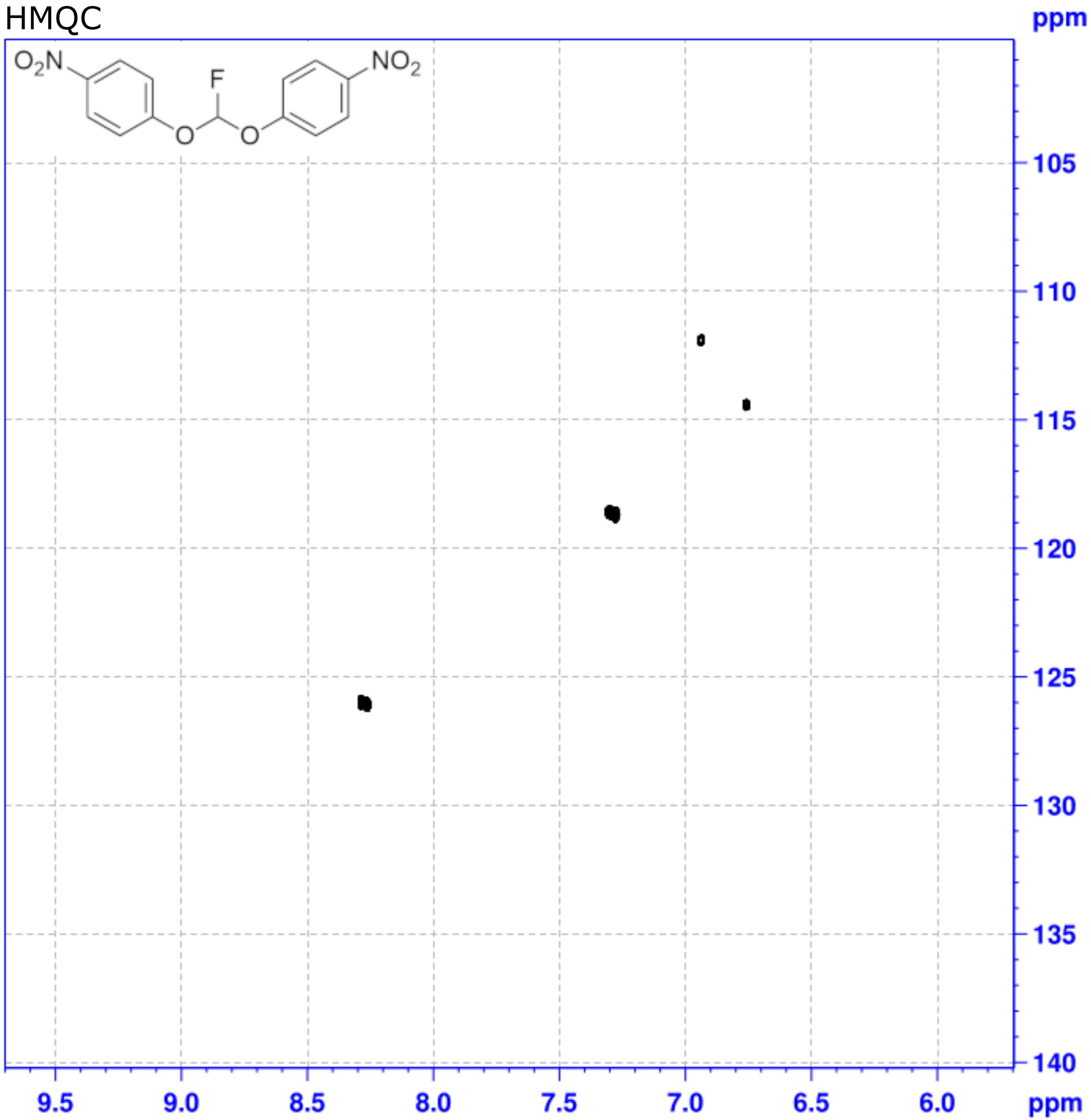

NAME	P7K1.2.21
EXPNO	5
PROCNO	1
Date_	20210201
Time	9.11
INSTRUM	spect
PROBHD	5 mm PABBO BB-
PULPROG	hmqcgpqf
TD	1024
SOLVENT	CDCl3
NS	4
DS	2
SWH	1602.564 Hz
FIDRES	1.565004 Hz
AQ	0.3195380 sec
RG	20642.5
DW	312.000 usec
DE	241.25 usec
TE	300.2 K
CNST2	145.0000000
D0	0.00000300 sec
D1	1.00000000 sec
D2	0.00344828 sec
D12	0.00002000 sec
D13	0.00000400 sec
D16	0.00020000 sec
IN0	0.00012425 sec

======== CHANNEL f1 ========

NUC1	1H
P1	10.50 usec
P2	21.00 usec
PL1	0.00 dB
SFO1	400.1030808 MHz

======== CHANNEL f2 ========

CPDPRG2	garp
NUC2	13C
P3	9.10 usec
PCPD2	55.00 usec
PL2	3.00 dB
PL12	18.40 dB
SFO2	100.6172986 MHz

====== GRADIENT CHANNEL =====

GPNAM1	sine.100
GPNAM2	sine.100
GPNAM3	sine.100
GPZ1	50.00 %
GPZ2	30.00 %
GPZ3	40.10 %
P16	1000.00 usec
ND0	2
TD	128
SFO1	100.6173 MHz
FIDRES	31.442905 Hz
SW	40.000 ppm
FnMODE	QF
SI	1024
SF	400.1000009 MHz
WDW	QSINE
SSB	0
LB	0.00 Hz
GB	0
PC	1.00
SI	512
MC2	QF
SF	100.6052043 MHz
WDW	QSINE
SSB	0
LB	0.00 Hz
GB	0
